# ChemSpaceAL: An Efficient Active Learning Methodology Applied to Protein-Specific Molecular Generation

**Published:** 2023-09-11

**Authors:** Gregory W. Kyro, Anton Morgunov, Rafael I. Brent, Victor S. Batista

**Affiliations:** Yale University

## Abstract

The incredible capabilities of generative artificial intelligence models have inevitably led to their application in the domain of drug discovery. It is therefore of tremendous interest to develop methodologies that enhance the abilities and applicability of these powerful tools. In this work, we present a novel and efficient semi-supervised active learning methodology that allows for the fine-tuning of a generative model with respect to an objective function by strategically operating within a constructed representation of the sample space. In the context of targeted molecular generation, we demonstrate the ability to fine-tune a GPT-based molecular generator with respect to an attractive interaction-based scoring function by strategically operating within a chemical space proxy, thereby maximizing attractive interactions between the generated molecules and a protein target. Importantly, our approach does not require the individual evaluation of all data points that are used for fine-tuning, enabling the incorporation of computationally expensive metrics. We are hopeful that the inherent generality of this methodology ensures that it will remain applicable as this exciting field evolves. To facilitate implementation and reproducibility, we have made all of our software available through the open-source ChemSpaceAL Python package.

## Graphical Abstract

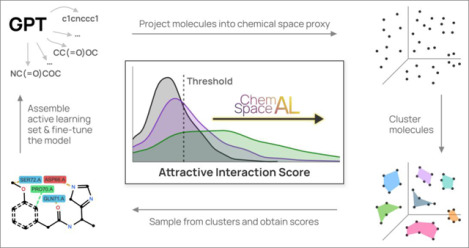

## 1. Introduction

The vast majority of pharmaceutical drugs function by targeting a specific protein.^1^ A popular method for discovering effective drugs is virtual screening, a computational approach designed to brute-force search a molecular library to identify candidates with high likelihoods of binding to a target protein. The efficacy of virtual screening pipelines is intrinsically tied to the comprehensiveness of the libraries that they screen, and it is therefore beneficial to enrich these libraries with novel drug-like small molecules. While the total number of chemical compounds synthesized to date is approximately 10^8^, it is estimated that there are between 10^23^ and 10^60^ theoretically feasible drug-like compounds.^2^ Given the challenge of screening on the order of 10^23^ drugs in a computationally feasible manner, it is advantageous to align the design of new molecules to a specific protein of interest, thereby reducing the total search space while simultaneously increasing the probability of generating molecules that will successfully bind.

Artificial intelligence (AI) has recently demonstrated remarkable capabilities in distinct domains, ranging from natural language processing with Generative Pretrained Transformer (GPT)-4 to protein structure prediction with AlphaFold.^3,4^ Molecular design methods powered by generative AI have been gaining tremendous attention in recent years, with numerous models based on architectures such as recurrent neural networks (RNNs),^5–27^ generative adversarial networks (GANs),^28–40^ autoencoders (AEs),^41–61^ and transformers.^62–69^ RNNs process sequential data by integrating information from current and previous sequence elements, updating their internal state with each iteration. In the case of GANs, a generative component contends with a discriminator trained to distinguish between real and generated data points. This setup enables the generation of new molecules that closely resemble the training distribution with respect to any criteria discernable by the discriminator. AEs, which often make use of RNNs as subcomponents, learn to compress input data into a lower-dimensional latent space and subsequently reconstruct the original input from the latent representation. AEs are able to generate novel molecules by decoding vectors from a regularized latent space. There have also been several AE variants for molecular design, such as training an AE to learn the latent representations of molecules, followed by training a GAN on these latent representations to generate novel latent vectors corresponding to new molecules.^70,71^

The transformer architecture represents one of the most significant advancements in AI, revolutionizing the way models understand and generate sequential data.^72^ Central to this innovation is the self-attention mechanism, where an embedding of each token (i.e., sequence element) interacts with embeddings of every other token, yielding weighting factors that establish the strengths of the connections between them. This mechanism enables the model to develop extremely rich internal representations, which have been successfully leveraged for tasks such as language translation,^72^ text classification,^73^ and next-token prediction.^3^ Many groups have investigated the use of transformer units in the field of de novo molecular generation.^62–69^

Many generative models in molecular design, such as those based on RNNs and transformers, utilize sequential data representations. This approach necessitates encoding inherently multi-dimensional molecules as one-dimensional sequences. The predominant method for this conversion is through the simplified molecular-input line-entry system (SMILES), which involves linear representations of molecular structures that can be reverted to their corresponding molecular connectivity graphs.^74^ There is also interest in direct graph-based representations, and several groups have explored molecular generative models based on graph neural networks.^75–82^

In supervised machine learning (ML), the model optimizes its parameters via backpropagation, which involves calculation of the gradient of a loss function with respect to the model’s parameters. This gradient indicates the direction for each parameter update, ensuring that each iterative refinement moves the model toward more accurate predictions. Reinforcement learning (RL) has emerged as a prominent alternative across various AI domains, including molecular generation. Unlike supervised learning, RL often employs a reward function that is not differentiable with respect to the model’s parameters, preventing the application of backpropagation. Instead, the model in RL is conceptualized as an agent that operates within a defined environment, selecting actions and receiving rewards or penalties based on the outcomes of these actions. In the context of molecular generation, different RL strategies have been implemented using several of the architectures previously mentioned, often involving the evaluation of specific properties of generated molecules. This approach provides the model with quantitative feedback derived from these evaluations, allowing the model to update its parameters accordingly. Research continues into developing and optimizing RL frameworks and strategies for molecular generation beyond the scope of traditional ML architectures.^83–85^

While RL employs quantitative rewards and penalties to update the model’s parameters, active learning (AL) refines the model by further training with selectively chosen data points. This refinement typically requires less data, a reduced learning rate, and fewer training epochs compared to the initial pretraining, ensuring that the model retains its broad domain knowledge while narrowing its focus toward a more precise objective. One significant advantage of AL over RL is the potential to utilize unsupervised or semi-supervised learning to curate fine-tuning training sets without performing resource-intensive evaluations of each generated data point. Consequently, a well-constructed sampling algorithm can expose the model to data that span the ideal search space while minimizing computational cost. However, it is crucial to note that if the data chosen for fine-tuning are not judiciously selected, the process can be ineffectual or even detrimental. Hence, the design of the sampling algorithm is paramount in AL methodologies.

Although the application of AL methods to molecular generation is still in its early stages, several noteworthy studies have been published. Ghaemi et al. enhanced an RNN by calculating the Quantitative Estimate of Drug-Likeness (QED)^86^ and only retaining molecules with scores that exceed a fixed threshold.^25^ In another study, Westermayr et al. refined the outputs of G-SchNet,^89^ a model designed for generating three-dimensional molecular structures, by computing a defined property of interest for all generated molecules, and then selecting the molecules with a value greater than one standard deviation above/below the mean for maximization/minimization tasks, respectively.^91^ Filella-Merce et al. performed a two-tiered AL strategy using an AE, where the inner loop of their system filters molecules based on criteria such as QED, synthetic accessibility, and similarity to previously retained molecules, and the outer loop operates only on those molecules that pass the inner loop’s filters, further refining the AL data based on in silico binding affinity to a specific protein.^53^ Notably, a common pattern across these approaches is the calculation of certain properties for every molecule and evaluation of each molecule against set selection criteria. While effective in cases where it is computationally tractable, this approach can be limiting when optimizing over more complex molecular properties that require expensive calculations.

In this work, we present a novel and efficient semi-supervised AL methodology that can align a generative model to a specified objective function without the need to evaluate each data point individually. We demonstrate the efficacy of this method by fine-tuning a molecular generator toward a protein target.

## 2. ChemSpaceAL Methodology and Related Theory

### 2.1 Overview

Our demonstration of the ChemSpaceAL methodology applied to molecular generation proceeds as follows:

1. Pretrain our GPT-based model on millions of SMILES strings so that it learns the rules and structure of SMILES notation
2. Use the trained model to generate a large number of unique SMILES strings
3. Calculate molecular descriptors containing information about molecular topology, physical properties, and presence of functional groups for each generated molecule
4. Project the generated SMILES strings into our chemical space proxy
5. Use k-means clustering on the generated molecules to group those with similar properties
6. Sample a small number of molecules from each cluster and dock each of them to a protein target (e.g., the HNH domain of Cas9)
7. Evaluate the top-ranked pose of each protein-ligand complex with a heuristic attractive interaction-based scoring function
8. Map the scores back to the original clusters and sample from the clusters proportionately to the cluster scores, combine the sampled molecules with the evaluated molecules whose scores meet or exceed our specified threshold, and include all of them in the AL training set
9. Refine our model by fine-tuning it with the AL training set
  *) Repeat steps (2) – (9) for multiple iterations, guiding the generation toward regions of chemical space that contain molecules with higher scores

### 2.2 GPT Architecture

The GPT model that we employ is based on the transformer architecture introduced in the revolutionary paper, “Attention is All You Need”.^72^ Within the framework of the transformer architecture, the encoder processes input data into a sequence of context-rich vectors, while the decoder utilizes this contextual information to generate output data. Both of these components utilize a self-attention mechanism, which enables the model to selectively focus on distinct parts of the input sequence at each computational step. The technical difference between the encoder and decoder parts of the transformer model is that the decoder ensures that the prediction for a particular token only depends on the preceding tokens, while each token in the encoder can attend to all other tokens in the sequence. Our GPT model is constructed as a series of transformer decoder blocks. This approach is appropriate for tasks that require generating novel sequential data such as SMILES strings.

The forward pass of our GPT model begins by dividing each SMILES string into distinct units known as tokens, processing each token with embedding layers, and combining these embeddings to form a vector representation of each token. These embedded vectors are then sequentially passed through a series of transformer decoder blocks, each comprised of a self-attention layer and a feed-forward network, with additional structural elements to enhance learning. The final result is a sequence of vectors, each corresponding to a position in the output SMILES string, where the elements of each vector represent probabilities for each token in the vocabulary. This high-level overview sets the stage for a more detailed discussion of the individual components.

#### Embeddings:

Initially, a vocabulary comprising all of the unique tokens in the training data is constructed. For any given SMILES string in the input data, the input tokens undergo three distinct processing methods: token, positional, and type embeddings. The token embedding maps each token in the input sequence to a learnable vector representation, allowing the model to learn an optimal high-dimensional characterization for each token. Similarly, the positional embedding maps each input token to a learnable vector based on its position in the sequence. The type embedding layer uniformly assigns a constant bias to all embeddings of each input sequence. The sum of these three embeddings is passed through a dropout layer, setting 10% of its scalar components to 0. This embedding process transforms the input tokens into a form more suitable for the downstream modeling process.

#### Transformer Decoder Stack:

For each token in the input sequence, the resulting embedding is passed to the first transformer decoder block, which begins with layer normalization, a process that adjusts and scales each embedding to have a mean of 0 and a standard deviation of 1. A self-attention mechanism is then applied to the normalized embedding, using learned matrices to linearly transform the embedding into three different vectors known as the query, key, and value vectors:

(1) $$q_{i}=W_{q}\times e_{i}$$

(2) $$k_{i}=W_{k}\times e_{i}$$

(3) $$v_{i}=W_{v}\times e_{i}$$

where $W_{q}$, $W_{k}$, and $W_{v}$ are learned weight matrices that transform each input embedding, represented by $e_{i}$, into the corresponding query, key, and value vectors. The dot products of the query and each key vector are then scaled according to the dimensionality of the key vectors and passed through a softmax function, transforming them into a probability distribution to serve as attention weights. Finally, the attention scores are used to generate a weighted sum of the value vectors, as shown in the following equation:

(4) $$e^{\prime }{}_{i}=V\times \text{softmax}\left(\begin{array}{c} \frac{q_{i}\cdot K_{1}}{\sqrt{{\text{d}}_{k}}}\\ \cdots \\ \frac{q_{i}\cdot k_{L}}{\sqrt{{\text{d}}_{k}}} \end{array}\right)$$

Here, $e^{\prime }{}_{i}$ represents the output of the attention mechanism at position *i* in the sequence, V is the value matrix whose *j*^th^ column is the value vector corresponding to the embedding at position *j* in the sequence, d_*k*_ denotes the dimensionality of the key vectors, and *L* represents the length of the entire sequence. This operation amplifies the information from value vectors corresponding to higher attention weights (i.e., tokens that are more relevant to the current query), while suppressing the information from less relevant value vectors.

In practice, the self-attention mechanism is executed multiple times in parallel through what is known as *multi-head* attention. Each head (i.e., execution) uses its own set of learned linear transformations to generate query, key, and value vectors for all tokens in the sequence for each item in the batch, allowing the model to simultaneously focus on different aspects of the input across the various heads. The outputs from all attention heads are then concatenated and passed through a learned linear transformation to generate the final output of the multi-head attention mechanism.

A residual connection is a shortcut that skips one or more layers and allows the original input to be added directly to the output of those layers. This technique aids in training deeper networks by mitigating the vanishing gradient problem, where the gradients become too small for the network to learn effectively. In the context of GPT models, a residual connection is made by adding the input of the attention mechanism to the output. This sum is then processed using layer normalization, and the transformed embeddings are passed through a feed-forward network using the equation:

(5) $$H=\text{Dropout}\left(W_{2}\times \text{GELU}\left(W_{1}\times E^{\prime }+b_{1}\right)+b_{2}\right)$$

where H is the output of the feed-forward network, E′ represents the matrix whose columns are the transformed embeddings, and $W_{1}$ (shape: 1024×256), **b**_**1**_ (shape: 1024), $W_{2}$ (shape: 256×1024), and $b_{2}$ (shape: 256) represent the weight matrices and bias vectors of the two linear layers. GELU, or Gaussian Error Linear Unit, is an activation function used to introduce non-linearity into the model. A residual connection is established by summing the input to this feed-forward network with the output.

This entire process is repeated for additional decoder blocks, and the output of the final decoder block is processed with layer normalization. The normalized output is then passed through a learned linear transformation with bias to map the embeddings to the output vocabulary size, and the resulting vectors are processed with softmax to generate the output probabilities at each position in the sequence.

### 2.3 GPT Parameters and Pretraining

Our GPT model is composed of eight transformer decoder blocks, each of which contains eight attention heads, and it embeds inputs into a 256-dimensional space. We pretrain our model using a dataset containing millions of SMILES strings corresponding to valid chemical structures. The objective during pretraining is to train the model to correctly predict the next token in the input sequence given the current and preceding tokens. This task encourages the model to grasp the syntax and structure of SMILES notation as a means of understanding molecular structures. The training process utilizes cross-entropy loss with L2 regularization applied to the linear layers using λ=0.1, and the SophiaG optimizer^87^ with β_1_=0.965, β_2_=0.99 and ρ=0.04. The learning rate warms up to 3×10^−4^ during the first 10% of tokens, then decays to 3×10^−5^ using cosine decay. Dropout with a probability of 10% is applied after each feed-forward network except for the output layer to mitigate overfitting, and gradient clipping is used in conjunction with layer normalization to stabilize the optimization process and prevent exploding gradients. All weights are initialized according to a Gaussian distribution with a mean of 0 and a standard deviation of 0.02 except for weights involved in layer normalization, which are initialized to 1, and bias parameters, which are initialized to 0. During pretraining, the model is trained with a batch size of 512 for 30 epochs (Supporting Information, Figures S1.1–S1.2).

### 2.4 Dataset Collection and Preprocessing

#### Data Collection:

We curate a pretraining set by gathering SMILES strings from multiple datasets:

- **ChemBL 33**: Contains about 2.4 million bioactive molecules with drug-like properties.^88^
- **GuacaMol v1**: Comprises about 1.6 million molecules derived from ChemBL 24 that have been synthesized and tested against biological targets.^89^
- **MOSES**: Encompasses about 1.8 million molecules selected from Zinc 15^90^ to maximize internal diversity and suitability for medicinal chemistry.^91^
- **BindingDB (08–2023)**: Includes about 1.2 million unique small molecules bound to proteins.^92^

We combine all of the SMILES strings from these sources, filter out the strings that are identified as invalid by the RDKit molecular parser,^93^ and remove any duplicate strings to prevent redundancy. The resulting combined dataset contains 5,622,772 unique and valid SMILES strings, with a vocabulary of 196 unique tokens.

#### Tokenization Process:

The SMILES strings are tokenized, meaning that they are divided into distinct units, each representing a specific element or feature. Some tokens contain a single character (e.g., “C” for carbon), while others consist of multiple characters (e.g., “Br” for bromine). This tokenization procedure allows the model to capture the syntax of SMILES notation, facilitating the training of the model to recognize patterns and relationships in chemical structures. We find that 106 tokens are represented in the dataset less than 100 times, and an additional 42 tokens occur less than 1,000 times. To reduce the size of our vocabulary (from 196 to 48), we remove all SMILES strings containing at least one token that appears less than 1,000 times in our dataset. Most of the SMILES strings excluded contain rare transition metals or isotopes.

#### Data Preprocessing:

The longest SMILES string in the dataset has 1,503 tokens, while 99% of the strings in the dataset have 133 or fewer tokens. We impose a SMILES string length cutoff of 133, and remove any string from the dataset whose length is greater than the cutoff. All remaining SMILES strings are then augmented with a start token “!”, an end-of-sequence token “~”, and are extended to the length of the longest SMILES string in the dataset using a padding token “<”. The resulting pretraining dataset contains 5,539,765 SMILES strings, which are randomly split into training (5,262,776 entries; 95.0%) and validation (276,989 entries; 5.0%) sets.

### 2.5 Constructing Our Chemical Space Proxy

In order to select beneficial molecules to be in our AL training set without having to evaluate each one, we need a way to relate molecules that have been scored to those that have not. To achieve this goal, we construct a proxy for chemical space that is predicated on molecular properties, allowing us to operate within a space where nearby molecules share similar features. We first calculate the full set of molecular descriptors available through RDKit’s CalcMolDescriptors method for each molecule in the pretraining set, encompassing a wide range of molecular properties including structural, topological, geometrical, electronic and thermodynamic characteristics. Among these 209 descriptors, 13 return NaN (not a number) or infinity for at least one SMILES string in the dataset and consequently are discarded, resulting in 196 descriptors (Supporting Information, Tables S2.1–S2.3). We then perform Principal Component Analysis (PCA) using these descriptors for all molecules in the pretraining set, and find that 99% of the variance is explained by the first 113 principal components. We use the first 120 principal components to construct a 120-dimensional space, which is used throughout the pipeline as our chemical space proxy. At each iteration, we generate 100,000 unique molecules and project them into this space.

### 2.6 Clustering and Sampling from Chemical Space

After constructing our chemical space proxy and projecting generated molecules into this space, we need a way of grouping molecules that likely have similar scores. We show that position in our constructed representation of chemical space correlates with the objective function (see Results and Discussion), and therefore group data points according to proximity in the chemical space proxy.

#### Clustering:

Within our chemical space proxy, we utilize k-means clustering to group molecules that exhibit similar chemical properties, with k set to 100. We employ the k-means++ initialization algorithm, where the first centroid is selected randomly and subsequent centroids are iteratively chosen with a probability proportional to their squared distance from the nearest existing centroid. To mitigate the potential for poor initialization, we perform k-means 100 times, seeking to minimize k-means loss (i.e., the sum of squared distances from each data point to the centroid of its cluster) and variance in cluster sizes. Initially, we sort each of the 100 clusterings by k-means loss, and take the five clusterings with the lowest loss, thereby preserving those with more compact clusters. Of these five, we select the clustering with the lowest variance in cluster size for use in the following stages of the methodology.

#### Sampling:

After clustering the generated molecules in our chemical space proxy, we randomly select 10 molecules from each cluster that contains at least 10 molecules, and select all of the molecules from any cluster that contains less than 10 molecules. We then randomly sample from the clusters with more than 10 molecules until we achieve a set of 1,000 molecules.

### 2.7 Docking to a Protein Target and Scoring Protein-Ligand Pairs

Having strategically selected a set of 1,000 molecules, the next steps in our methodology are to dock each molecule to a specific protein target and score the top-ranked pose for each complex. This process allows us to estimate attractive interactions between each selected molecule and the protein target.

#### Docking to a Protein Target:

Obtaining a docking pose for each protein-ligand pair is necessary for deriving the protein-ligand interaction fingerprint that we use for scoring. Docking is conducted using the DiffDock software^94^ with the HNH domain of Cas9 (PDB ID: 6O56)^95^ as our protein target. We select HNH as our protein target because of its moderate size and critical role in the catalytic activity of the CRISPR/Cas9 system. These characteristics make it a computationally manageable candidate that is also relevant to enzymatic biological processes, which are largely influenced by protein-ligand interactions. During the docking inference stage, we utilize 20 inference steps, 10 samples for each complex, and a batch size of 6. It should be noted that since DiffDock is a diffusion generative model, it is inherently stochastic in nature. We select the top-ranked docking pose for each protein-ligand pair, which is subsequently used for scoring.

#### Scoring Protein-Ligand Complexes:

We utilize a heuristic scoring function that considers various types of attractive contributions to protein-ligand binding using the prolif (Protein-Ligand Interaction Fingerprints) software package^96^ and handpicked weights for each interaction type: hydrophobic interactions are scored at 2.5; hydrogen-bond interactions at 3.5; ionic interactions at 7.5; interactions between aromatic rings and cations at 2.5; Van der Waals interactions at 1.0; halogen-bond interactions at 3.0; face-to-face pi-stacking interactions at 3.0; edge-to-face pi-stacking interactions at 1.0; and metallic complexation interactions at 3.0. This is a crude approximation, as the optimal weight of each interaction might vary significantly. Nonetheless, it is suitable as a proof-of-concept and can be replaced with a more precise metric. We score each of the 1,000 selected complexes using this method, thereby determining a score for each molecule to serve as a proxy for its potential to bind the protein target.

### 2.8 Curating the Active Learning Training Set and Fine-Tuning the Model

#### Creating the Active Learning Training Set:

After scoring each of the 1,000 protein-ligand pairs, we select *N* replicas of each molecule that scores equal to or above a defined threshold of 11, where *N* is the smallest integer that achieves a total number of molecules of at least 5,000. We then map all scores back to the original clusters and calculate mean cluster scores, which are converted to sampling fractions with the softmax function. We also consider other methods for converting cluster scores to sampling fractions and report the results in the Supporting Information (Figures S3.1–S6.5). We sample *f*_*i*_ × 5,000 molecules randomly from each cluster to obtain a total of 5,000 molecules, where *f*_*i*_ is the calculated fraction for sampling from cluster *i*. If a given cluster has fewer molecules than would satisfy the calculated fraction, we distribute the surplus among the other clusters relative to their sampling fractions. We combine these 5,000 molecules with the replicas of molecules that met the scoring threshold to generate a training set for AL of at least 10,000 molecules. This protocol allows us to beneficially incorporate molecules that have not been scored while retaining emphasis on those that have obtained high scores.

#### Performing the Active Learning Training:

After compiling the training set to be used for AL, the model is further trained for 10 epochs using a learning rate of 3×10^−5^, with no warmup and a cosine decay to 3×10^−6^.

The fine-tuned model is then used to generate 100,000 unique molecules which are subsequently used for another iteration of the pipeline. This iterative approach allows us to locate regions of chemical space corresponding to high-scoring molecules. The process is continued until a satisfactory alignment of the model is achieved.

## 3. Results and Discussion

### 3.1 Validating Our Pretrained GPT Model

**Figure 2.**
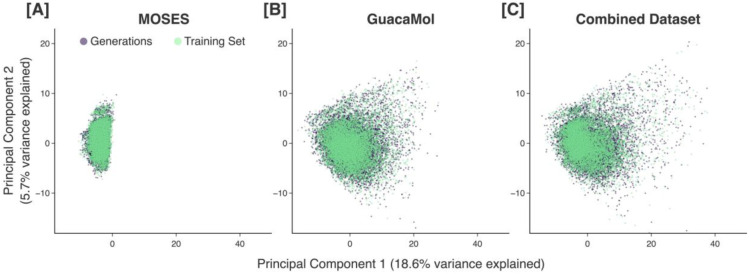
Different pretraining sets (green) plotted with the molecules generated by the corresponding pretrained model (purple). Data points are projected onto the first two principal components of our chemical space proxy. Results are shown for the MOSES (A), GuacaMol (B), and combined (C) pretraining sets.

Before presenting the capabilities of our proposed methodology, it is necessary first to demonstrate that our pretrained GPT model is able to generate molecules that are representative of the chemical space spanned by the training set. Visualizing each pretraining set that we use and the corresponding generated molecules projected into our chemical space proxy, we see that our pretrained models are able to generate molecules that sufficiently cover the area spanned by the corresponding pretraining set (Figure 2).

To further substantiate our pretrained GPT model, we show that it is competitive with top-performing models in the field at generating a broad distribution of molecules. Many generative AI models for molecular discovery have been evaluated with the MOSES benchmark.^91^ This benchmark constitutes an important standard for the field, with the objective of assessing models’ abilities to generate diverse collections of novel and valid molecules. There are other metrics used in the MOSES benchmark that assess how closely the set of generated molecules resembles the training set, but for more targeted tasks such as protein-binder design, this objective may not be desirable, as the optimal molecules may reside in a small region of chemical space which is not well-represented in the training data. However, it is necessary for our pretrained model to initially generate a wide range of molecules, as this allows our pipeline to begin with a diverse and broad representation of chemical space that can then be refined through AL. For this reason, we evaluate our pretrained model on the complete MOSES benchmark and show that our model performs among the best in the field (Supporting Information, Figures S7.1–S7.2), establishing its merit as a starting point for AL. Although we utilize a GPT-based model in this work, we suspect that the model can be successfully substituted as more capable architectures are developed, while still retaining the core advantages of our novel AL methodology.

### 3.2 Substantiating Our Scoring Function and Selection Criterion

**Figure 3.**
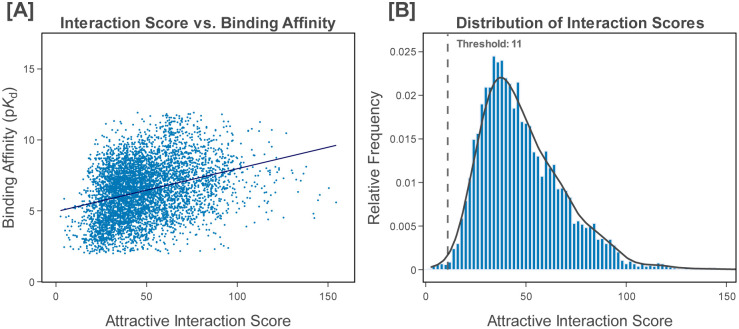
Evaluation of our attractive interaction-based scoring function with the protein-ligand complexes in the PDBbind v.2020 refined set. (A) Binding affinity (p*K*_d_) plotted as a function of attractive interaction score. There is a corresponding Pearson correlation of 0.32. (B) The relative frequency of different scores. 99.6% of complexes exceed our score threshold of 11.

We assess the validity of our scoring function with the PDBbind v2020 refined set, which contains 5,316 unique experimentally determined protein-ligand binding complexes with high-quality labels and structures.^97^ RDKit fails to interpret 21 of the complexes, and another 112 complexes contain metal atoms that prolif is not able to process, resulting in a dataset of 5183 complexes. We find that there is a positive Pearson correlation of 0.32 between the scores derived from our heuristic function and the experimentally determined binding affinities (Figure 3A), supporting our scoring function as an approximate yet meaningful proxy for binding ability. Furthermore, we find that 99.6% of the complexes score at least 11, substantiating our choice of threshold value (Figure 3B).

### 3.3 Performing Naïve Active Learning to Establish a Baseline

**Figure 4.**
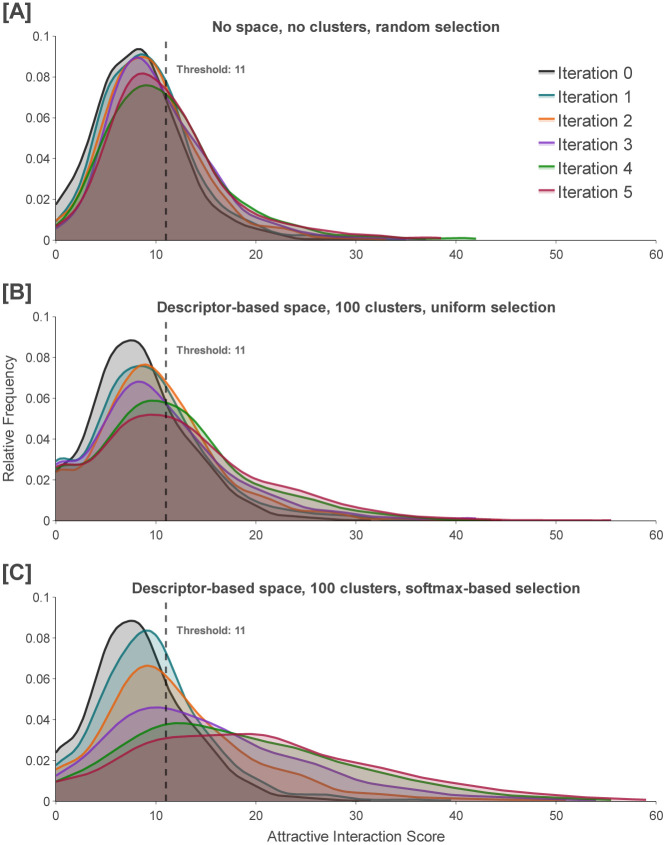
Attractive interaction scores of scored molecules across five iterations of active learning. Results for the naïve sampling method are shown in (A), with random selection of molecules and active learning with only those that score equal to or above the score threshold of 11. Results involving only the diffusion effect are shown in (B), with cluster-based sampling where each cluster is assigned a sampling fraction *f* = 0.01 to generate the active learning set. Results for our proposed active learning methodology are shown in (C). Iteration 0 refers to the pretraining phase, while later iterations refer to the active learning phases.

In order to establish a control, we perform a naïve version of AL where we generate 100,000 unique molecules, randomly select 1,000 of them, and then dock and score each of the selected molecules. We construct the AL training set from *N* replicas of each molecule that scores equal to or above our score threshold, where *N* is the smallest integer that achieves a total number of molecules of at least 5,000. We repeat this procedure for five iterations, and observe that the percentage of generated molecules above the score threshold increases from 26.2% to 44.2% (Figure 4A).

### 3.4 Validating Our Chemical Space

**Figure 5.**
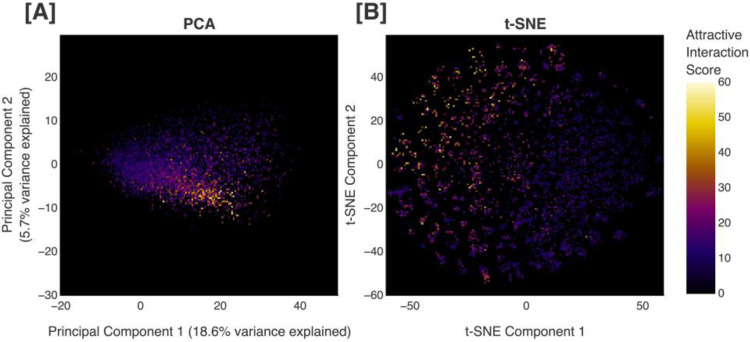
Visualization of scored molecules in lower-dimensional spaces. (A) Generated molecules projected along the first two principal components (18.6% and 5.7% of total variance explained, respectively) of our chemical space proxy, and (B) two-dimensional t-distributed stochastic neighbor embedding (t-SNE) plot of the generated molecules. Plots are colored by score obtained with our heuristic attractive interaction-based scoring function, where black/purple corresponds to lower scores and white/yellow corresponds to higher scores.

To improve upon naïve AL, we utilize a clustering method to group molecules with similar properties. In order to achieve this goal, we construct a representation of chemical space that is based on specific molecular characteristics. To locate regions with high scores, a correlation must exist between position in this space and values produced by our scoring function. Visualizing all of the scored molecules from each iteration of our pipeline (6,000 molecules) along the first two principal components of our chemical space proxy, we are able to observe a continuous gradient of scores (Figure 5A), demonstrating the relation between position in our chemical space proxy and values produced by our objective function.

Moreover, we visualize the scored molecules using t-distributed stochastic neighbor embedding (t-SNE),^98^ a dimensionality reduction technique that can capture nonlinear structures often missed by linear methods like PCA. It is worth noting that in this work, we perform t-SNE (perplexity=40, early exaggeration=60) only once to ensure a constant coordinate system for comparison across different AL iterations (full method in Supporting Information, Section 8). When the scored molecules are plotted using t-SNE, we see that the regions containing molecules with higher scores are easily identifiable (Figure 5B). These results, in combination with those obtained using PCA, confirm the connection between our approximation to chemical space and our attractive interaction-based scoring function.

### 3.5 Assessing the Diffusion Effect

Because the generated molecules are not evenly distributed in the constructed chemical space, cluster-based sampling introduces a bias in which molecules from less dense regions are sampled more frequently than they are with random selection, leading to a score-independent shift in the distribution throughout AL iterations. To assess this bias, which we refer to as the *diffusion effect*, we construct AL training sets containing at least 10,000 molecules by selecting molecules with scores equal to or above the defined score threshold (at least 5,000 molecules including replicas) and sampling from each cluster with the same sampling fraction *f* = 0.01 (5,000 molecules). Notably, the magnitude of the shift in the score distribution is slightly larger than that achieved via random selection (Figure 4B). Specifically, the percentage of generated molecules equal to or greater than our score threshold increases from 28.1% to 51.1% after five iterations of AL.

### 3.6 Evaluating the Capabilities of the ChemSpaceAL Methodology

In contrast to our protocol for assessing the diffusion effect, our complete pipeline involves sampling from clusters according to fractions obtained by applying the softmax function to the mean cluster scores. We observe that the percentage of generated molecules with scores equal to or greater than the score threshold is increased from 28.1% to 76.0% after five iterations of AL, nearly a three-fold enhancement (Figure 4C). More detailed metrics regarding the continuous improvement of the distribution of generated molecules are shown in Table 1.

After each AL iteration, the percentage of generated molecules with scores above the score threshold, the mean score, the scores at the first, second, and third quartiles, as well as the maximum score all increase, indicating that the broad population of generated molecules is enhanced throughout our methodology. Since 99.6% of protein-ligand complexes in the PDBbind v2020 refined set exhibit scores equal to or above the score threshold (Figure 3B), this threshold empirically constitutes a minimal requirement for binding. Therefore, almost 72% of the molecules initially generated by the pretrained model have a near-zero probability of binding, while this metric plummets to 24% after employing our methodology, indicating a vast improvement of the resulting library.

### 3.7 Further Analyzing Our Sampling Approach

**Figure 6.**
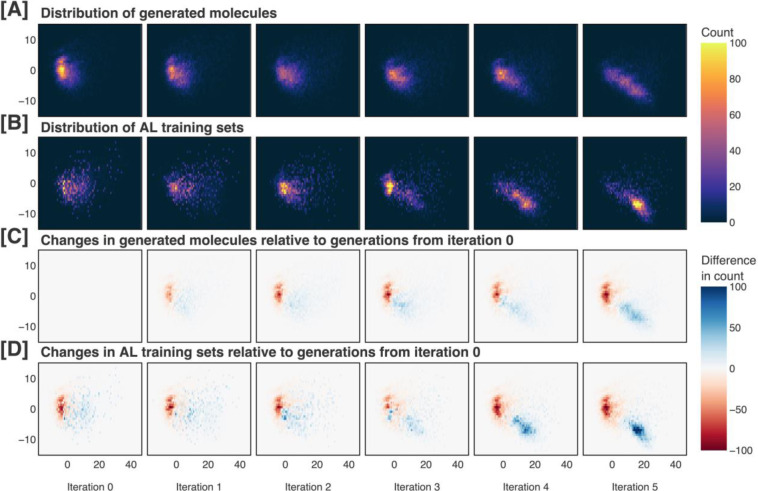
Generated molecules and active learning training sets across each iteration of our pipeline, visualized along the first two principal components of our chemical space proxy. The generated molecules and active learning training sets are shown in (A) and (B), respectively. Changes in the generated molecules and active learning training sets relative to the molecules generated at iteration 0 are shown in (C) and (D), respectively. Iteration 0 refers to the pretraining phase, while later iterations refer to the active learning phases.

**Figure 7.**
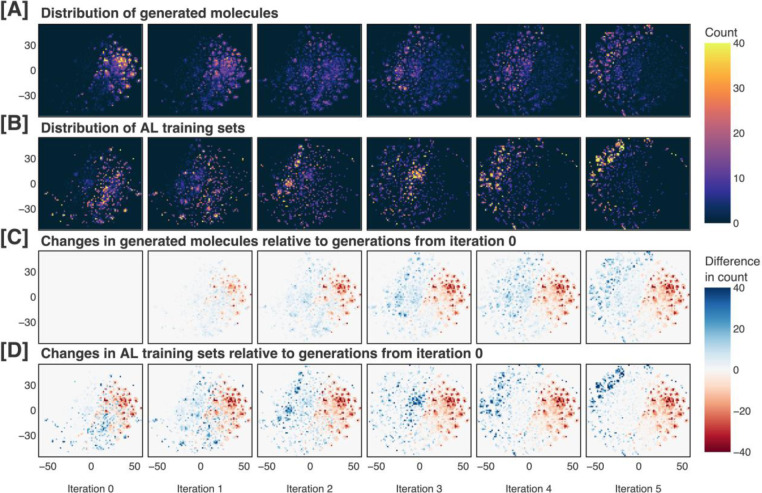
Generated molecules and active learning training sets across each iteration of our pipeline, visualized in two dimensions after performing t-distributed stochastic neighbor embedding (t-SNE). The generated molecules and active learning training sets are shown in (A) and (B), respectively. Changes in the generated molecules and active learning training sets relative to the molecules generated at iteration 0 are shown in (C) and (D), respectively. Iteration 0 refers to the pretraining phase, while later iterations refer to the active learning phases.

If our sampling approach were insufficient at differentiating between distinct regions within the generated distribution, we would expect to create AL training sets that were highly similar to the generated distribution at the same iteration. Figure 6A–B and Figure 7A–B demonstrate that this is not the case, showing that certain subregions are amplified or suppressed at each iteration. Furthermore, in Figure 6C–D and Figure 7C–D, it is clear that both the generated and AL sets evolve significantly relative to the generations from iteration 0. Visualizing the evolution of the generated and AL sets, we see that our methodology shifts the distributions continuously and in a constant direction.

## 4. Summary

We have developed a novel AL methodology that can efficiently improve the outputs of a generative model with respect to an objective function by strategically operating within a constructed sample space. We demonstrate the capabilities of this methodology in the context of targeted molecular generation by fine-tuning a GPT model to produce molecules that exhibit higher attractive interaction scores with the HNH domain of Cas9. In contrast to previous AL methods for molecular generation, our approach does not require scoring each data point that is included in the AL training set, allowing for the incorporation of more computationally expensive metrics such as those requiring protein-ligand docking poses.

## 5. Future Outlook

We envision that the interchangeability of the generative model, constructed sample space, and scoring function renders our methodology adaptable to future innovations. For instance, the GPT model could be replaced by a more capable architecture as soon as one is developed. In addition, rather than constructing a sample space from molecular descriptors, any quantifiable features that are related to the defined objective function can be used. In the context of molecular generation, the list of descriptors used to construct our chemical space proxy could be substituted as better molecular descriptors are developed. Moreover, the scoring function that we use can be replaced by a more computationally expensive metric such as a molecular mechanics-based scoring function to achieve higher precision. The efficiency and generality of our approach facilitate the applicability and utility of the ChemSpaceAL methodology both at present and as the state of the field inevitably improves.

## Supplementary Material

1

## Figures and Tables

**Figure 1.**
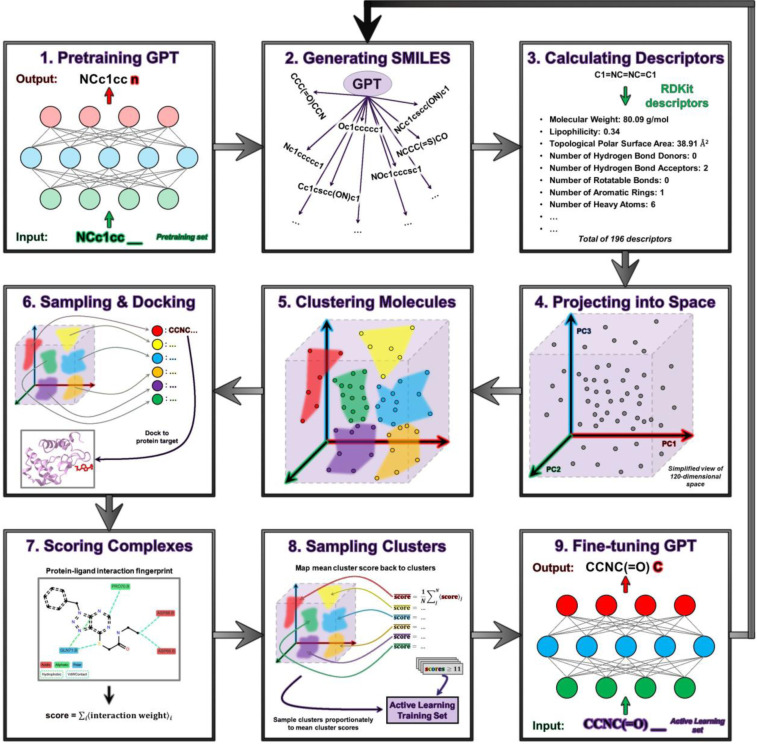
Process flow diagram depicting the complete pipeline of the ChemSpaceAL active learning methodology applied to molecular generation.

**Table 1.** Statistics regarding the distribution of generated molecules across our complete active learning pipeline.

Iteration	Percent > 11	Q1	Q2	Mean	Q3	Max
0	28.1%	5.5	8.0	8.0	11.5	31.5
1	37.0%	6.0	9.0	9.8	12.5	39.5
2	49.7%	7.5	10.5	12.2	16.0	51.0
3	62.6%	8.0	13.5	15.1	20.6	54.0
4	72.9%	10.0	16.5	18.2	25.0	55.5
5	76.0%	11.0	19.0	20.1	27.6	59.0

a The percentage of generated molecules with attractive interaction scores equal to or above our score threshold is shown (Percent > 11), as well as the score at the first quartile (Q1), second quartile (Q2), mean, third quartile (Q3), and maximum of the distribution.

b Iteration 0 refers to the pretraining phase, while later iterations refer to the active learning phases.

## Data Availability

All of our software is available as open source at https://github.com/gregory-kyro/ChemSpaceAL/. Additionally, the ChemSpaceAL Python package is available via PyPI at https://pypi.org/project/ChemSpaceAL/.

## Abbreviations

AI: artificial intelligence
GPT: generative pretrained transformer
RNNs: recurrent neural networks
GANs: generative adversarial networks
AEs: autoencoders
SMILES: simplified molecular-input line-entry system
ML: machine learning
RL: reinforcement learning
AL: active learning
QED: quantitative estimate of drug-likeness
NaN: not a number
PCA: principal component analysis
t-SNE: t-distributed stochastic neighbor embedding.
