# ChemSpaceAL: An Efficient Active Learning Methodology Applied to Protein-Specific Molecular Generation

**Published:** 2023-12-04

**Authors:** Gregory W. Kyro, Anton Morgunov, Rafael I. Brent, Victor S. Batista

**Affiliations:** Yale University

## Abstract

The incredible capabilities of generative artificial intelligence models have inevitably led to their application in the domain of drug discovery. Within this domain, the vastness of chemical space motivates the development of more efficient methods for identifying regions with molecules that exhibit desired characteristics. In this work, we present a computationally efficient active learning methodology that requires evaluation of only a subset of the generated data in the constructed sample space to successfully align a generative model with respect to a specified objective. We demonstrate the applicability of this methodology to targeted molecular generation by fine-tuning a GPT-based molecular generator toward a protein with FDA-approved small-molecule inhibitors, c-Abl kinase. Remarkably, the model learns to generate molecules similar to the inhibitors without prior knowledge of their existence, and even reproduces two of them exactly. We also show that the methodology is effective for a protein without any commercially available small-molecule inhibitors, the HNH domain of the CRISPR-associated protein 9 (Cas9) enzyme. We believe that the inherent generality of this method ensures that it will remain applicable as the exciting field of in silico molecular generation evolves. To facilitate implementation and reproducibility, we have made all of our software available through the open-source ChemSpaceAL Python package.

## 1. Introduction

The vast majority of pharmaceutical drugs function by targeting a specific protein.^1^ Virtual screening and de novo drug design are popular methods for developing effective drugs.^2^ Molecular generation methods powered by generative artificial intelligence (AI) can benefit both of these strategies, and there have been numerous reports of recurrent neural networks (RNNs),^3–25^ generative adversarial networks (GANs),^26–39^ autoencoders,^40–63^ and transformers^64–71^ demonstrating remarkable capabilities.

Active Learning (AL) methods can be used to fine-tune an AI model with selectively chosen data points, ensuring that the model retains its broad domain knowledge while narrowing its focus toward a more precise objective. In its basic form, AL can be applied by exclusively using data points that have been directly evaluated and satisfy specific criteria. However, within the AL framework, it is feasible to extend traditional methods by not only including directly evaluated data points, but also incorporating a mechanism that utilizes unevaluated data points similar to the evaluated ones deemed satisfactory. This approach facilitates the use of resource-intensive scoring functions that otherwise would be too expensive by scoring only a strategically selected subset of data points and extending the insights obtained from the scores to data that have not been evaluated. In this context, the total computational cost is largely dependent on the number of sampled molecules necessary to sufficiently represent the ideal search space.

Although there are many notable examples of AL methods for supervised learning tasks related to virtual screening for drug discovery,^72–77^ the application of AL in generative AI for molecular generation is comparatively unexplored. In this emerging field, Filella-Merce et al. recently presented a two-tiered AL strategy using a variational autoencoder, where an inner loop filters molecules based on molecular properties, and an outer loop docks the molecules that pass the inner loop’s filters to a protein target to identify molecules with satisfactory in silico binding affinities.^52^ While this method is successful in generating chemically viable molecules with improved predicted affinity toward the target, it relies on docking each molecule in the outer loop, which is computationally expensive. It is therefore of interest to develop a computationally efficient approach for fine-tuning a molecular generator toward a protein target that does not require docking each molecule, and instead exploits a strategic method for estimating binding ability of molecules that have not been directly evaluated. This would significantly enhance the computational efficiency of AL for aligning molecular generators toward specified targets.

In this work, we present an efficient AL methodology that employs a strategic sampling algorithm and requires evaluation of only a subset of the generated data to successfully align the generated molecular ensemble toward a specified protein target. Specifically, we demonstrate the effectiveness of our methodology by independently aligning a Generative Pretrained Transformer (GPT)-based model to c-Abl kinase and the HNH domain of the CRISPR-associated protein 9 (Cas9) enzyme.^78,79^

## 2. Overview of the ChemSpaceAL Methodology

**Figure 1.**
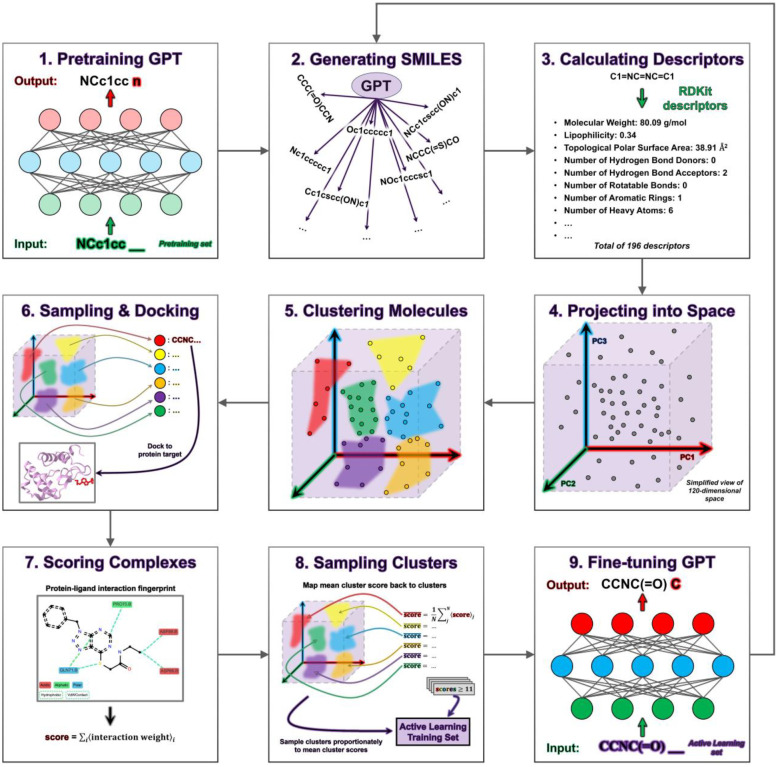
Process flow diagram depicting the complete ChemSpaceAL active learning methodology applied to molecular generation.

Our demonstration of the ChemSpaceAL methodology applied to molecular generation (Figure 1) proceeds as follows:

1. Pretrain the GPT-based model on millions of SMILES (Simplified Molecular Input Line Entry System) strings
2. Use the trained model to generate 100,000 unique molecules (determined by SMILES-string canonicalization)
3. Calculate molecular descriptors for each generated molecule
4. Project the descriptor vectors of the generated molecules into a Principal Component Analysis (PCA)-reduced space constructed from the descriptors of all molecules in the pretraining set
5. Use k-means clustering on the generated molecules within the space to group those with similar properties
6. Sample about 1% of molecules from each cluster and dock each of them to a protein target (e.g., c-Abl kinase or the HNH domain of Cas9)
7. Evaluate the top-ranked pose of each protein-ligand complex with an attractive interaction-based scoring function
8. Construct an AL training set by sampling from the clusters proportionally to the mean scores of the evaluated molecules within each respective cluster, and combining the sampled molecules with replicas of the evaluated molecules whose scores meet a specified threshold
9. Fine-tune the model with the AL training set

*) Repeat steps (2) – (9) for multiple iterations

## 3. Aligning the Generative Model to Specified Protein Targets

Utilizing a transformer decoder-based GPT model (more details in Section 7),^80^ our initial goal is to pretrain the model on data that span as much of true chemical space as possible. This approach allows the pretrained model to develop a rich internal representation of SMILES strings, enabling it to generate a diverse array of molecules. To curate an extensive dataset for pretraining the model, we combine SMILES strings from four datasets: ChEMBL 33 (about 2.4 million bioactive molecules with drug-like properties),^81^ GuacaMol v1 (about 1.6 million molecules derived from ChEMBL 24 that have been synthesized and tested against biological targets),^82^ MOSES (about 1.8 million molecules selected from Zinc 15 to maximize internal diversity and suitability for medicinal chemistry),^83,84^ and BindingDB 08–2023 (about 1.2 million unique small molecules bound to proteins).^85^ After processing, the resulting dataset contains about 5.6 million unique and valid SMILES strings, and will be referred to as the *combined dataset*. More details regarding the data that we use and the preprocessing methods that we employ are discussed in Section 6. To assess the dependence of our methodology on the nature of the pretraining set, we compare two independent models: one pretrained on the combined dataset (C model), and one pretrained on the MOSES dataset (M model).

**Figure 2.**
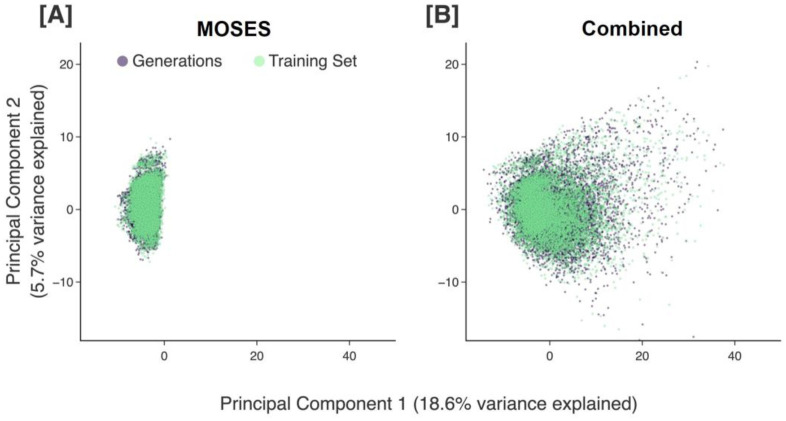
Different pretraining sets (green) plotted with the molecules generated (purple) by the corresponding pretrained model that is trained only on the respective pretraining set. 100,000 data points are randomly sampled from each pretraining set, and 100,000 are generated in each case. The descriptor vectors of the data points are projected into our chemical space proxy and the first two principal components are shown. Results are displayed for the MOSES **(A)** and combined **(B)** pretraining sets.

In Figure 2, we show 100,000 generated molecules from each model trained solely on either the MOSES or combined dataset along the first two principal components of our chemical space proxy. It should be noted that the PCA reduction is performed only once on the molecular descriptors of all molecules in the combined dataset and the obtained principal components are used for all visualizations throughout this work, ensuring fair comparison between different sets of data points (more details in Section 8.1). We see that the pretrained models are able to generate molecules that roughly cover the area spanned by the corresponding pretraining sets (Figure 2).

Using both pretrained models, we independently assess the ChemSpaceAL methodology with c-Abl kinase and the HNH domain of Cas9. In the first case, we aim to validate our methodology by showing that the generated molecular ensemble evolves toward the FDA-approved small-molecule inhibitors of c-Abl kinase. In the latter case, we investigate the applicability of the methodology to a protein without any commercially available small-molecule inhibitors.

In both cases, the generated molecules are filtered based on ADMET (Absorption, Distribution, Metabolism, Excretion, and Toxicity) metrics and functional group restrictions.^86^ ADMET filters are employed to ensure that the molecules possess drug-like properties, and functional group restrictions are used to discard chemical moieties that are less favorable for biological applications. More details regarding the ADMET and functional group filters that we use are reported in Tables S1.1 and S1.2 in the Supporting Information.

### 3.1. Aligning to C-Abl Kinase

C-Abl kinase (PDB ID: 1IEP)^72^ is of significant scientific interest because its dysfunction is associated with the development of chronic myeloid leukemia, making it a vital target for anticancer drugs designed to inhibit its activity and thereby control the proliferation of cancer cells. There are multiple FDA-approved small-molecule inhibitors of c-Abl kinase that have similar structures, including imatinib, nilotinib, dasatinib, bosutinib, ponatinib, bafetinib, and asciminib.^78,87,88^ We dock and score each of the inhibitors using our scoring function, and choose the lowest score among them (37) to be the score threshold for our methodology (more details in Section 8.3).

**Figure 3.**
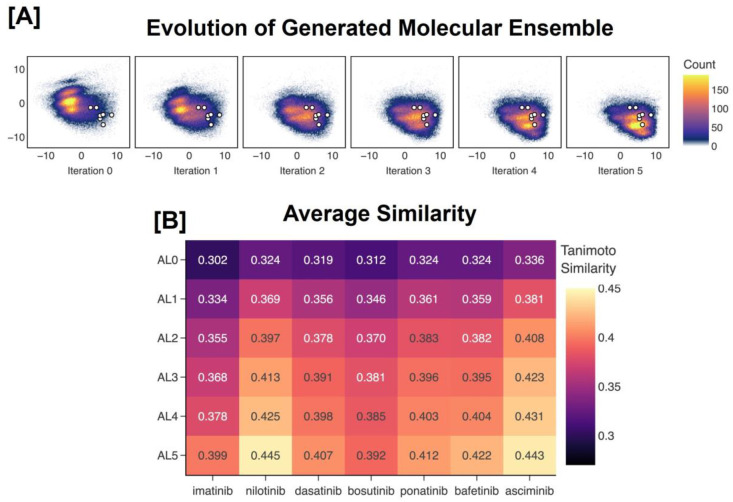
Comparing the evolution of the generated molecular ensemble from the model pretrained on the combined dataset to the FDA-approved small-molecule inhibitors of c-Abl kinase. In **(A)**, the descriptor vectors of the generated molecules across each iteration of our methodology are projected into our chemical space proxy and visualized along the first two principal components. The inhibitor descriptor vectors are also projected into the space and are represented by white dots with black outline. In **(B)**, the average Tanimoto similarities between the RDKit fingerprints of all generated molecules at each iteration and that of each inhibitor are shown. Tanimoto similarities between the inhibitors are reported in Figure S2.1 of the Supporting Information. Iteration 0 refers to the pretraining phase, while later iterations refer to the active learning phases.

**Figure 4.**
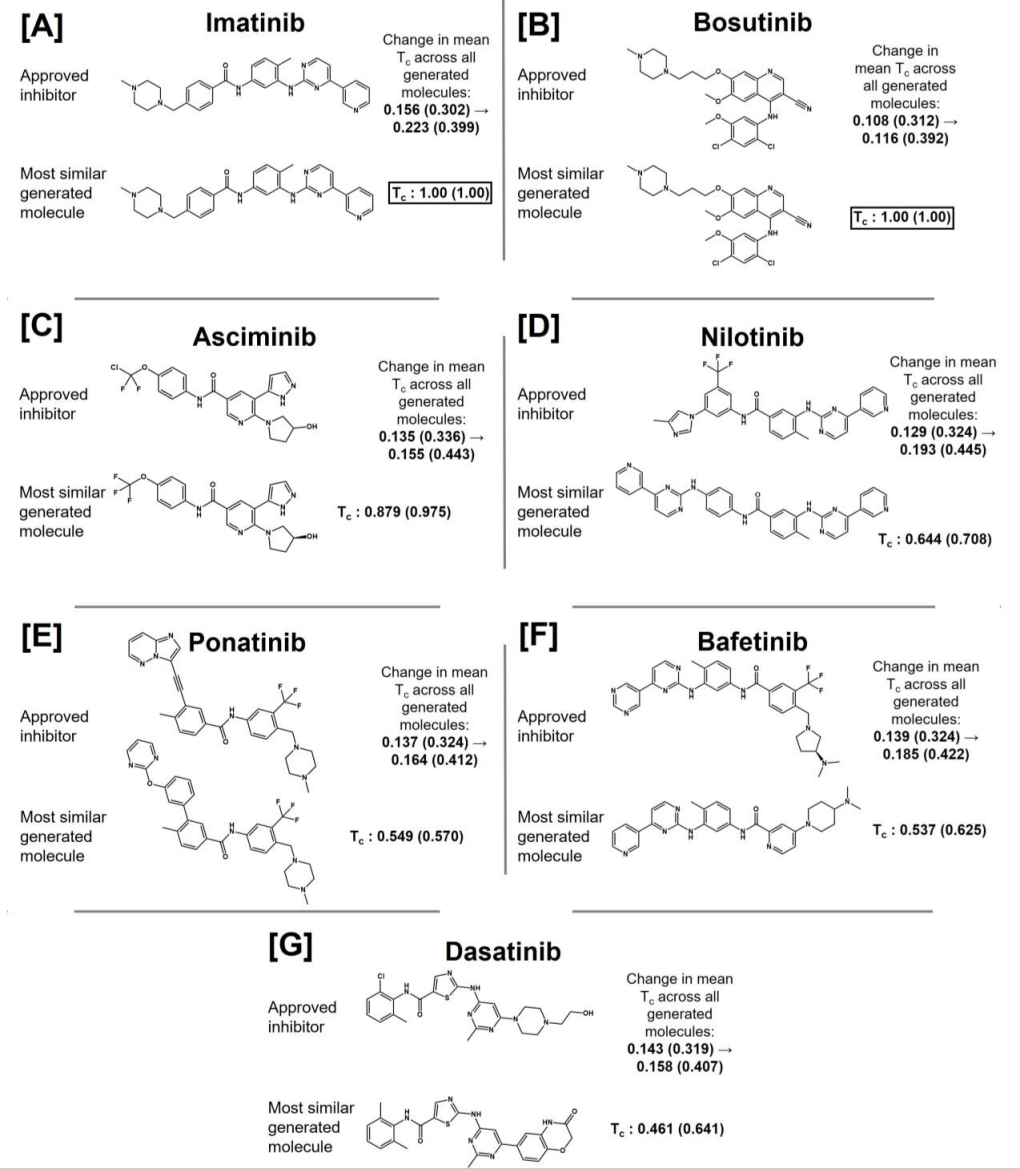
Comparison of the generated molecular ensemble from the model pretrained on the combined dataset to the FDA-approved small-molecule inhibitors of c-Abl kinase. For each inhibitor, the most similar generated molecule, after five iterations, is shown, as well as the Tanimoto similarity (T_C_) between the two. The change in the mean similarity between each inhibitor and all generated molecules from iteration 0 (pretrained model) to iteration 5 is shown. For all comparisons in this figure, the T_C_ between Extended-Connectivity Fingerprint 4s is shown along with the T_C_ between RDKit fingerprints in parentheses.^89,90^ Results are shown for imatinib **(A)**, bosutinib **(B)**, asciminib **(C)**, nilotinib **(D)**, ponatinib **(E)**, bafetinib **(F)**, and dasatinib **(G)**.

For the C model, the mean Tanimoto similarities between the generated molecular ensemble and each of the seven inhibitors increase at each iteration, indicating a constant evolution toward the inhibitors (Figure 3B). This shift of the distribution toward the region of space that contains the FDA-approved inhibitors can be visualized by projecting the descriptor vectors of the generated ensemble at each iteration of the methodology and those of the inhibitors into the chemical space proxy (Figure 3A). Moreover, the set of generated molecules after five iterations contains imatinib and bosutinib (Figure 4).

**Figure 5.**
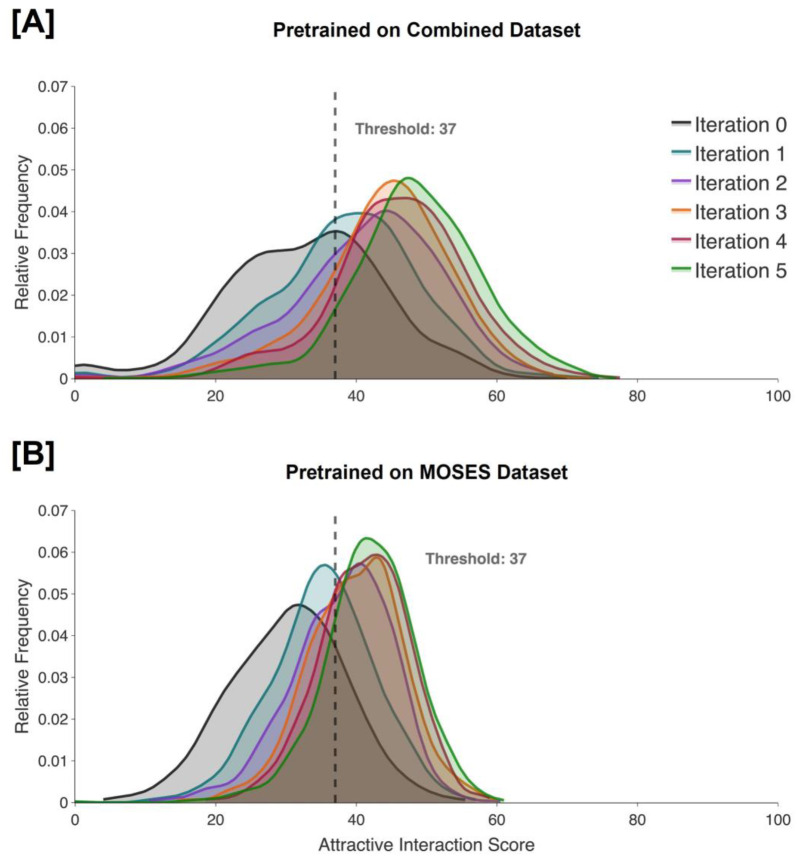
Attractive interaction scores of evaluated molecules across five iterations of active learning for c-Abl kinase. The distributions for the model pretrained on the combined dataset are shown in **(A)**, and the distributions for the model pretrained on the MOSES dataset are shown in **(B)**. Iteration 0 refers to the pretraining phase, while later iterations refer to the active learning phases.

We also assess the performance of the methodology by analyzing the distribution of scores of generated molecules throughout AL iterations. For both the C and M models, the percentage of molecules that reaches the scoring threshold is significantly increased after five iterations of AL, further validating the applicability of our method to c-Abl kinase; the percentage is increased from 38.8% to 91.6% for the C model, and from 21.7% to 80.3% for the M model (Table 1). The evolutions of these distributions can be seen in Figure 5.

It is worth nothing that 38.8% of the molecules generated by the C model reach the score threshold immediately after pretraining, while only 21.7% of the molecules generated by the M model reach the threshold, indicating that our combined pretraining set covers regions of chemical space not spanned by the MOSES dataset that contain higher-scoring molecules (Table 1). Moreover, after applying the methodology, the molecular ensemble generated by the C model is more similar to the FDA-approved inhibitors than that generated by the M model (Figure S3.1 in the Supporting Information), and is comprised exclusively of molecules with satisfactory ADMET profiles (Figure S4.1 in the Supporting Information). These results support the notion that our methodology is more effective at generating drug-like molecules specific to a protein target by pretraining on the combined dataset and applying filters to the generation stage, rather than pretraining on a refined dataset such as the MOSES dataset.

### 3.2. Aligning to the HNH Domain of Cas9

To further evaluate our methodology, we apply it to a protein without any commercially available small-molecule inhibitors, the HNH domain of Cas9 (PDB ID: 6O56).^79^ This protein is a nuclease component critical to the function of the CRISPR/Cas9 system, and is responsible for cleaving the target DNA strand complementary to the guide RNA, which directs the Cas9 enzyme to the correct sequence for gene modification. The HNH domain of Cas9 is therefore particularly interesting because understanding its structure and dynamics can lead to enhancements in the precision and efficiency of CRISPR-based gene editing tools.^91^ Furthermore, the ability to develop binders for HNH could offer a direct way to modulate its behavior.

Our methodology requires a score threshold in order to select molecules to be included in the AL training set. In the absence of known small-molecule binders for HNH, we refer to a large database of experimentally determined protein-ligand complexes, the PDBbind v2020 refined set,^92^ and select this threshold to be 11 (more details in Section 8.3). This lack of known binders also leads us to use the change in the distribution of scores as the primary metric for evaluation. After five iterations of AL, the percentage of generated molecules that reaches the score threshold increases from 21.3% to 52.1% for the C model, and from 14.3% to 28.2% for the M model (Table 2); the performance differential between the C and M models is commensurate with that observed for c-Abl kinase. The evolutions of these distributions can be seen in Figure S5.1 in the Supporting Information.

## 4. Evaluating Individual Components of the Methodology

The goal of this section is to isolate and analyze the effectiveness of individual components of our methodology: the chemical space proxy, clustering algorithm, scoring method, and sampling algorithm for constructing AL training sets. For all results presented here, the methodology is applied to the model pretrained on our combined dataset, aligned to HNH, and without any filters during generation stages. It is imperative to note that the function of the proposed methodology is simply to align the model toward achieving high scores as determined by a scoring function, through a constructed space that correlates with this function. In this section, we therefore seek to evaluate how the model responds with respect to the scoring function, and do not consider any filters on the generated molecules. However, to ensure a rigorous assessment, we additionally perform analogous analyses of the methodology applied to c-Abl kinase with ADMET and functional group filters applied to the generated molecules, and observe similar results as those included in this section (Figures S3.2 and S3.4 in the Supporting Information).

### 4.1. Naïve Active Learning Control

**Figure 6.**
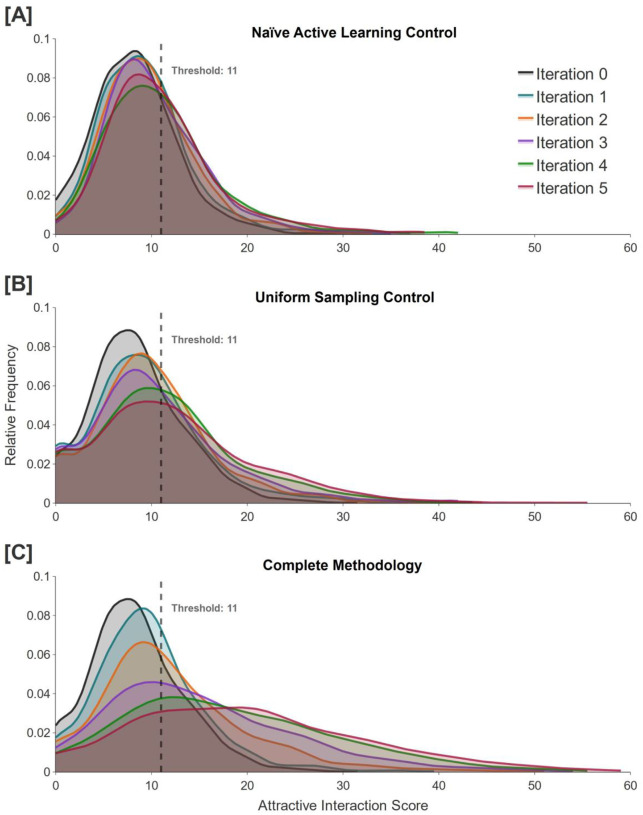
Attractive interaction scores of evaluated molecules across five iterations of active learning. Results for the naïve active learning control are shown in **(A)**, which utilizes random selection of molecules and fine-tuning with only replicas of those that score equal to or above the score threshold of 11. Results for the uniform sampling control are shown in **(B)**, which uses cluster-based sampling where each cluster is assigned a sampling fraction *f* = 0.01 during the construction of the active learning set. Results for our complete methodology are shown in **(C)**. Iteration 0 refers to the pretraining phase, while later iterations refer to the active learning phases.

In order to establish a baseline for comparison to our methodology, we perform a naïve version of AL where we generate 100,000 unique molecules, randomly select 1,000 of them, dock and score each of the selected molecules, and then fine-tune the model with the scored molecules that reach the score threshold. The purpose of this approach is to demonstrate how the fine-tuning would occur if we did not sample from clusters in the chemical space proxy to construct an AL training set. In this case, we construct the AL training set from *N* replicas of each molecule that scores equal to or above the score threshold (11), where *N* is the smallest integer that achieves a total number of molecules of at least 5,000. The model is then further trained on this AL set, and the fine-tuned model is used to generate another 100,000 unique molecules which are subsequently used for another iteration of the methodology. We repeat this procedure for a total of five iterations, and observe that the percentage of generated molecules that reaches the score threshold increases from 26.2% to 44.2% (Figure 6A).

### 4.2. Chemical Space Proxy and Clustering Algorithm

In order to improve upon naïve AL, we propose to strategically select molecules to be in the AL training set that have not been evaluated. This requires a method for relating molecules that have been scored to those that have not. To achieve this goal, we construct a proxy for chemical space that is predicated on molecular properties, allowing us to operate within a space where nearby molecules share similar chemical features. More details regarding the construction of our chemical space proxy are discussed in Section 8.1.

**Figure 7.**
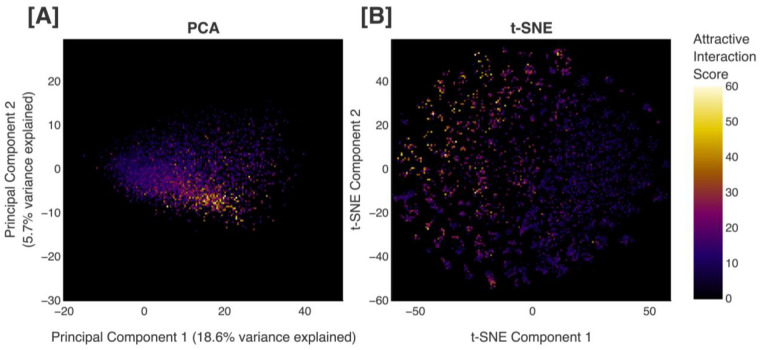
Visualization of scored molecules in the chemical space proxy. All of the scored molecules from all iterations of the complete methodology applied to the model pretrained on our combined dataset, aligned to HNH, and with no filters on the generated molecules (6,000 molecules) are displayed. **(A)** Descriptor vectors of the generated molecules projected into the chemical space proxy and shown along the first two principal components, and **(B)** two-dimensional t-distributed stochastic neighbor embedding (t-SNE) plot of the generated molecules are shown. Plots are colored by score obtained with the scoring function, where black/purple corresponds to lower scores and white/yellow corresponds to higher scores.

A correlation must exist between position in the chemical space proxy and values produced by the scoring function in order to successfully estimate the scores of molecules that have not been evaluated. Visualizing all of the scored molecules from all iterations of the complete methodology (6,000 molecules) along the first two principal components of our chemical space proxy, we observe a continuous gradient of scores (Figure 7A), illustrating the relation between position in our chemical space proxy and values produced by our scoring function. Moreover, when the positions of the scored molecules in the chemical space proxy are reduced to two dimensions using t-distributed stochastic neighbor embedding (t-SNE), a technique that captures nonlinear structures, we also see that the regions containing molecules with higher scores are easily identifiable (Figure 7B; more details in Section 6 of the Supporting Information).

Within our chemical space proxy, we utilize k-means clustering (with k = 100) to group molecules that exhibit similar chemical properties. We also report results for k = 10, which proves to be less effective (see Figures S7.1 – S7.4 in the Supporting Information). This is likely because much of the diversity in the chemical space is homogenized into clusters which, in the case of k = 10, are very large compared to k = 100, and valuable information is lost. In short, we generate 100 clusters and then randomly sample up to 10 molecules from each cluster, selecting all molecules in cases where a cluster contains fewer than 10 molecules. More details of our clustering method are discussed in Section 8.2.

### 4.3. Docking and Scoring

After strategically selecting 1,000 molecules, we dock each of them to a protein target using DiffDock (more details in Section 8 of the Supporting Information),^93^ and evaluate it using our scoring function, which is essentially a sum of attractive protein-ligand contact points, each weighted by its interaction type. More details regarding the scoring function we use are discussed in Section 8.3. We calculate a score for each of the 1,000 docking poses to serve as an estimate for the ligand’s potential to bind the protein target.

### 4.4. Uniform Sampling Control

Because the generated molecules are not evenly distributed in the chemical space proxy, cluster-based sampling introduces a bias in which molecules from less dense regions are sampled more frequently than they would be with random selection. This leads to a score-independent shift in the distribution throughout AL iterations, which we will refer to as the *diffusion effect*. To assess this bias, we construct AL training sets by randomly selecting 10 molecules from each cluster, scoring each of them, selecting the molecules with scores that reach the score threshold (at least 5,000 molecules including replicas), and sampling from each cluster with the same sampling fraction *f* = 0.01 (about 50 from each cluster for a total of 5,000 molecules) for a total of approximately 10,000 molecules. This approach serves as a control for isolating the effectiveness of our algorithm for sampling unscored molecules to be in the AL training set. For this uniform sampling-based approach, the increase in the scores of the molecules in the generated ensemble after five iterations (28.1% to 51.1%) is slightly more pronounced than that achieved via naïve AL (26.2% to 44.2%), as shown in Figure 6B. Although these results mark a slight improvement over those obtained with naïve AL, they are significantly worse than those achieved with our complete methodology (28.1% to 76.0%), indicating that our score-based sampling method is necessary for high performance and aligns the model with the scoring function much more effectively than does uniform sampling.

### 4.5. Sampling from Clusters Proportionally to Their Scores

In order to improve upon uniform sampling, we propose a way to intelligently weight the importance of each cluster when sampling molecules from the chemical space proxy to be in the AL training set. After scoring each of the 1,000 protein-ligand pairs, we sample from the clusters proportionally to the mean scores calculated from the evaluated molecules within each respective cluster. These sampled molecules are then combined with replicas of the evaluated molecules whose scores meet the score threshold, forming the AL training set. More details regarding our sampling algorithm are discussed in Section 8.4. Our sampling procedure allows us to enrich the AL training set with unscored molecules that would likely obtain high scores, exploiting the fact that position in the chemical space proxy correlates with the scoring function (Figure 7).

**Figure 8.**
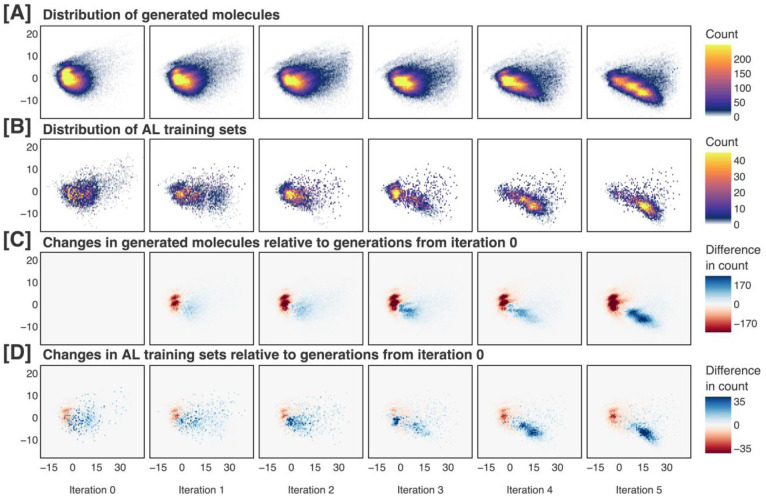
Generated molecules and active learning training sets across five iterations of our complete methodology, visualized along the first two principal components of our chemical space proxy. The generated molecular ensembles and active learning training sets at each iteration are shown in **(A)** and **(B)**, respectively. Changes in the generated molecular ensembles and active learning training sets relative to the molecules generated at iteration 0 are shown in **(C)** and **(D)**, respectively. In **(A)** and **(C)**, the 100,000 unique generated molecules from each iteration are used. In **(B)**, the full active learning training sets, each containing approximately 10,000 molecules, are used. In **(D)**, for proper comparison between the generated molecules at iteration 0 and the active learning training sets, 5,000 molecules are randomly sampled from the generated ensemble at iteration 0, and 5,000 molecules are randomly sampled from the active learning training set at each iteration. Iteration 0 refers to the pretraining phase, while later iterations refer to the active learning phases. More details of this analysis are reported in Figure S9.1 in the Supporting Information.

Our complete methodology shifts the percentage of generated molecules that reaches the score threshold from 28.1% to 76.0% (Figure 6C). This increase is attributable to the shift of the generated molecular ensemble toward the region of the chemical space proxy associated with higher scores. Figure 8 illustrates this progression, depicting the evolution of the generated ensemble in a constant direction through the chemical space proxy.

## 5. Summary and Future Outlook

In this work, we present an efficient AL methodology that requires evaluation of only a subset of the generated data to successfully align a generative AI model with respect to a specified objective. We demonstrate its applicability in the context of targeted molecular generation by independently enhancing attractive interactions between the molecules in the generated ensemble and two protein targets, namely, c-Abl kinase and the HNH domain of Cas9. When aligning toward c-Abl kinase, we are able to shift the distribution of generated molecules toward the region of the chemical space proxy corresponding to several FDA-approved inhibitors for this target. We also show that our methodology is effective for a protein without any commercially available small-molecule inhibitors, the HNH domain of Cas9. Moreover, we analyze the effectiveness of individual components of our methodology, and show that the integration of these components in our complete approach aligns the model with the scoring function much more effectively than more naïve AL methods.

The generative model, constructed sample space, and scoring function are all highly substitutable within the framework of our methodology, and we therefore envision that it will be adaptable to future innovations. For instance, the GPT-based model could be replaced by a more capable architecture as soon as one is developed. In addition, rather than constructing a sample space from molecular descriptors, any quantifiable features that are correlated with the scoring function can be used. In the context of molecular generation, the list of descriptors used to construct our chemical space proxy could be substituted as better molecular descriptors are developed (i.e., ones that correlate better with the scoring function). Moreover, the scoring function that we use can be replaced by a better metric to achieve closer correspondence with experimental results. The generality of our approach facilitates the applicability and utility of the ChemSpaceAL methodology both at present and as the state of the field inevitably improves.

## 6. Dataset Collection and Preprocessing

### 6.1. Data Collection

We combine all of the SMILES strings from ChEMBL 33, GuacaMol v1, MOSES, and BindingDB, filter out the strings that are identified as invalid by the RDKit molecular parser, and remove any duplicate strings. The resulting combined dataset contains 5,622,772 unique and valid SMILES strings.

### 6.2. Tokenization

Our combined dataset initially has a vocabulary of 196 unique tokens. We find that 148 tokens are represented in the dataset fewer than 1000 times; to reduce the size of our vocabulary (from 196 to 48), we remove all SMILES strings containing at least one token that appears less than 1000 times in the combined dataset (details in Tables S10.1 and S10.2 in the Supporting Information). Most of the SMILES strings excluded contain rare transition metals or isotopes.

### 6.3. Data Preprocessing

The longest SMILES string in the combined dataset contains 1,503 tokens, while 99.99% of the strings in the dataset have 133 or fewer tokens (details in Figures S11.1 – S11.2 in the Supporting Information). We impose a SMILES string length cutoff of 133, and remove any string from the dataset whose length is greater than this cutoff. All remaining SMILES strings are then extended, if necessary, to the length of the longest SMILES string in the dataset (133) using a padding token “<”, and are augmented with a start token “!” and an end token “~”. The resulting dataset contains 5,539,765 SMILES strings, which are randomly split into training (5,262,776 entries; 95.0%) and validation (276,989 entries; 5.0%) sets for pretraining.

## 7. Details of the Generative Model

We utilize a GPT-based model (details of the model architecture can be found in Section 12 of the Supporting Information). Our model embeds inputs into a 256-dimensional space, and is composed of eight transformer decoder blocks, each of which contains eight attention heads. Dropout with a probability of 10% is applied after each feed-forward network except for the output layer to mitigate overfitting, and gradient clipping with a maximum norm of 1.0 is used in conjunction with layer normalization to stabilize the optimization process and prevent exploding gradients. All weights are initialized according to a Gaussian distribution with a mean of 0 and a standard deviation of 0.02 except for weights involved in layer normalization, which are initialized to 1, and bias parameters, which are initialized to 0. The training process utilizes cross-entropy loss with L2 regularization applied to the linear layers using λ=0.1, and the SophiaG optimizer with β_1_=0.965, β_2_=0.99 and p=0.04.^94^

### 7.1. Pretraining

During pretraining, the learning rate warms up to 3×10^−4^ until the model has been trained on 10% of the total number of tokens in the dataset, then decays to 3×10^−5^ using cosine decay. The model is trained with a batch size of 512 for 30 epochs. Learning curves are reported in Figures S13.1 and S13.2 in the Supporting Information.

### 7.2. Benchmarking

Many generative AI models for molecular discovery have been evaluated with the MOSES benchmark,^77^ which constitutes an important standard for the field, with the objective of assessing models’ abilities to generate diverse collections of novel and valid molecules. We show that our pretrained model performs among the best in the field (details in Table S14.1 and S14.2 in the Supporting Information), establishing its merit as a starting point for AL.

### 7.3. Fine-tuning

After compiling the AL training set, the model is further trained with a batch size of 512 for 10 epochs using a learning rate of 3×10^−5^, with no warmup and a cosine decay to 3×10^−6^.

## 8. Details of the ChemSpaceAL Methodology

### 8.1. Chemical Space Proxy

We first calculate the full set of molecular descriptors available through RDKit’s CalcMolDescriptors function for each molecule in the combined pretraining set, encompassing a wide range of molecular properties including structural, topological, geometrical, electronic and thermodynamic characteristics. Among these 209 descriptors, 13 return NaN (not a number) or infinity for at least one SMILES string in the dataset and consequently are discarded (details in Table S15.1 in the Supporting Information), resulting in 196 descriptors (details in Table S15.2 in the Supporting Information). We use as many RDKit descriptors as possible because this step in the methodology is very fast (see Figure S16.1 in the Supporting Information), enabling us to generate maximally descriptive molecular representations. We also independently investigate the performance of the methodology using only the 42 RDKit molecular quantum numbers (MQNs), which are not included in the CalcMolDescriptors function, and find this representation to yield worse results than those obtained using the PCA-reduced 120-dimensional representation of the 196 descriptors (Figure S17.1 in the Supporting Information). We note that for the proposed methodology to work, the set of descriptors used must satisfy two criteria: 1) position in the chemical space proxy correlates with the scoring function, and 2) nearby molecules in the chemical space proxy have similar scores. It is evident that there could exist many sets of descriptors satisfying these criteria; a thorough investigation into the choice of descriptors is outside the scope of this work. After performing PCA using the 196 RDKit descriptors for all molecules in the combined pretraining set, we find that 99% of the variance is explained by the first 113 principal components (details in Figure S15.3 of the Supporting Information), and use the first 120 principal components throughout the methodology as our chemical space proxy. Our methodology might attain similar results with fewer principal components retained, but this reduction is not necessary since this step is computationally inexpensive.

### 8.2. Clustering Algorithm

Within our chemical space proxy, we utilize k-means clustering to group molecules that exhibit similar chemical properties, with k = 100. Given that running k-means is incredibly fast, we perform k-means 100 times to mitigate the potential for poor initialization, seeking to minimize k-means loss and cluster size variance. Initially, we take the five clusterings with the lowest loss, thereby preserving those with more compact clusters. Of these five, we select the clustering with the lowest variance in cluster size for use in the following stages of the methodology.

After clustering the generated molecules in our chemical space proxy, we randomly select 10 molecules from each cluster that contains at least 10 molecules, and select all of the molecules from any cluster that contains less than 10 molecules. For AL iterations 1–5, when applying the methodology to the C model for aligning to HNH with no filters on the generated molecules, the number of clusters containing fewer than 10 molecules out of 100 clusters are 4, 3, 5, 2, and 3 for each respective iteration (see Figures S18.1 – S18.4 in the Supporting Information). We then randomly sample from the clusters with more than 10 molecules until we achieve a set of 1,000 molecules.

### 8.3. Scoring Function

Our scoring function considers attractive protein-ligand contact points using the prolif software package,^95^ and assigns handpicked weights for each interaction type: hydrophobic interactions are scored at 2.5; hydrogen-bond interactions at 3.5; ionic interactions at 7.5; interactions between aromatic rings and cations at 2.5; Van der Waals interactions at 1.0; halogen-bond interactions at 3.0; face-to-face pi-stacking interactions at 3.0; edge-to-face pi-stacking interactions at 1.0; and metallic complexation interactions at 3.0.

We assess our scoring function with the PDBbind v2020 refined set, which contains 5,316 unique experimentally determined protein-ligand binding complexes with high-quality labels and structures.^92^ We find that there is a positive Pearson correlation of 0.32 between the scores derived from our scoring function and the experimentally determined binding affinities (Figure S19.1A in the Supporting Information), supporting our scoring function as an approximate yet meaningful estimate of binding ability. Furthermore, we find that 99.6% of the complexes achieve the score threshold of 11 (Figure S19.1B in the Supporting Information).

The optimal weights for the interaction types may vary significantly depending on the specific target, and therefore the scoring function employed in this work should be considered a crude estimation. However, the positive correlation with experimentally determined binding affinities supports its utility as a heuristic approximation to binding ability. Moreover, it can be replaced with a more precise metric, as long as the replacement metric correlates with the descriptors used to construct the chemical space proxy.

### 8.4. Sampling Algorithm

After scoring each of the 1,000 protein-ligand pairs, we select *N* replicas of each molecule that scores equal to or above the score threshold, where *N* is the smallest integer that achieves a total number of molecules of at least 5,000. We then calculate mean cluster scores from the scored molecules, which are converted to sampling fractions with the softmax function. We also consider other methods for converting cluster scores to sampling fractions and report the results for each method attempted (Figures S20.1 – S20.17 in the Supporting Information). We then convert *f*_*i*_ × 5,000 to an integer (where *f*_*i*_ is the calculated fraction for sampling from cluster *i*), and sample the corresponding number of molecules randomly from each respective cluster. If a given cluster has fewer molecules than would satisfy the calculated fraction, we distribute the surplus among the other clusters relative to their sampling fractions. We combine these 5,000 molecules with the replicas of molecules that meet the scoring threshold to generate an AL training set of approximately 10,000 molecules.

## Supplementary Material

1

## Figures and Tables

**Table 1.** Evolution of protein-ligand attractive interaction scores between molecules in the generated ensemble and c-Abl kinase across our complete active learning methodology.

Iter	C %>37	C Mean	C Max	M %>37	M Mean	M Max
0	38.8	32.8	70.0	21.7	30.3	55.5
1	59.3	38.4	74.5	42.1	35.2	57.0
2	70.1	41.4	68.0	59.2	38.0	60.5
3	81.2	44.0	73.5	68.8	39.9	60.0
4	86.6	46.0	77.5	76.2	41.0	59.0
5	91.6	48.5	77.0	80.3	41.8	61.0

a The percentage of generated molecules with attractive interaction scores equal to or above our score threshold (% > 37), the mean score, and the maximum score are shown for the model pretrained on the combined dataset (C), and the model pretrained on the MOSES dataset (M) for five iterations of the methodology.

b Iteration 0 refers to the pretraining phase, while later iterations refer to the active learning phases.

**Table 2.** Evolution of protein-ligand attractive interaction scores between molecules in the generated ensemble and HNH across our complete active learning methodology.

Iter	C %>11	C Mean	C Max	M %>11	M Mean	M Max
0	21.3	7.9	32.5	14.3	7.3	22.5
1	31.9	9.1	26.5	18.9	7.8	21.0
2	39.1	9.8	25.0	22.5	8.2	22.0
3	43.9	10.4	23.0	24.5	8.6	23.0
4	50.1	11.1	33.5	28.7	8.9	21.0
5	52.1	11.5	34.0	28.2	9.0	23.0

a The percentage of generated molecules with attractive interaction scores equal to or above our score threshold (% > 11), the mean score, and the maximum score are shown for the model pretrained on the combined dataset (C), and the model pretrained on the MOSES dataset (M) for five iterations of the methodology.

b Iteration 0 refers to the pretraining phase, while later iterations refer to the active learning phases.

## Data Availability

All of our software is available as open source at https://github.com/batistagroup/ChemSpaceAL. Additionally, the ChemSpaceAL Python package is available via PyPI at https://pypi.org/project/ChemSpaceAL/.

## Abbreviations

AI: artificial intelligence
RNNs: recurrent neural networks
GNNs: generative adversarial networks
AL: active learning
GPT: Generative Pretrained Transformer
Cas9: CRISPR-associated protein 9
SMILES: Simplified Molecular Input Line Entry System
PCA: Principal Component Analysis
ADMET: Absorption, Distribution, Metabolism, Excretion, and Toxicity
T_C_: Tanimoto similarity
LogP: logarithm of the partition coefficient
t-SNE: t-distributed stochastic neighbor embedding
NaN: not a number
MQNs: molecular quantum numbers
