# Supplementary material for: ChemSpaceAL: An Efficient Active Learning Methodology Applied to Protein-Specific Molecular Generation
Source: ArXiv. 2023 Dec 4:arXiv:2309.05853v2. Originally published 2023 Sep 11. Preprint. [Version 2] (PMC10516108)

Supporting Information

ChemSpaceAL: An Efficient Active Learning Methodology Applied to Protein-Specific Molecular Generation

Gregory W. Kyro, Anton Morgunov, Rafael I. Brent, Victor S. Batista

Department of Chemistry, Yale University, New Haven, Connecticut 06511-8499

List of Sections

- **Section 1:** ADMET and Functional Group Filters.
- **Section 2:** Similarity between FDA-Approved Inhibitors of c-Abl Kinase.
- **Section 3:** Comparing the Generations to c-Abl Kinase Inhibitors for Different Methods.
- **Section 4:** Radar Plots Showing Evolution of ADMET Metrics.
- **Section 5:** Scores of Molecules across Five Iterations of Active Learning for HNH.
- **Section 6:** Implementation Details of t-Distributed Stochastic Neighbor Embedding (t-SNE).
- **Section 7:** Choosing the Number of Clusters to Use for k-means.
- **Section 8:** Details and Parameters Used for Running DiffDock.
- **Section 9:** t-SNE Visualization of the Evolution of the Generated Molecular Ensembles.
- **Section 10:** Vocabulary Composition of the Combined Dataset.
- **Section 11:** Frequencies of Block Sizes, Molecular Weights and Tokens in Pretraining Sets.
- **Section 12:** Details of the GPT Architecture.
- **Section 13:** Training the GPT Model.
- **Section 14:** Pretrained GPT Model Performance on the MOSES Benchmark.
- **Section 15:** RDKit Descriptors Used to Construct the Chemical Space Proxy.
- **Section 16:** Wall Times of Each Step in the Complete Pipeline.
- **Section 17:** Evaluating the Methodology with Lower-Dimensional MQN Filters.
- **Section 18:** Frequency as a Function of Cluster Size for Alignment to c-Abl Kinase.
- **Section 19:** Evaluation of Scoring Function Compared to PDBbind v2020 Refined Set.
- **Section 20:** Alternative Methods for Converting Mean Cluster Scores to Sampling Fractions.
- **Section 21:** Distributions of Mean and Median Cluster Scores.
- **Section 22:** Additional Evaluation of Generations across Active Learning Iterations.
- **References**

Section 1: ADMET and Functional Group Filters.

Table S1.1. Upper and lower bounds applied to each ADMET metric used for generation filter. We use upper bound for logP of 6.5 because one of the FDA-approved inhibitors of c-Abl kinase, nilotinib, has a value of 6.356 as calculated by RDKit. All other bounds are taken from ADMETlab 2.0.[1]

ADMET Property	Lower Bound	Upper Bound
Molecular Weight	100	600
Number of Hydrogen Bond Acceptors	0	12
Number of Hydrogen Bond Donors	0	7
Number of Rotatable Bonds	0	11
Number of Rings	0	6
Number of Heteroatoms	1	15
Formal Charge	-4	4
Topological Polar Surface Area	0	140
LogP	-0.4	6.5

Table S1.2. List of functional groups excluded by generation filter.

- ‘fr_azide’
- ‘fr_isocyan’
- ‘fr_isothiocyan’
- ‘fr_nitro’
- ‘fr_nitro_arom’
- ‘fr_nitro_arom_nonortho’
- ‘fr_nitroso’
- ‘fr_phos_acid’
- ‘fr_phos_ester’
- ‘fr_sulfonamd’
- ‘fr_sulfone’
- ‘fr_term_acetylene’
- ‘fr_thiocyan’
- ‘fr_prisulfonamd’
- ‘fr_C_S’
- ‘fr_azo’
- ‘fr_diazo’
- ‘fr_epoxide’
- ‘fr_ester’
- ‘fr_COO2’
- ‘fr_Imine’
- ‘fr_N_O’
- ‘fr_SH’
- ‘fr_aldehyde’
- ‘fr_dihydropyridine’
- ‘fr_hdrzine’
- ‘fr_hdrzone’
- ‘fr_ketone’
- ‘fr_thiophene’
- ‘fr_phenol’

<u>Section 2</u>: Similarity between FDA-Approved Inhibitors of C-Abl Kinase.

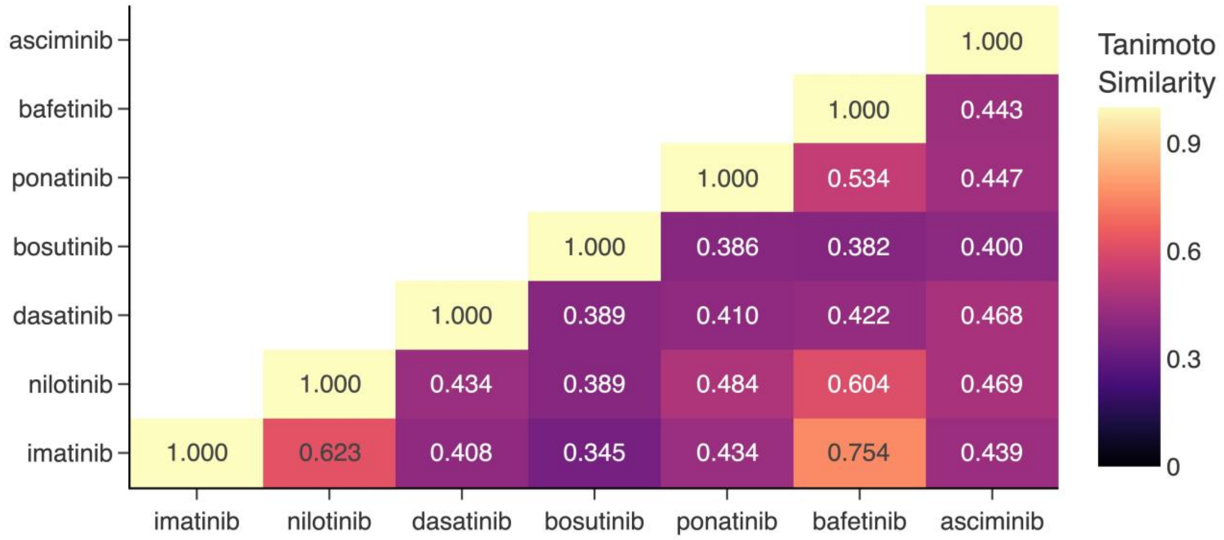

Figure S2.1. Tanimoto Similarity between RDKit fingerprints of the FDA-approved inhibitors of c-Abl kinase: imatinib, nilotinib, dasatinib, bosutinib, ponatinib, bafetinib, and asciminib.

<u>Section 3</u>: Comparing the Generations to c-Abl Kinase Inhibitors for Different Methods.

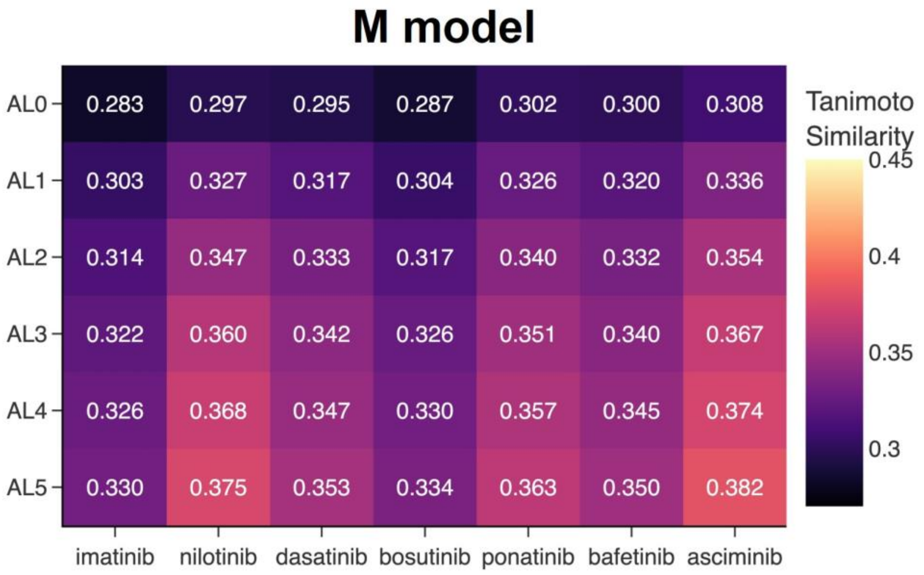

Figure S3.1. Visualizing the evolution of the generated molecular ensemble from the model pretrained on the MOSES dataset with the generation filtered based on ADMET metrics and functional group restrictions, and comparing it to the FDA-approved small-molecule inhibitors of c-Abl kinase. The average Tanimoto similarities between the RDKit fingerprints of all generated molecules at each iteration of the pipeline and each inhibitor are shown. Iteration 0 refers to the pretraining phase, while later iterations refer to the active learning phases

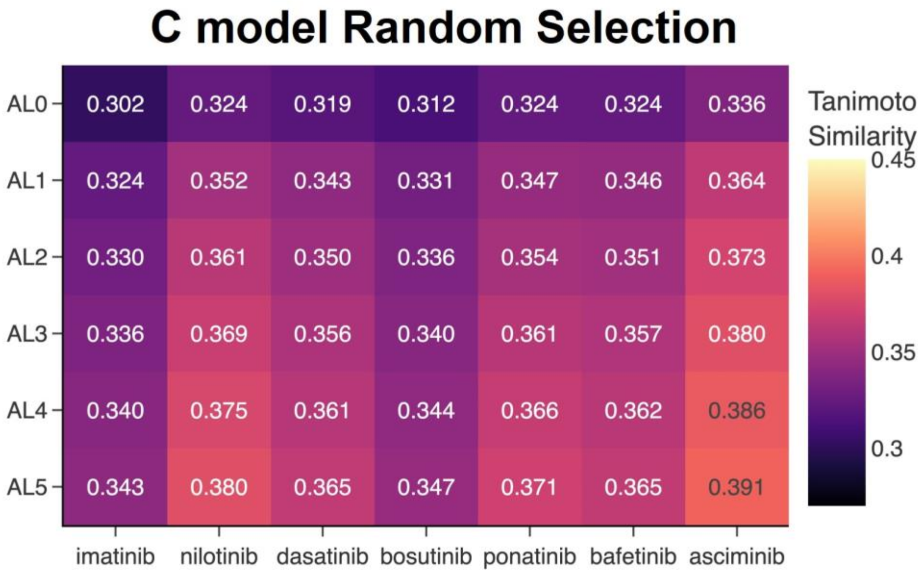

Figure S3.2. Visualizing the evolution of the generated molecular ensemble from the model utilizing random selection (i.e., 1,000 molecules are randomly selected from the generated ensemble and scored, and those that satisfy the score threshold and replicated *N* times where *N* is the smallest integer to achieve a total of 5,000 datapoints to be in the active learning set), pretrained on the combined dataset with the generation filtered based on ADMET metrics and functional group restrictions, and comparing it to the FDA-approved small-molecule inhibitors of c-Abl kinase. The average Tanimoto similarities between the RDKit fingerprints of all generated molecules at each iteration of the pipeline and each inhibitor are shown. Iteration 0 refers to the pretraining phase, while later iterations refer to the active learning phases

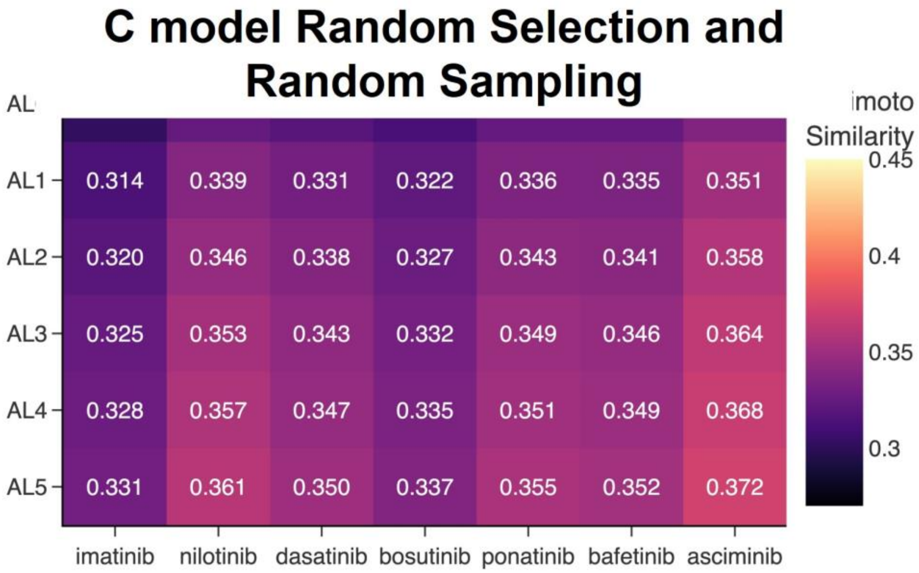

Figure S3.3. Visualizing the evolution of the generated molecular ensemble from the model utilizing random selection with random sampling (i.e., random selection with the addition of randomly sampling 5,000 molecules from the generated ensemble that have not been scored to be in the active learning training set), pretrained on the combined dataset with the generation filtered based on ADMET metrics and functional group restrictions, and comparing it to the FDA-approved small-molecule inhibitors of c-Abl kinase. The average Tanimoto similarities between the RDKit fingerprints of all generated molecules at each iteration of the pipeline and each inhibitor are shown. Iteration 0 refers to the pretraining phase, while later iterations refer to the active learning phases.

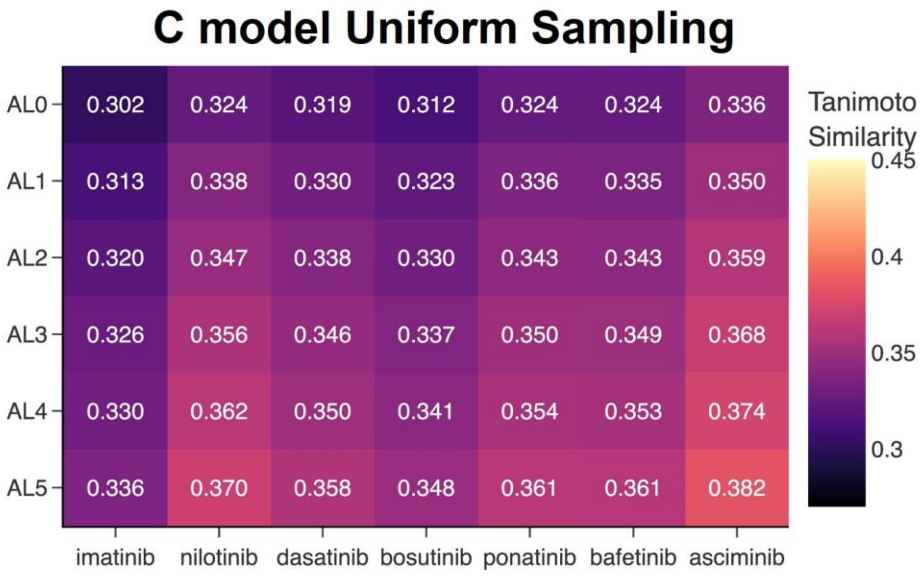

Figure S3.4. Visualizing the evolution of the generated molecular ensemble from the model utilizing uniform sampling (i.e., cluster-based sampling where each cluster is assigned a sampling fraction f = 0.01 to generate the active learning set), pretrained on the combined dataset with the generation filtered based on ADMET metrics and functional group restrictions, and comparing it to the FDA-approved small-molecule inhibitors of c-Abl kinase. The average Tanimoto similarities between the RDKit fingerprints of all generated molecules at each iteration of the pipeline and each inhibitor are shown. Iteration 0 refers to the pretraining phase, while later iterations refer to the active learning phases

Section 4: Radar Plots Showing Evolution of ADMET Metrics.

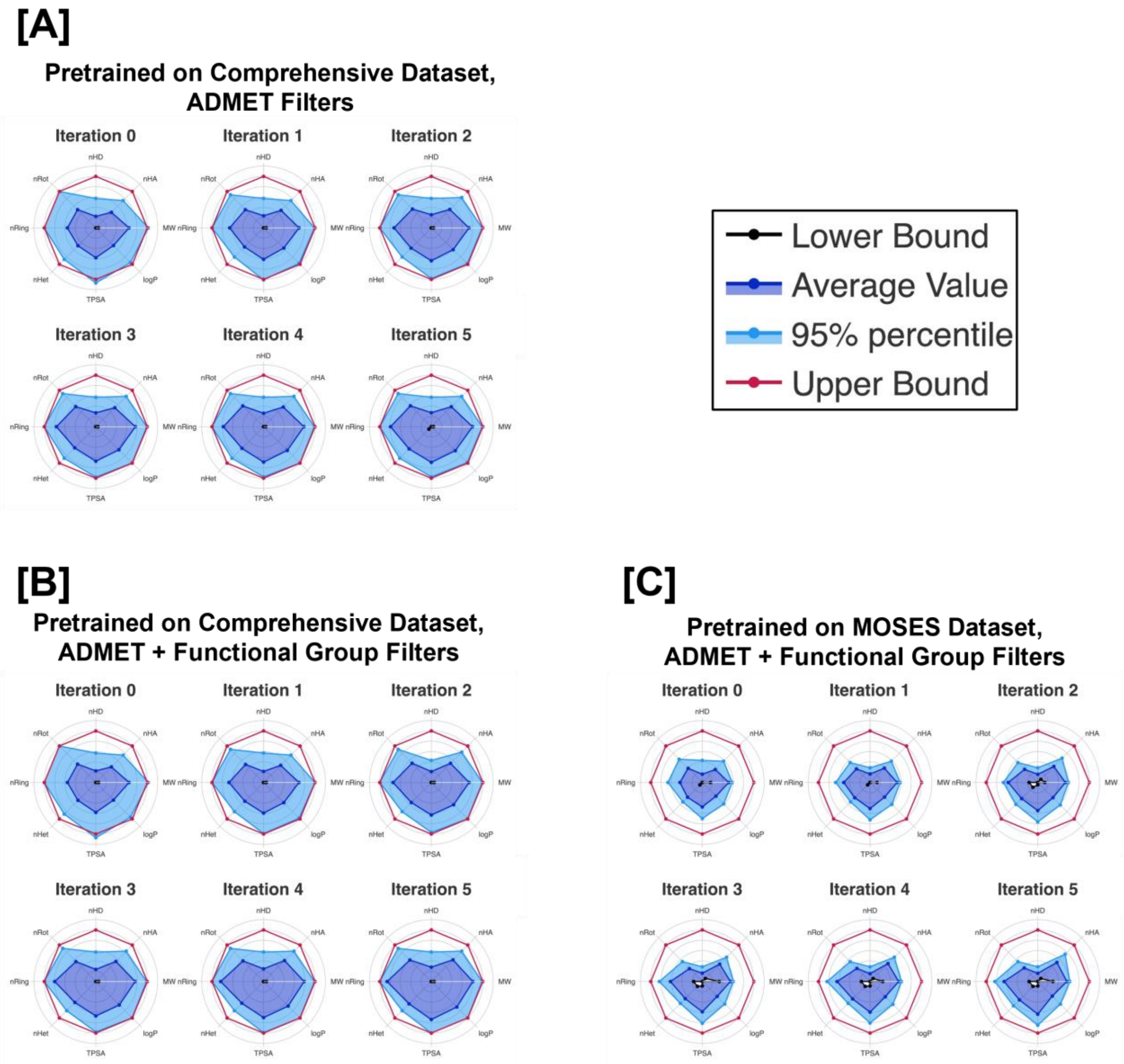

Figure S4.1. Radar charts for c-Abl kinase depicting the mean and 95th percentile values for each ADMET metric with respect to the lower and upper bounds enforced for the generated molecular ensembles from the model pretrained on the combined dataset with the generations filtered based on ADMET metrics are shown in (A), the charts for the ensembles from the model pretrained on the combined dataset with the generations filtered based on ADMET metrics and functional group restrictions are shown in (B), and the ensemble for the model pretrained on the MOSES dataset with the generations filtered based on ADMET metrics and functional group restrictions are shown in (C). Iteration 0 refers to the pretraining phase, while later iterations refer to the active learning phases.

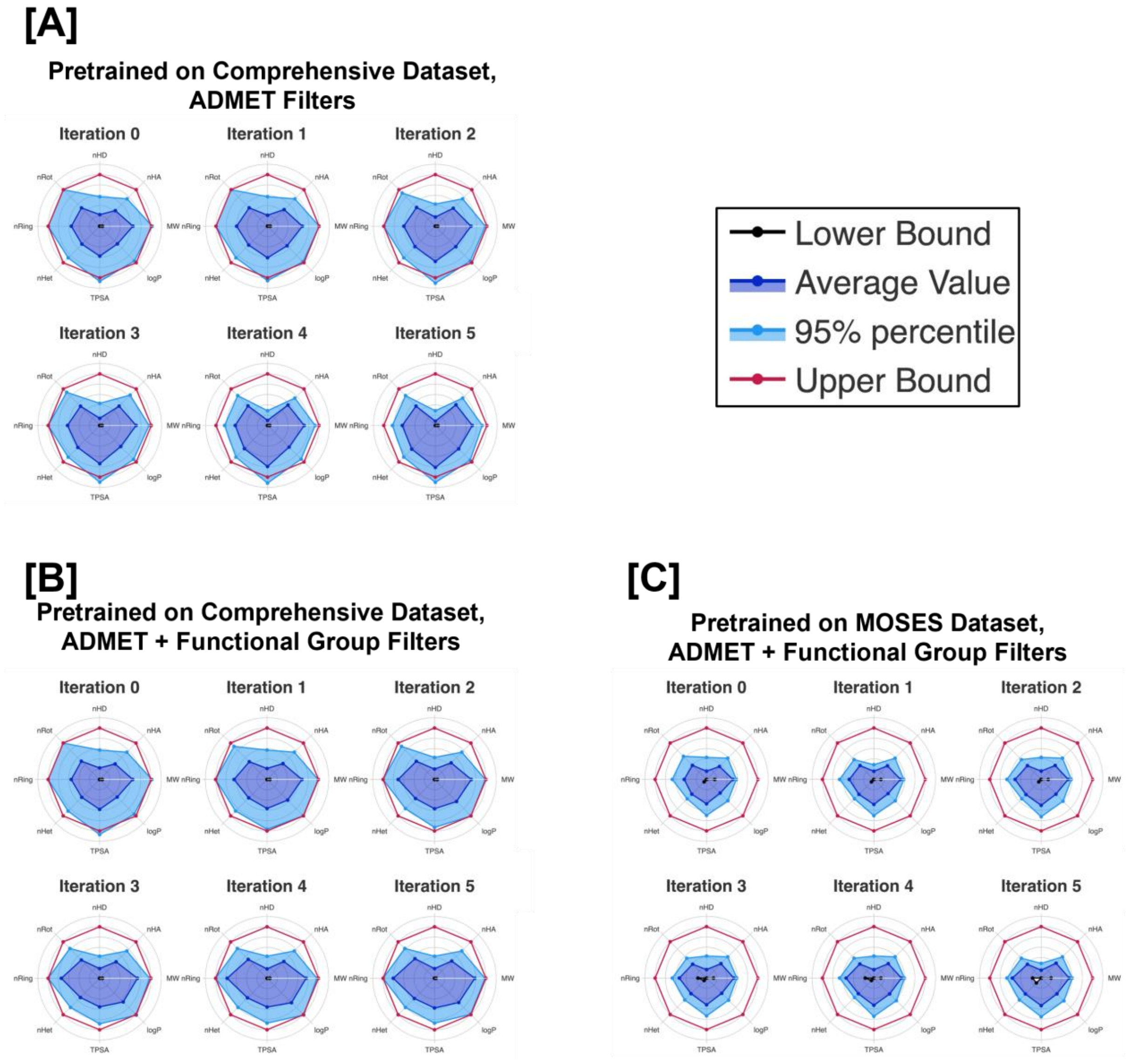

Figure S4.2. Radar charts for the HNH domain of Cas9 depicting the mean and 95th percentile values for each ADMET metric with respect to the lower and upper bounds enforced for the distribution for the model pretrained on the combined dataset with generation conditioned on ADMET filters are shown in (A), the distribution for the model pretrained on the combined dataset with generation conditioned on ADMET and functional group filters are shown in (B), and the distribution for the model pretrained on the MOSES dataset with generation conditioned on ADMET and functional group filters are shown in (C). Iteration 0 refers to the pretraining phase, while later iterations refer to the active learning phases.

Section 5: Scores of Molecules across Five Iterations of Active Learning for HNH.

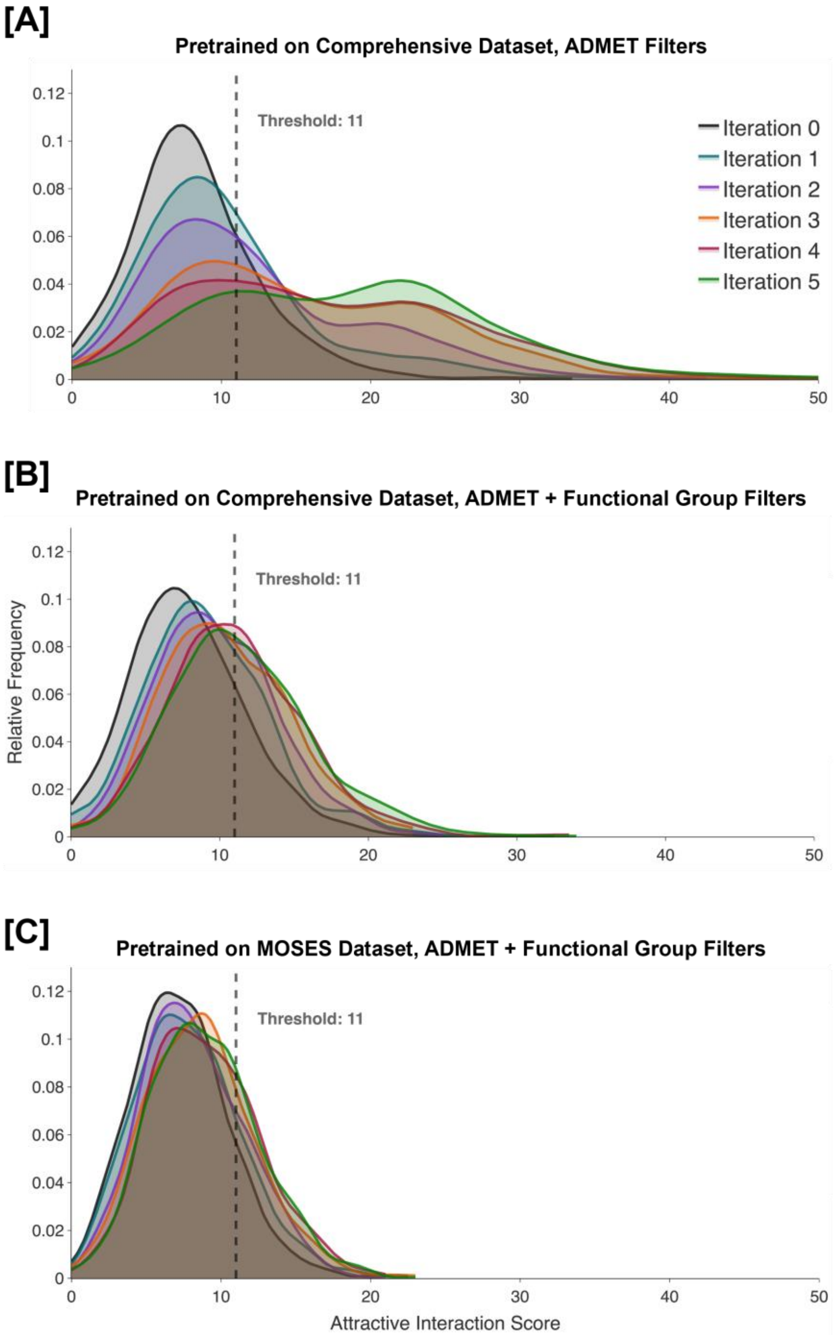

Figure S5.1. Attractive interaction scores of scored molecules across five iterations of active learning for the HNH domain of Cas9. The distribution for the model pretrained on the combined dataset with the generations filtered based on ADMET metrics are shown in (A). The distributions for the model pretrained on the combined dataset with the generations filtered based on ADMET metrics and functional group restrictions are shown in (B). The distributions for the model pretrained on the MOSES dataset with the generations filtered based on ADMET metrics and functional group restrictions are shown in (C). Iteration 0 refers to the pretraining phase, while later iterations refer to the active learning phases.

<u>Section 6</u>: Implementation Details of t-Distributed Stochastic Neighbor Embedding (t-SNE).

To create a standard t-SNE space which involves a constant coordinate system, we proceed as follows. Firstly, we collect scored molecules from all iterations (6,000 molecules). Secondly, we add the molecules from all active learning training sets that employed either softmax or uniform selection methods. Thirdly, we add a random sample of 10,000 molecules from the set of generations at each iteration. We perform this sampling to have the same number of molecules from the active learning training sets and generated sets, which enables us to fairly compute the difference in distributions. Note that our training sets usually contain slightly more than 10,000 molecules, so we sample exactly 10,000 for consistency. After combining all molecules and dropping all duplicates, we perform a t-SNE reduction.

<u>Section 7</u>: Choosing the Number of Clusters to Use for k-means.

For each implementation of k-means, we utilize the scikit-learn Python package,[21] which employs the k-means++ initialization algorithm, where the first centroid is selected randomly and subsequent centroids are iteratively chosen with a probability proportional to their squared distance from the nearest existing centroid

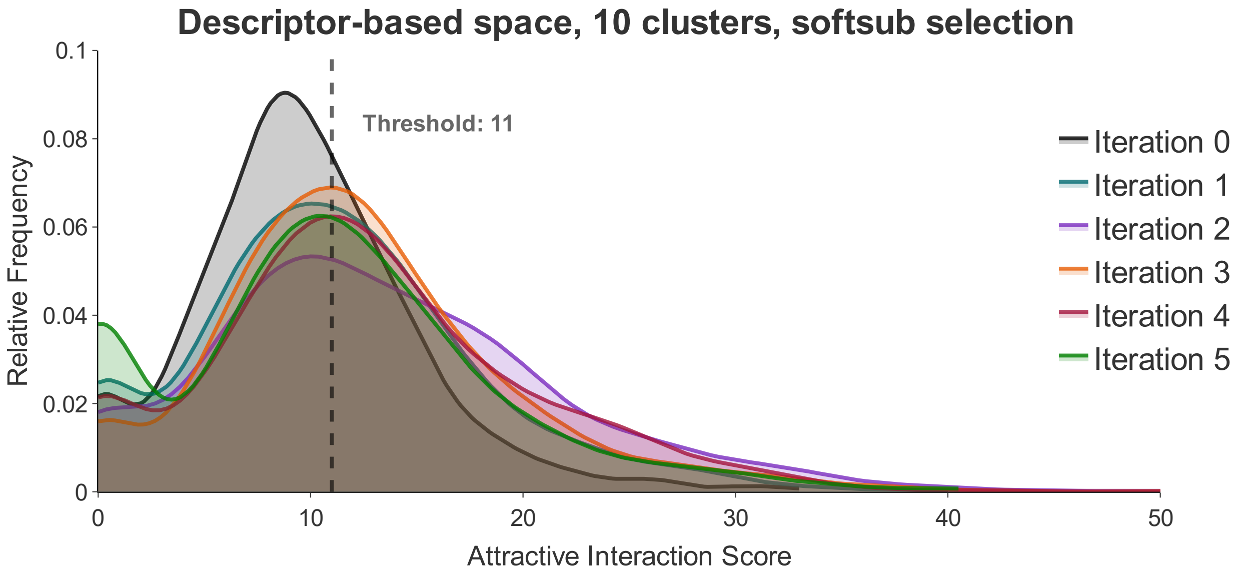

Figure S7.1. Attractive interaction scores for molecules generated by the pretrained model (iteration 0) and by the model after each of the five iterations of active learning where, prior to sampling for docking, molecules in the chemical space are grouped into 10 clusters. Cluster scores are converted into sampling fractions using the *softsub* approach.

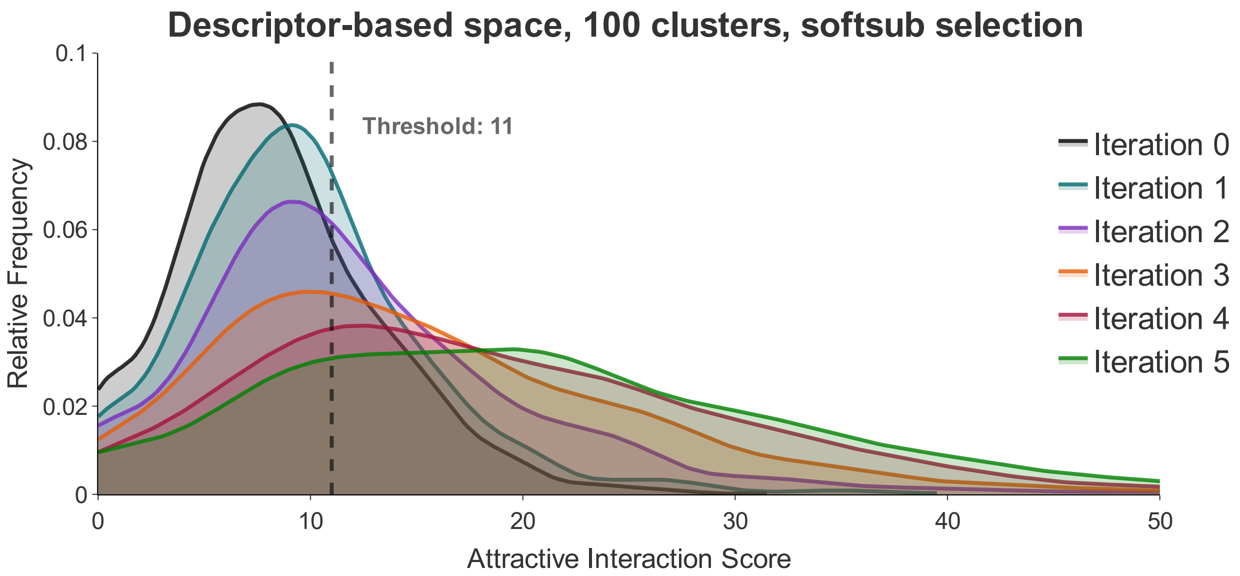

Figure S7.2. Attractive interaction scores for molecules generated by the pretrained model (iteration 0) and by the model after each of the five iterations of active learning where, prior to sampling for docking, molecules in the chemical space are grouped into 100 clusters. Cluster scores are converted into sampling fractions using the *softsub* approach. This figure occurs in the main text (Figure 4C), but is also shown here for comparison.

Table S7.3. Statistics of the distribution of attractive interaction scores, when molecules are clustered into 10 groups and cluster scores are converted into sampling fractions using the *softsub* method.

Iteration	Percent > 11	Q1	Q2	Mean	Q3	Max	Std
0	14.29	8.26	8.91	8.75	10.28	11.25	2.25
1	42.86	8.96	10.27	10.67	11.49	16.09	2.62
2	57.14	10.32	11.51	13.31	15.34	21.00	3.91
3	37.50	6.80	10.45	8.88	12.51	14.94	5.44
4	37.50	7.33	9.10	9.03	11.74	14.12	3.83
5	25.00	7.50	8.75	8.67	10.99	12.90	2.96

[a] The percentage of generated molecules with attractive interaction scores equal to or above our score threshold is shown (Percent > 11), as well as the score at the first quartile (Q1), second quartile (Q2), Mean, third quartile (Q3), maximum (Max), and standard deviation (Std) of the distribution.

[b] Iteration 0 refers to the pretraining phase, while later iterations refer to the active learning phases.

Table S7.4. Statistics of the distribution of attractive interaction scores, when molecules are clustered into 100 groups and cluster scores are converted into sampling fractions using the *softsub* method.

Iteration	Percent > 11	Q1	Q2	Mean	Q3	Max	Std
0	28.10	5.50	8.00	8.46	11.50	31.50	4.89
1	37.00	6.00	9.00	9.76	12.50	39.50	5.63
2	49.70	7.50	10.50	12.22	16.00	51.00	7.93
3	62.60	8.00	13.50	15.14	20.63	54.00	9.70
4	72.90	10.00	16.50	18.25	25.00	55.50	10.90
5	76.00	11.00	19.00	20.08	27.63	59.00	11.90

[a] The percentage of generated molecules with attractive interaction scores equal to or above our score threshold is shown (Percent > 11), as well as the score at the first quartile (Q1), second quartile (Q2), Mean, third quartile (Q3), maximum (Max), and standard deviation (Std) of the distribution.

[b] Iteration 0 refers to the pretraining phase, while later iterations refer to the active learning phases.

<u>Section 8</u>: Details and Parameters Used for Running DiffDock.

DiffDock handles all of the ligand preparation; we simply provide it with a protein structure and ligand SMILES string. It should be noted that since DiffDock is a diffusion generative model, it is inherently stochastic in nature During the docking inference stage, we utilize 20 inference steps, 10 samples for each complex, and a batch size of 6. Utilizing RDKit, DiffDock uses the MolFromSmiles module to process ligands, adds hydrogen atoms, and retrieves the 3D atomic coordinates with the AllChem.EmbedMolecule module employing the ETKDGv2 methodology.

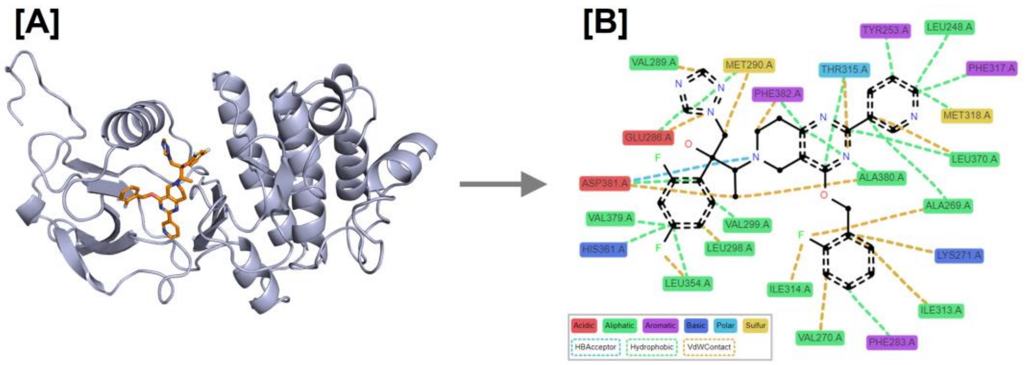

Figure S8.1. Generated molecule docked to the c-Abl kinase **(A)** with the corresponding protein-ligand fingerprint **(B)**.

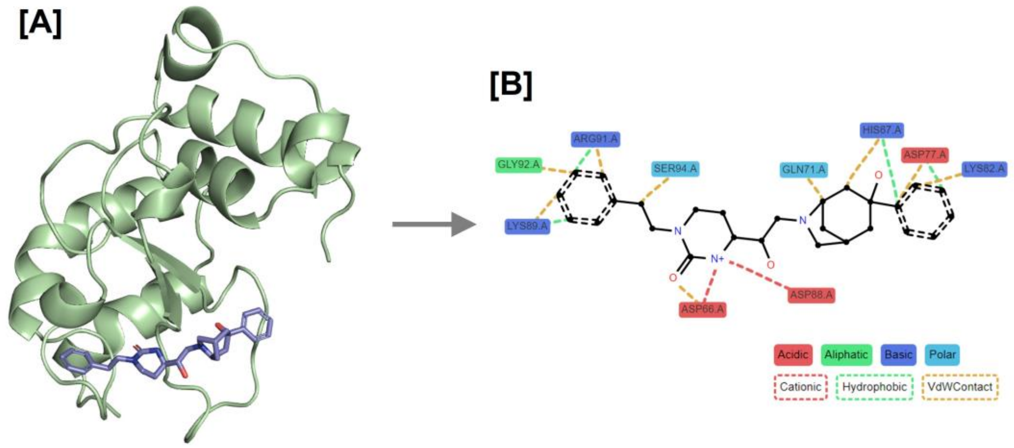

Figure S8.2. Generated molecule docked to the HNH domain of Cas9 **(A)** with the corresponding protein-ligand fingerprint **(B)**.

<u>Section 9</u>: t-SNE Visualization of the Evolution of the Generated Molecular Ensembles.

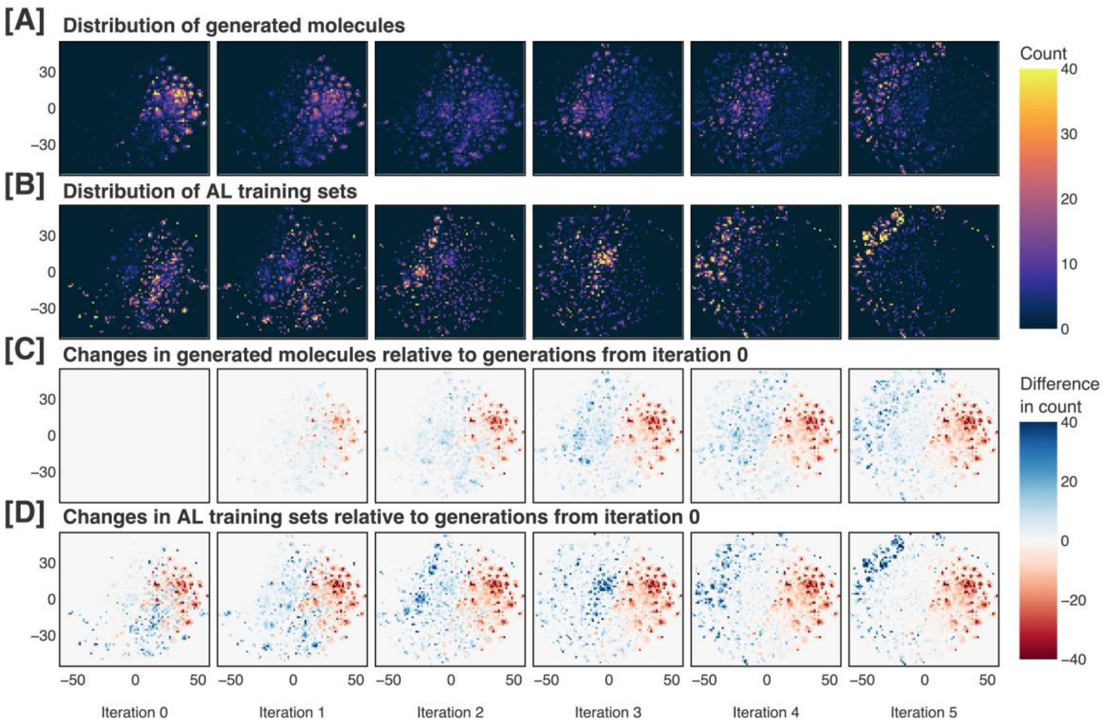

Figure S9.1. Generated molecules and active learning training sets across each iteration of our pipeline, visualized in two dimensions after performing t-distributed stochastic neighbor embedding (t-SNE). The generated molecules and active learning training sets are shown in (A) and (B), respectively. Changes in the generated molecules and active learning training sets relative to the molecules generated at iteration 0 are shown in (C) and (D), respectively. Iteration 0 refers to the pretraining phase, while later iterations refer to the active learning phases.

Section 10: Vocabulary Composition of the Combined Dataset.

Table S10.1. List of unique tokens that occur in the unfiltered combined dataset less than 1,000 times.

- '%10'
- '%11'
- '%12'
- '%13'
- '%14'
- '%15'
- '%16'
- '%17'
- '%18'
- '%19'
- '%20'
- '%21'
- '%22'
- '%23'
- '%24'
- '%25'
- '%26'
- '%27'
- '%28'
- '%29'
- '%30'
- '%31'
- '%32'
- '*'
- ':'
- '[*]'
- '[10B]'
- '[11C-]'
- '[11C@@H]'
- '[11CH2]'
- '[11CH3]'
- '[11CH]'
- '[11C]'
- '[11c]'
- '[123I-]'
- '[123IH]'
- '[123I]'
- '[123Te]'
- '[124I-]'
- '[124I]'
- '[125I-]'
- '[125IH]'
- '[125I]'
- '[127I]'
- '[127Xe]'
- '[129Xe]'
- '[131Cs]'
- '[131I-]'
- '[131I]'
- '[133Xe]'
- '[135I]'
- '[13CH2]'
- '[13CH3]'
- '[13CH]'
- '[13C]'
- '[13NH3]'
- '[13cH]'
- '[13c]'
- '[14C@@H]'
- '[14C@@]'
- '[14C@H]'
- '[14CH2]'
- '[14CH3]'
- '[14CH]'
- '[14C]'
- '[14cH]'
- '[14c]'
- '[15NH]'
- '[15OH2]'
- '[15nH]'
- '[15n]'
- '[17F]'
- '[18F-]'
- '[18FH]'
- '[18F]'
- '[18OH]'
- '[18O]'
- '[19F]'
- '[211At]'
- '[223Ra]'
- '[22Na+]'
- '[32PH]'
- '[32P]'
- '[35S]'
- '[3H]'
- '[42K+]'
- '[45Ca+2]'
- '[47Ca+2]'
- '[4H]'
- '[73Se]'
- '[75Se]'
- '[76BrH]'
- '[76Br]'
- '[81Kr]'
- '[82Rb+]'
- '[82Rb]'
- '[85Sr+2]'
- '[85SrH2]'
- '[89Sr+2]'
- '[Ag+]'
- '[Ag-4]'
- '[Ag-]'
- '[Ag]'
- '[Al+3]'
- '[Al-3]'
- '[Al]'
- '[Ar]'
- '[As+]'
- '[As-]'
- '[AsH3]'
- '[AsH]'
- '[As]'
- '[At]'
- '[Au-]'
- '[Au]'
- '[B@-]'
- '[B@@-]'
- '[BH-]'
- '[BH2-]'
- '[BH3-]'
- '[B]'
- '[Ba+2]'
- '[Ba]'
- '[Be+2]'
- '[Bi+3]'
- '[BiH3]'
- '[Bi]'
- '[Br+2]'
- '[Br]'
- '[C+]'
- '[CH+]'
- '[CH-]'
- '[CH2+]'
- '[CH2-]'
- '[CH2]'
- '[CH]'
- '[C]'
- '[Ca++]'
- '[Ca+2]'
- '[CaH2]'
- '[Ca]'
- '[Cl+2]'
- '[Cl+3]'
- '[Cl+]'
- '[Cl]'
- '[Co]'
- '[Cs+]'
- '[Cs]'
- '[Cu-]'
- '[Cu]'
- '[F+]'
- '[F-]'
- '[Fe++]'
- '[Fe--]'
- '[Fe-3]'
- '[Fe]'
- '[Gd-4]'
- '[Gd-5]'
- '[H+]'
- '[H-]'
- '[HH]'
- '[He]'
- '[Hg]'
- '[I+2]'

- '[I+3]'
- '[I+]'
- '[IH2]'
- '[IH]'
- '[I]'
- '[KH]'
- '[K]'
- '[Kr]'
- '[Li+]'
- '[LiH]'
- '[Li]'
- '[Mg+2]'
- '[Mg+]'
- '[MgH2]'
- '[Mg]'
- '[Mn]'
- '[Mo]'
- '[N@+]'
- '[N@@+]'
- '[N@@H+]'
- '[N@@]'
- '[N@H+]'
- '[N@]'
- '[NH-]'
- '[NH2+]'
- '[NH4+]'
- '[NH]'
- '[N]'
- '[NaH]'
- '[Na]'
- '[Nb--]'
- '[Ni++]'
- '[Ni]'
- '[O+]'
- '[O-2]'
- '[OH+]'
- '[OH-]'
- '[OH]'
- '[O]'
- '[Os]'
- '[P-]'
- '[P@+]'
- '[P@@+]'
- '[P@@]'
- '[P@]'
- '[PH+]'
- '[PH2+]'
- '[PH2]'
- '[PH]'
- '[P]'
- '[Pd--]'
- '[Pd]'
- '[Pt--]'
- '[Pt]'
- '[Ra]'
- '[Rb+]'
- '[Rb]'
- '[Re-]'
- '[Re]'
- '[Ru-]'
- '[Ru]'
- '[S-2]'
- '[S-]'
- '[S@+]'
- '[S@@+]'
- '[S@]'
- '[SH+]'
- '[SH-]'
- '[SH2]'
- '[SH]'
- '[S]'
- '[Sb]'
- '[Se+]'
- '[Se-2]'
- '[Se-]'
- '[SeH2]'
- '[SeH]'
- '[Si-]'
- '[Si@]'
- '[SiH-]'
- '[SiH2]'
- '[SiH3-]'
- '[SiH3]'
- '[SiH4]'
- '[SiH]'
- '[Sn]'
- '[Sr++]'
- '[Sr+2]'
- '[SrH2]'
- '[Tc]'
- '[Te+]'
- '[Te-]'
- '[TeH2]'
- '[TeH]'
- '[Te]'
- '[V]'
- '[W]'
- '[Xe]'
- '[Zn++]'
- '[Zn+2]'
- '[Zn+]'
- '[Zn-2]'
- '[Zn]'
- '[b-]'
- '[c+]'
- '[c-]'
- '[cH+]'
- '[cH-]'
- '[c]'
- '[n-]'
- '[nH+]'
- '[n]'
- '[o+]'
- '[o]'
- '[s+]'
- '[s]'
- '[se+]'
- '[te+]'
- '[te]'
- 'b'
- 'p'

Table S10.2. List of unique tokens that occur in the filtered combined dataset.

- '!'
- '#'
- '('
- ') '
- '-'
- '.'
- '/'
- '1'
- '2'
- '3'
- '4'
- '5'
- '6'
- '7'
- '8'
- '9'
- '<'
- '='
- 'B'
- 'Br'
- 'C'
- 'Cl'
- 'F'
- 'I'
- 'N'
- 'O'
- 'P'
- 'S'
- '[2H]'
- '[B-]'
- '[Br-]'
- '[C-]'
- '[C@@H]'
- '[C@@]'
- '[C@H]'
- '[C@]'
- '[Cl-]'
- '[H]'
- '[I-]'
- '[K+]'
- '[N+]'
- '[N-]'
- '[NH+]'
- '[NH3+]'
- '[Na+]'
- '[O-]'
- '[P+]'
- '[S+]'
- '[S@@]'
- '[Se]'
- '[Si]'
- '[n+]'
- '[nH]'
- '[se]'
- '\'
- 'c'
- 'n'
- 'o'
- 's'
- '~'

Section 11: Frequencies of Block Sizes, Molecular Weights and Tokens in Pretraining Sets.

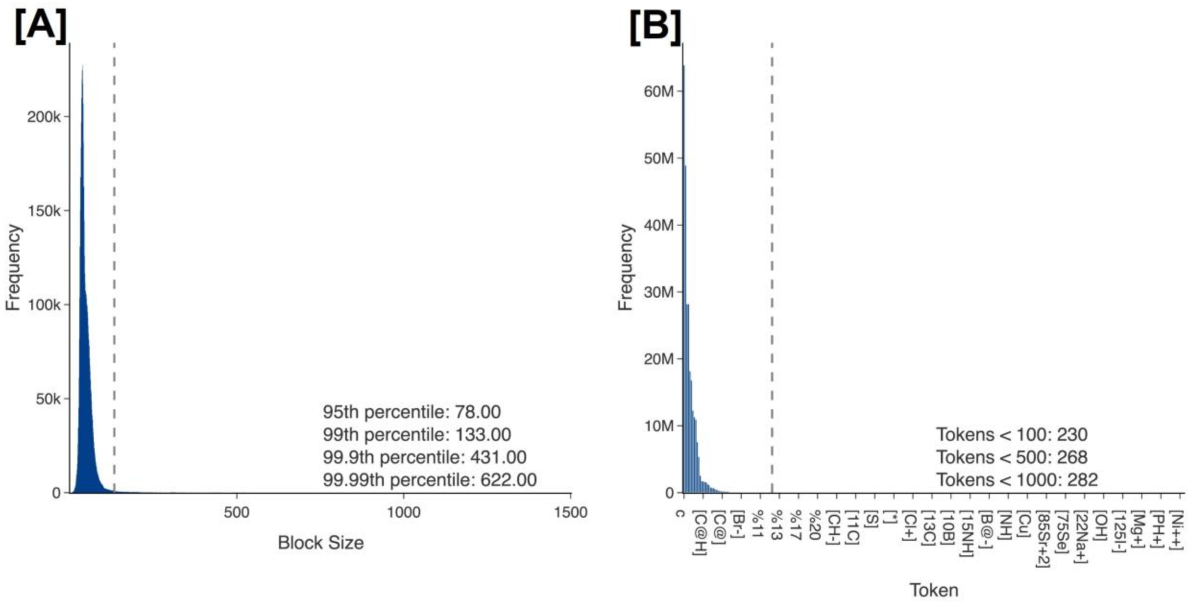

Figure S11.1. Frequency as a function of block size (A) and token (B). Vertical dotted lines are positioned at 133 in (A) and serves as our block size cutoff. In (B), our cutoff, illustrated with the vertical dotted lines, is positioned at the first token where the frequency is less than 1,000 times.

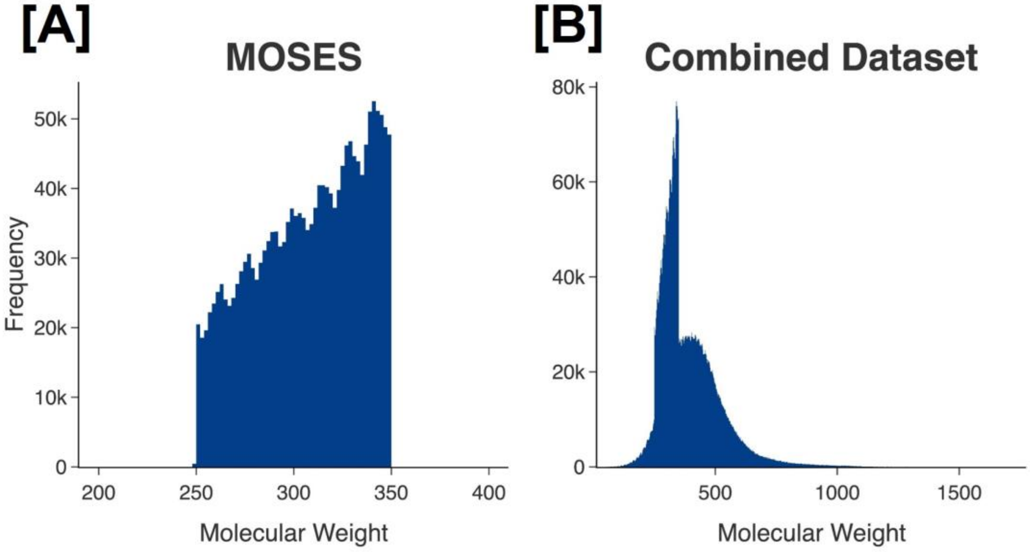

Figure S11.2. Frequency as a function of molecular weight of molecule in the (A) MOSES and (B) combined datasets.

Section 12: Details of the GPT Architecture.

The GPT model that we employ is based on the transformer architecture introduced in the revolutionary paper, "Attention is All You Need".**Error! Bookmark not defined.** Within the framework of the transformer architecture, the encoder processes input data into a sequence of context-rich vectors, while the decoder utilizes this contextual information to generate output data. Both of these
components utilize a self-attention mechanism, which enables the model to selectively focus on distinct parts of the input sequence at each computational step. The technical difference between the encoder and decoder parts of the transformer model is that the decoder ensures that the prediction for a particular token only depends on the preceding tokens, while each token in the encoder can attend to all other tokens in the sequence. Our GPT model is constructed as a series of transformer decoder blocks. This approach is appropriate for tasks that require generating novel sequential data such as SMILES strings.

The forward pass of our GPT model begins by dividing each SMILES string into distinct units known as tokens, processing each token with embedding layers, and combining these embeddings to form a vector representation of each token. These embedded vectors are then sequentially passed through a series of transformer decoder blocks, each comprised of a self-attention layer and a feed-forward network, with additional structural elements to enhance learning. The final result is a sequence of vectors, each corresponding to a position in the output SMILES string, where the elements of each vector represent probabilities for each token in the vocabulary. This high-level overview sets the stage for a more detailed discussion of the individual components.

Embeddings: Initially, a vocabulary comprising all of the unique tokens in the training data is constructed. For any given SMILES string in the input data, the input tokens undergo three distinct processing methods: token, positional, and type embeddings. The token embedding maps each token in the input sequence to a learnable vector representation, allowing the model to learn an optimal high-dimensional characterization for each token. Similarly, the positional embedding maps each input token to a learnable vector based on its position in the sequence. The type embedding layer uniformly assigns a constant bias to all embeddings of each input sequence. The sum of these three embeddings is passed through a dropout layer, setting 10% of its scalar components to 0. This embedding process transforms the input tokens into a form more suitable for the downstream modeling process.

Transformer Decoder Stack: For each token in the input sequence, the resulting embedding is passed to the first transformer decoder block, which begins with layer normalization, a process that adjusts and scales each embedding to have a mean of 0 and a standard deviation of 1. A self-attention mechanism is then applied to the normalized embedding, using learned matrices to linearly transform the embedding into three different vectors known as the query, key, and value vectors:

$$\mathbf{q}_i = \mathbf{W}_q \times \mathbf{e}_i \quad (1)$$

$$\mathbf{k}_i = \mathbf{W}_k \times \mathbf{e}_i \quad (2)$$

$$\mathbf{v}_i = \mathbf{W}_v \times \mathbf{e}_i \quad (3)$$

where $\mathbf{W}_q$, $\mathbf{W}_k$, and $\mathbf{W}_v$ are learned weight matrices that transform each input embedding, represented by $\mathbf{e}_i$, into the corresponding query, key, and value vectors. The dot products of the

query and each key vector are then scaled according to the dimensionality of the key vectors and passed through a softmax function, transforming them into a probability distribution to serve as attention weights. Finally, the attention scores are used to generate a weighted sum of the value vectors, as shown in the following equation:

$$\mathbf{e}'_i = \mathbf{V} \times \mathrm{softmax}\begin{pmatrix} \frac{\mathbf{q}_i \cdot \mathbf{k}_1}{\sqrt{\mathrm{d}_k}} \\ \cdots \\ \frac{\mathbf{q}_i \cdot \mathbf{k}_L}{\sqrt{\mathrm{d}_k}} \end{pmatrix} \quad (4)$$

Here, $\mathbf{e}'_i$ represents the output of the attention mechanism at position i in the sequence, $\mathbf{V}$ is the value matrix whose j^{th} column is the value vector corresponding to the embedding at position j in the sequence, d_k denotes the dimensionality of the key vectors, and L represents the length of the entire sequence. This operation amplifies the information from value vectors corresponding to higher attention weights (i.e., tokens that are more relevant to the current query), while suppressing the information from less relevant value vectors.

In practice, the self-attention mechanism is executed multiple times in parallel through what is known as *multi-head* attention. Each head (i.e., execution) uses its own set of learned linear transformations to generate query, key, and value vectors for all tokens in the sequence for each item in the batch, allowing the model to simultaneously focus on different aspects of the input across the various heads. The outputs from all attention heads are then concatenated and passed through a learned linear transformation to generate the final output of the multi-head attention mechanism.

A residual connection is a shortcut that skips one or more layers and allows the original input to be added directly to the output of those layers. This technique aids in training deeper networks by mitigating the vanishing gradient problem, where the gradients become too small for the network to learn effectively. In the context of GPT models, a residual connection is made by adding the input of the attention mechanism to the output. This sum is then processed using layer normalization, and the transformed embeddings are passed through a feed-forward network using the equation:

$$\mathbf{H} = \mathrm{Dropout}(\mathbf{W_2} \times \mathrm{GELU}(\mathbf{W_1} \times \mathbf{E}' + \mathbf{b_1}) + \mathbf{b_2}) \quad (5)$$

where $\mathbf{H}$ is the output of the feed-forward network, $\mathbf{E}'$ represents the matrix whose columns are the transformed embeddings, and $\mathbf{W}_1$ (shape: 1024×256), $\mathbf{b}_1$ (shape: 1024), $\mathbf{W}_2$ (shape: 256×1024), and $\mathbf{b}_2$ (shape: 256) represent the weight matrices and bias vectors of the two linear layers. GELU, or Gaussian Error Linear Unit, is an activation function used to introduce non-linearity into the model. A residual connection is established by summing the input to this feed-forward network with the output.

This entire process is repeated for additional decoder blocks, and the output of the final decoder block is processed with layer normalization. The normalized output is then passed through a learned linear transformation with bias to map the embeddings to the output vocabulary size, and

the resulting vectors are processed with softmax to generate the output probabilities at each position in the sequence.

<u>Section 13</u>: Training the GPT Model.

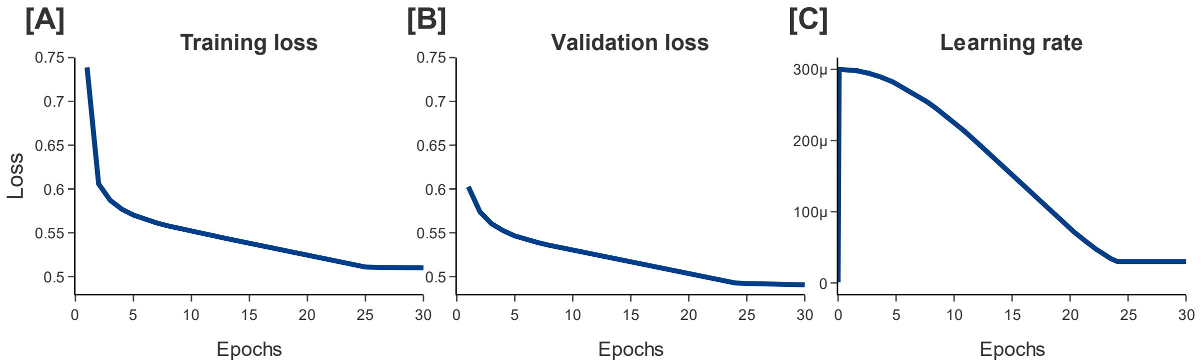

Figure S13.1. Training loss (A), validation loss (B), and learning rate (C) during the 30 epochs of pretraining of our model on the combined dataset.

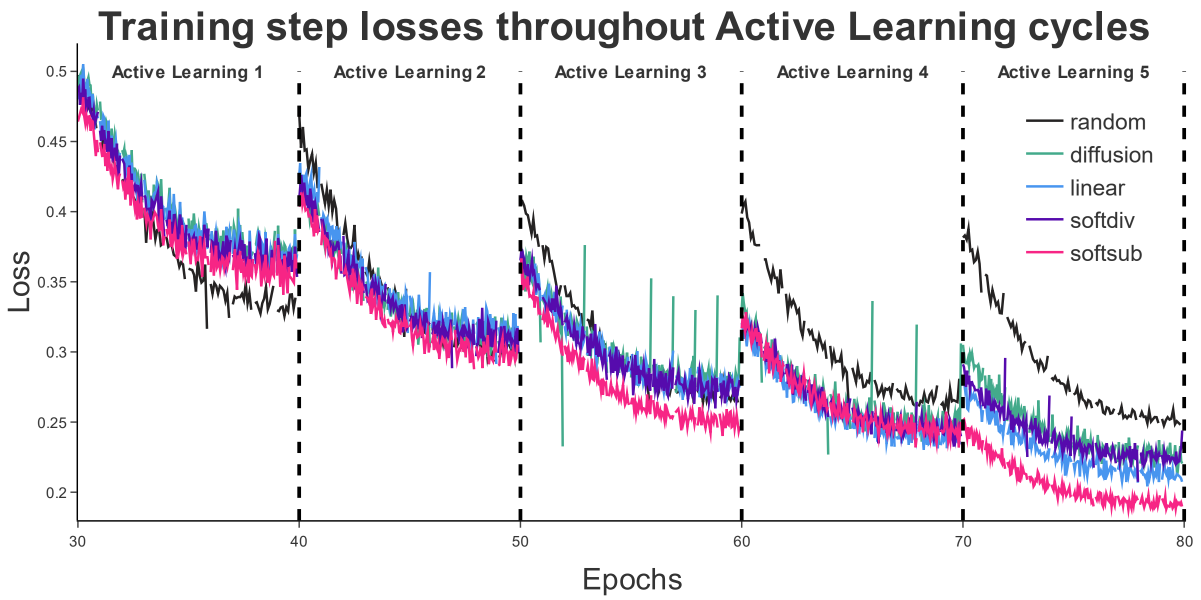

Figure S13.2. Training step losses (evaluated after each batch) during 5 rounds of active learning for the HNH domain of Cas9 (10 epochs each) with different conversion methods.

Section 14: Pretrained GPT Model Performance on the MOSES Benchmark.

Table S14.1. Primary results of our pretrained model on the MOSES benchmark compared to top-performing models in the field.

Model	Validity	Unique@1K	Unique@10K	Novelty	$IntDiv_1$	$IntDiv_2$	Filters
Our Model	0.996	**1.000**	0.999	0.730	0.856	0.850	**0.998**
MolGPT[2]	0.994	N/A	**1.000**	0.797	0.857	0.851	N/A
LatentGAN[3]	0.897	**1.000**	0.997	0.949	0.857	0.850	0.973
JT-VAE[4]	**1.000**	**1.000**	**1.000**	0.914	0.855	0.849	0.976
CharRNN[5]	0.975	**1.000**	0.999	0.842	0.856	0.850	0.994
MolecularRNN[6]	**1.000**	N/A	0.994	**1.000**	0.881	**0.876**	N/A
iPPIgAN[7]	0.989	**1.000**	0.999	0.990	N/A	N/A	N/A
DNMG[8]	0.999	**1.000**	0.998	0.936	0.856	0.850	0.996
CogMol[9]	0.955	**1.000**	**1.000**	N/A	0.857	0.851	0.989
TransVAE[10]	0.567	NA	N/A	0.996	N/A	N/A	N/A
ShapeProb[11]	0.969	**1.000**	0.995	N/A	0.865	N/A	0.865
GENTRL[12]	0.850	N/A	N/A	N/A	N/A	N/A	N/A
TransAntivirus[13]	**1.000**	0.999	0.999	0.999	**0.895**	N/A	N/A
CRTmaccs[14]	**1.000**	**1.000**	**1.000**	**1.000**	N/A	N/A	N/A
MolGCT[15]	0.985	**1.000**	0.998	0.814	0.853	N/A	0.996
cMolGPT[16]	0.988	**1.000**	0.999	N/A	N/A	N/A	N/A
GraphINVENT[17]	0.964	**1.000**	0.998	N/A	0.857	0.851	0.950
cTransformer[18]	0.988	**1.000**	0.999	N/A	N/A	N/A	N/A
GMTransformer[19]	0.829	**1.000**	**1.000**	0.883	0.856	N/A	0.980

[a] Validity (ratio of generated molecules deemed valid by RDKit's molecular structure parser), Unique@1K and @10K (fraction of valid generated molecules with no duplicates), Novelty (fraction of valid and unique generated molecules that are not in the training set), $IntDiv_i$ (internal diversity within the generated set for power mean *i*), and Filters (fraction of generated molecules that pass filters that check for specific fragments) are shown. See MOSES benchmark for more details on how these metrics are calculated.[20]

[b] The top value for each metric is shown in bold.

[c] Values not reported are shown as N/A.

Table S14.2. Additional results of our pretrained model on the MOSES benchmark compared to top-performing models in the field.

Model	FCD/Test	FCD/TestSF	Frag/Test	Frag/TestSF	SNN/Test	SNN/TestSF	Scaff/Test	Scaff/[illegible]
Our Model	**0.038**	**0.450**	**1.000**	**0.999**	0.633	**0.585**	**0.970**	0.[illegible]
MolGPT[2]	0.067	0.507	N/A	N/A	N/A	N/A	N/A	N[illegible]
LatentGAN[3]	0.296	0.824	0.999	0.998	0.538	0.514	0.886	0.[illegible]
JT-VAE[4]	0.395	0.938	0.997	0.995	0.548	0.519	0.896	0.[illegible]
CharRNN[5]	0.073	0.520	**1.000**	0.998	0.601	0.565	0.924	0.[illegible]
MolecularRNN[6]	N/A	N/A	N/A	N/A	N/A	N/A	N/A	N[illegible]
iPPIgAN[7]	5.879	6.171	N/A	N/A	N/A	N/A	N/A	N[illegible]
DNMG[8]	0.373	0.631	0.999	0.998	0.472	0.579	0.784	**0.**[illegible]
CogMol[9]	0.166	0.603	0.999	0.997	0.560	0. 533	0.905	0.[illegible]
TransVAE[10]	N/A	N/A	N/A	N/A	N/A	N/A	N/A	N[illegible]
ShapeProb[11]	1.332	1.850	0.984	0.980	0.446	0.432	0.459	0.[illegible]
GENTRL[12]	N/A	N/A	N/A	N/A	N/A	N/A	N/A	N[illegible]
TransAntivirus[13]	10.947	N/A	N/A	N/A	N/A	N/A	N/A	N[illegible]
CRTmaccs[14]	13.565	13.999	N/A	N/A	0.334	0.330	N/A	N[illegible]
MolGCT[15]	0.402	0.803	0.997	0.995	0.618	0.577	0.891	0.[illegible]
cMolGPT[16]	N/A	N/A	**1.000**	0.998	0.619	0.578	N/A	N[illegible]
GraphINVENT[17]	0.682	1.223	0.986	0.986	0.569	0.539	0.885	0.[illegible]
cTransformer[18]	N/A	N/A	**1.000**	0.998	0.619	0.578	N/A	N[illegible]
GMTransformer[19]	0.199	0.760	0.998	0.996	0.578	0.546	0.913	0.[illegible]

[a] FCD (Fréchet ChemNet Distance that is calculated using activation of the penultimate layer of ChemNet), Frag (compares molecular fragments between generated and training sets), SNN (average Tanimoto similarity between molecules in the generated set and the corresponding nearest molecule in the training set), and Scaff (compares molecular scaffolds between generated and training sets) are shown. Test (similarity from the training set to the test set) and TestSF (similarity from the training set to the scaffold test set) are shown for each metric. See MOSES benchmark for more details on how these metrics are calculated.[20]

[b] The top value for each metric is shown in bold.

[c] Values not reported are shown as N/A.

<u>Section 15</u>: RDKit Descriptors Used to Construct the Chemical Space Proxy.

Table S15.1. List of RDKit descriptors discarded.

- BCUT2D_CHGHI
- BCUT2D_CHGLO
- BCUT2D_LOGPHI
- BCUT2D_LOGPLOW
- BCUT2D_MRHI
- BCUT2D_MRLOW
- BCUT2D_MWHI
- BCUT2D_MWLOW
- Ipc
- MaxAbsPartialCharge
- MaxPartialCharge
- MinAbsPartialCharge
- MinPartialCharge

Table S15.2. List of RDKit descriptors included and used to construct the chemical space proxy.

- AvgIpc
- BalabanJ
- BertzCT
- Chi0
- Chi0n
- Chi0v
- Chi1
- Chi1n
- Chi1v
- Chi2n
- Chi2v
- Chi3n
- Chi3v
- Chi4n
- Chi4v
- EState_VSA1
- EState_VSA10
- EState_VSA11
- EState_VSA2
- EState_VSA3
- EState_VSA4
- EState_VSA5
- EState_VSA6
- EState_VSA7
- EState_VSA8
- EState_VSA9
- ExactMolWt
- FpDensityMorgan1
- FpDensityMorgan2
- FpDensityMorgan3
- FractionCSP3
- HallKierAlpha
- HeavyAtomCount
- HeavyAtomMolWt
- Kappa1
- Kappa2
- Kappa3
- LabuteASA
- MaxAbsEState Index
- MaxEStateIndex
- MinAbsEState Index
- MinEStateIndex
- MolLogP
- MolMR
- MolWt
- NHOHCount
- NOCount
- NumAliphatic Carbocycles
- NumAliphatic Heterocycles
- NumAliphatic Rings
- NumAromatic Carbocycles
- NumAromatic Heterocycles
- NumAromatic Rings
- NumHAcceptors
- NumHDonors
- NumHeteroatoms
- NumRadical Electrons
- NumRotatable Bonds
- NumSaturated Carbocycles
- NumSaturated Heterocycles
- NumSaturated Rings
- NumValence Electrons
- PEOE_VSA1
- PEOE_VSA10
- PEOE_VSA11
- PEOE_VSA12
- PEOE_VSA13
- PEOE_VSA14
- PEOE_VSA2
- PEOE_VSA3
- PEOE_VSA4
- PEOE_VSA5
- PEOE_VSA6
- PEOE_VSA7
- PEOE_VSA8
- PEOE_VSA9
- RingCount
- SMR_VSA1
- SMR_VSA10

- SMR_VSA2
- SMR_VSA3
- SMR_VSA4
- SMR_VSA5
- SMR_VSA6
- SMR_VSA7
- SMR_VSA8
- SMR_VSA9
- SlogP_VSA1
- SlogP_VSA10
- SlogP_VSA11
- SlogP_VSA12
- SlogP_VSA2
- SlogP_VSA3
- SlogP_VSA4
- SlogP_VSA5
- SlogP_VSA6
- SlogP_VSA7
- SlogP_VSA8
- SlogP_VSA9
- TPSA
- VSA_EState1
- VSA_EState10
- VSA_EState2
- VSA_EState3
- VSA_EState4
- VSA_EState5
- VSA_EState6
- VSA_EState7
- VSA_EState8
- VSA_EState9
- fr_Al_COO
- fr_Al_OH
- fr_Al_OH_noTert
- fr_ArN
- fr_Ar_COO
- fr_Ar_N
- fr_Ar_NH
- fr_Ar_OH
- fr_COO
- fr_COO2
- fr_C_O
- fr_C_O_noCOO
- fr_C_S
- fr_HOCCN
- fr_Imine
- fr_NH0
- fr_NH1
- fr_NH2
- fr_N_O
- fr_Ndealkylation1
- fr_Ndealkylation2
- fr_Nhpyrrole
- fr_SH
- fr_aldehyde
- fr_alkyl_carbamate
- fr_alkyl_halide
- fr_allylic_oxid
- fr_amide
- fr_amidine
- fr_aniline
- fr_aryl_methyl
- fr_azide
- fr_azo
- fr_barbitur
- fr_benzene
- fr_benzodiazepine
- fr_bicyclic
- fr_diazo
- fr_dihydropyridine
- fr_epoxide
- fr_ester
- fr_ether
- fr_furan
- fr_guanido
- fr_halogen
- fr_hdrzine
- fr_hdrzone
- fr_imidazole
- fr_imide
- fr_isocyan
- fr_isothiocyan
- fr_ketone
- fr_ketone_Topliss
- fr_lactam
- fr_lactone
- fr_methoxy
- fr_morpholine
- fr_nitrile
- fr_nitro
- fr_nitro_arom
- fr_nitro_arom_ nonortho
- fr_nitroso
- fr_oxazole
- fr_oxime
- fr_para_ hydroxylation
- fr_phenol
- fr_phenol_ noOrthoHbond
- fr_phos_acid
- fr_phos_ester
- fr_piperdine
- fr_piperzine
- fr_priamide
- fr_prisulfonamd
- fr_pyridine
- fr_quatN
- fr_sulfide
- fr_sulfonamd
- fr_sulfone
- fr_term_acetylene
- fr_tetrazole
- fr_thiazole
- fr_thiocyan
- fr_thiophene
- fr_unbrch_alkane
- fr_urea
- qed

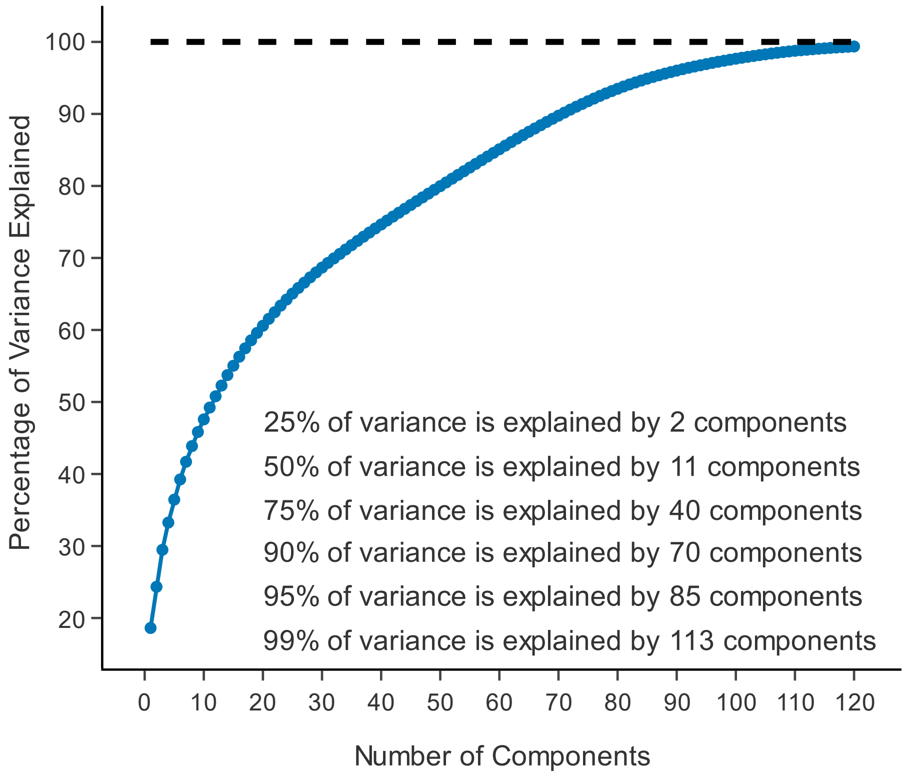

Figure S15.3. Cumulative fraction of variance explained by the first *N* principal components. Our chemical space proxy (i.e., the first 120 principal components) explains 99.3% of the variance in the hyperspace of 196 RDKit descriptors.

Section 16: Wall Times of Each Step in the Complete Pipeline.

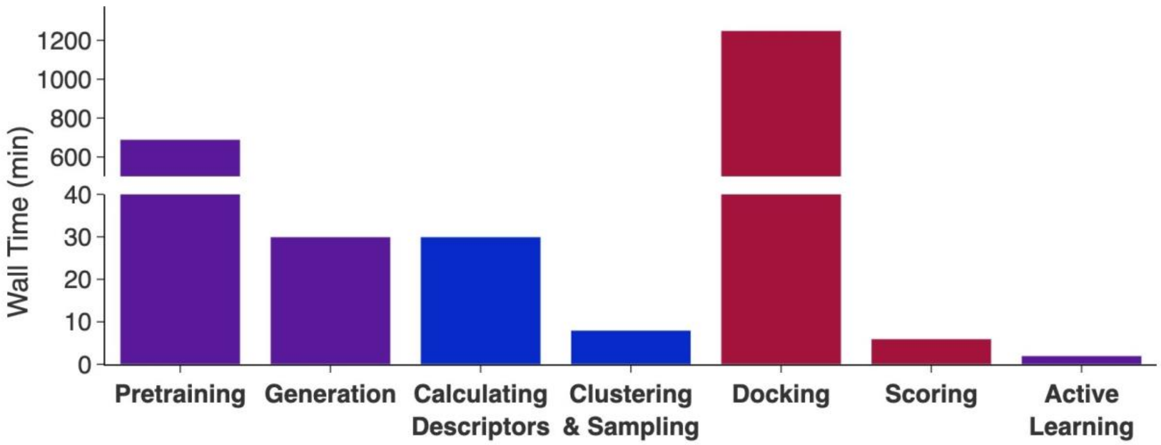

Figure S16.1. Wall times of each step in the complete pipeline. Steps include pretraining, generation, calculating descriptors, clustering and sampling, docking, scoring, and active learning fine-tuning. Pretraining is performed only once, while each other step is performed once per iteration.

<u>Section 17</u>: Evaluating the Methodology with Lower-Dimensional MQN Filters.

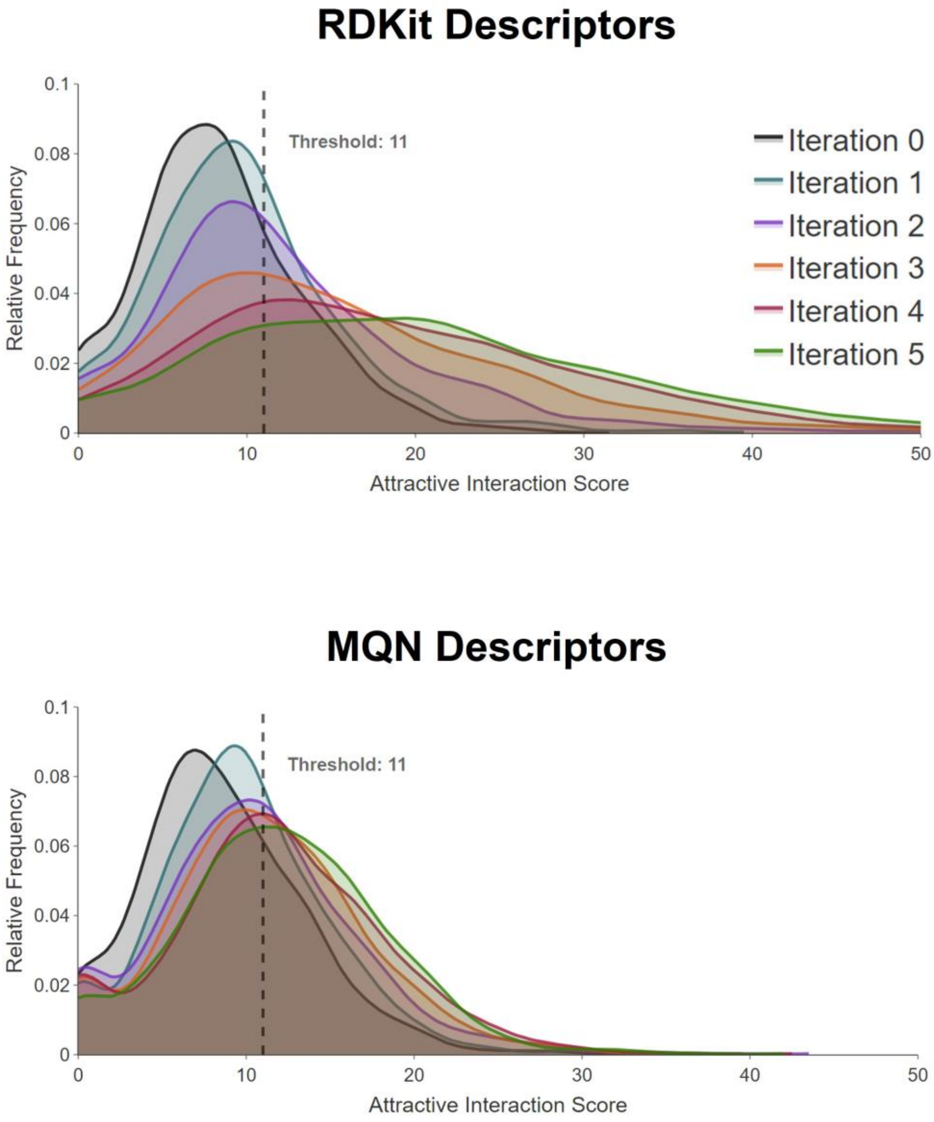

Figure S17.1. Attractive interaction scores of scored molecules across five iterations of active learning. Results for the methodology applied to the HNH domain of Cas9 using 196 RDKit descriptors, and using 42 MQN descriptors are shown. Iteration 0 refers to the pretraining phase, while later iterations refer to the active learning phases.

Section 18: Frequency as a Function of Cluster Size for Alignment to c-Abl Kinase.

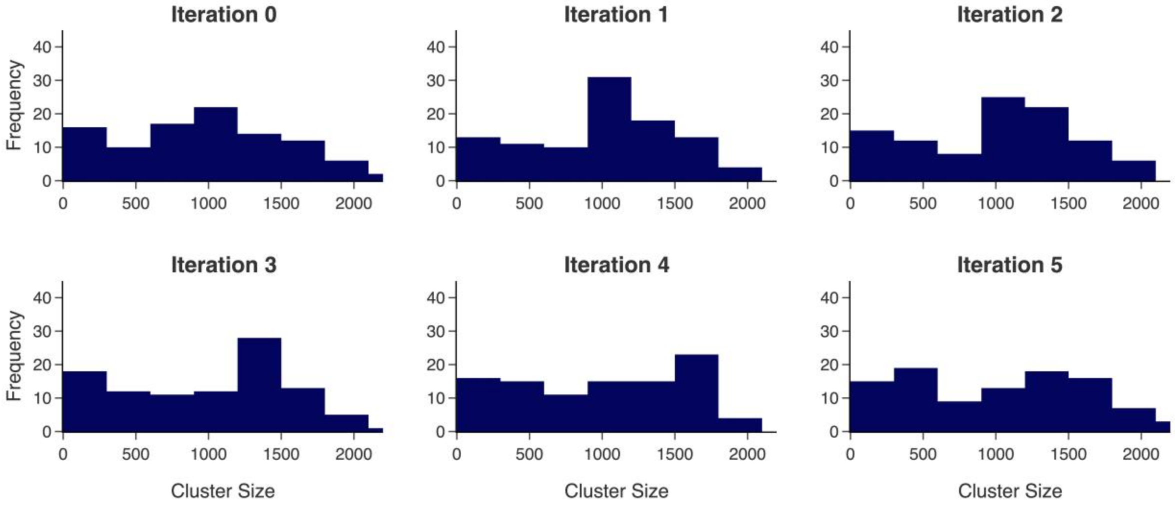

Figure S18.1. Frequency as a function of cluster size for each iteration of the methodology. Results are shown for the model pretrained on the combined dataset with the generations filtered based on ADMET metrics, aligned to c-Abl kinase.

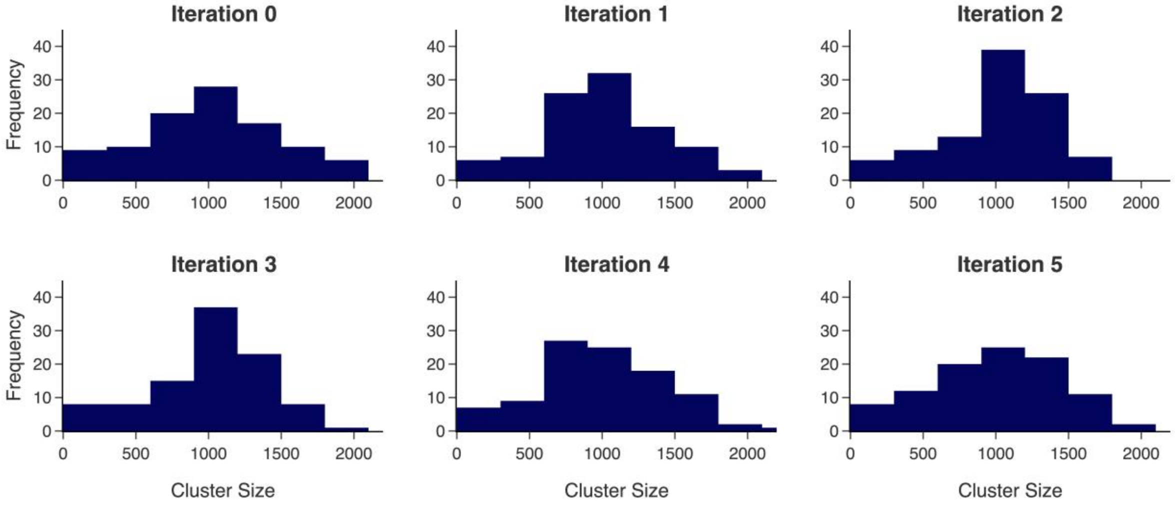

Figure S18.2. Frequency as a function of cluster size for each iteration of the methodology. Results are shown for the model pretrained on the combined dataset with the generations filtered based on ADMET metrics and functional group restrictions, aligned to c-Abl kinase.

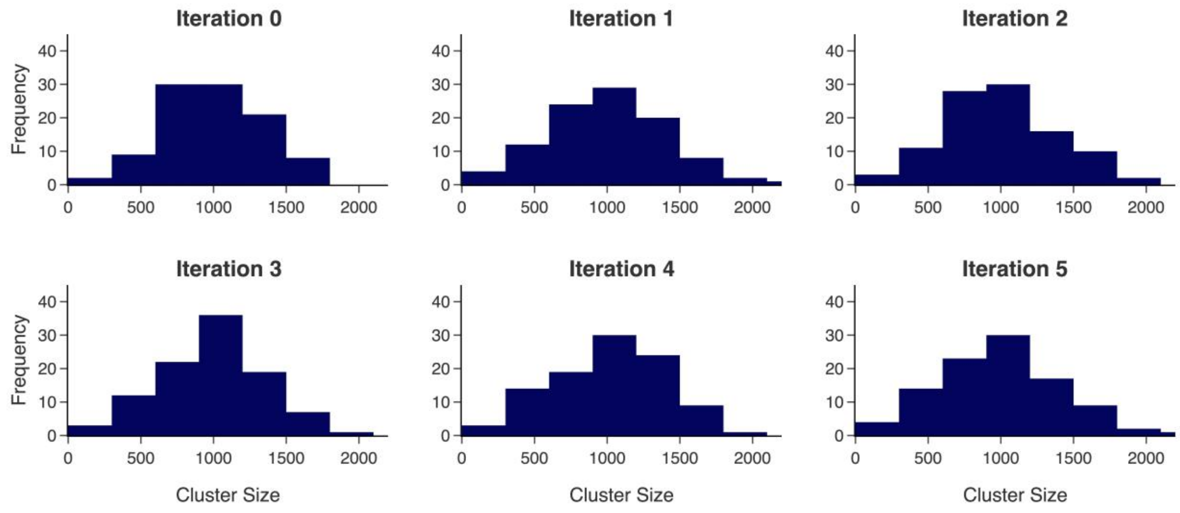

Figure S18.3. Frequency as a function of cluster size for each iteration of the methodology. Results are shown for the model pretrained on the MOSES dataset with the generations filtered based on ADMET metrics and functional group restrictions, aligned to c-Abl kinase.

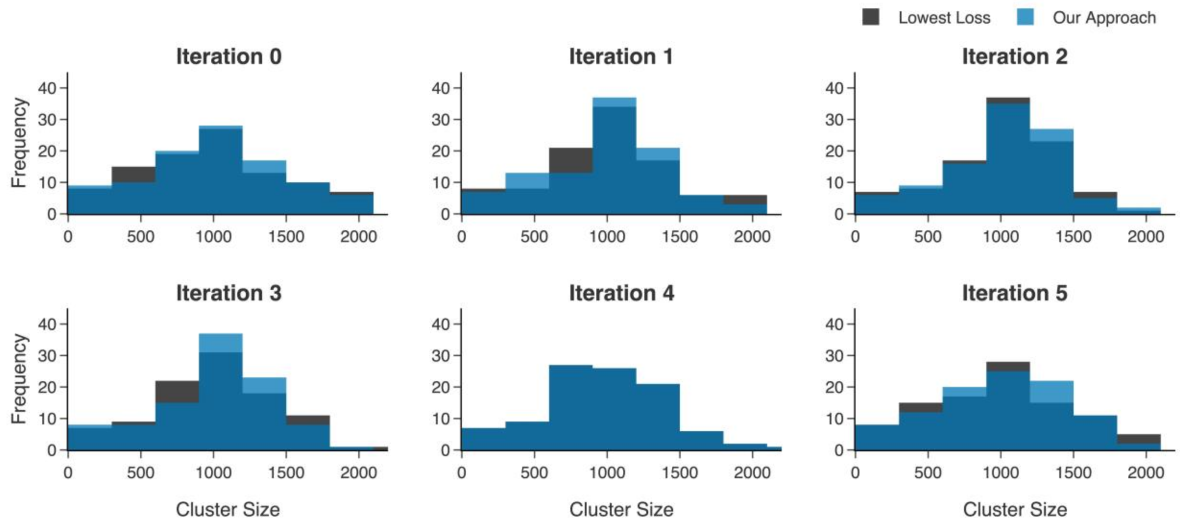

Figure S18.4. Frequency as a function of cluster size for each iteration of the methodology shown for the clustering that we select as well as the clustering with the lowest loss. Results are shown for the model pretrained on the combined dataset with the generations filtered based on ADMET metrics and functional group restrictions, aligned to c-Abl kinase.

Section 19: Evaluation of Scoring Function Compared to PDBbind v2020 Refined Set.

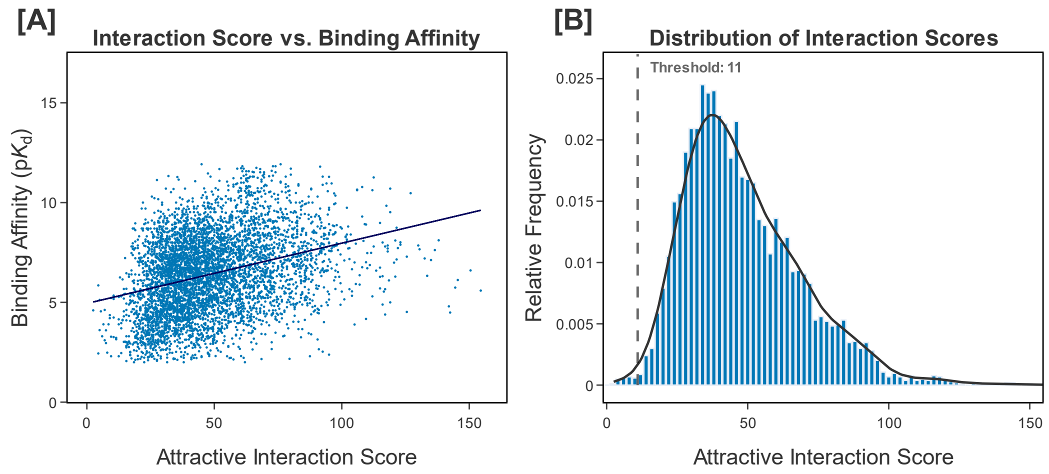

Figure S19.1. Evaluation of our scoring function with the protein-ligand complexes in the PDBbind v.2020 refined set. **(A)** Binding affinity (pK_d) plotted as a function of score. There is a corresponding Pearson correlation of 0.32. **(B)** The relative frequency of different scores is shown. 99.6% of the complexes exceed our score threshold of 11.

<u>Section 20</u>: Alternative Methods for Converting Mean Cluster Scores to Sampling Fractions.

Prior to constructing the active learning training set, we need to convert the attractive interaction scores s_i obtained by using the prolif software on docked molecules into sampling fractions f_i, which will be used to calculate the number of molecules that we need to sample from each cluster. A simple way to do that is to normalize the sum of all scores to unity:

$$f_i^{\text{linear}}(s_i) = \frac{s_i}{\sum_i s_i}$$

We call this approach *linear* conversion. Because one could interpret sampling fractions as effective probabilities of sampling from a given cluster, it is natural to consider the use of a softmax function:

$$f_i^{\text{softmax}}(s_i) = \frac{e^{s_i}}{\sum_i e^{s_i}}$$

which, for computational stability purposes, is often implemented with the maximum value among a set of arguments subtracted from each individual argument. To contrast with a modification of a softmax function introduced later, we refer to this as *softsub* conversion. In the main text of our paper, we implement the *softsub* approach and refer to it as *softmax* because this is the common implementation of the softmax function.

$$f_i^{\text{softsub}}(s_i) = \frac{e^{s_i - s_{max}}}{\sum_i e^{s_i - s_{max}}}$$

For a pretrained model, cluster scores range from 0 to 16. Because exponential functions increase rapidly, the *softsub* approach will effectively favor the 1-5 clusters with largest scores. We conjecture that a smoother function may lead to better model behavior during active learning, and implement a *softdiv* conversion approach, in which, instead of subtracting the maximum cluster score, we divide by it:

$$f_i^{\text{softdiv}}(s_i) = \frac{e^{s_i / s_{max}}}{\sum_i e^{s_i / s_{max}}}$$

Empirically, this approach leads even to a narrower distribution of sampling fractions than that obtained with the *linear* conversion approach. We introduce a hyperparameter $divf \in (0,1]$ by which we multiply the s_{max} value prior to dividing by it:

$$f_i^{\text{softdivf}}(s_i) = \frac{e^{\frac{s_i}{divf \times s_{max}}}}{\sum_i e^{\frac{s_i}{divf \times s_{max}}}}$$

By visualizing the distribution of *softdiv* values with different values of the hyperparameter (Figure S4.1), we pick $divf = 0.25$, as it maximizes the spread in sampling fractions. In what follows, the *softdiv* conversion will refer to *softdiv* with $divf = 0.25$.

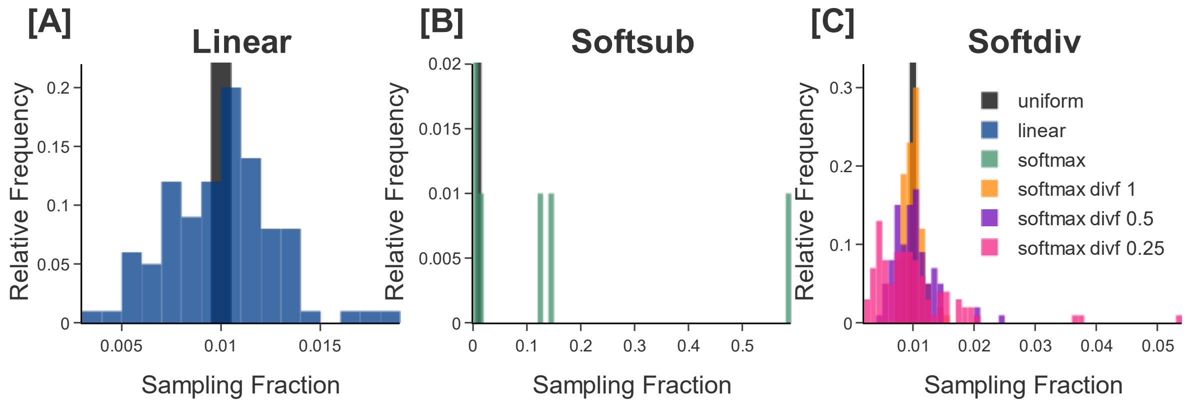

Figure S20.1. Distribution of sampling fractions obtained with different conversion approaches applied to cluster scores obtained from generations of the pretrained model. A bar corresponding to sampling the same number of molecules from each cluster (i.e., uniform sampling) is shown in black.

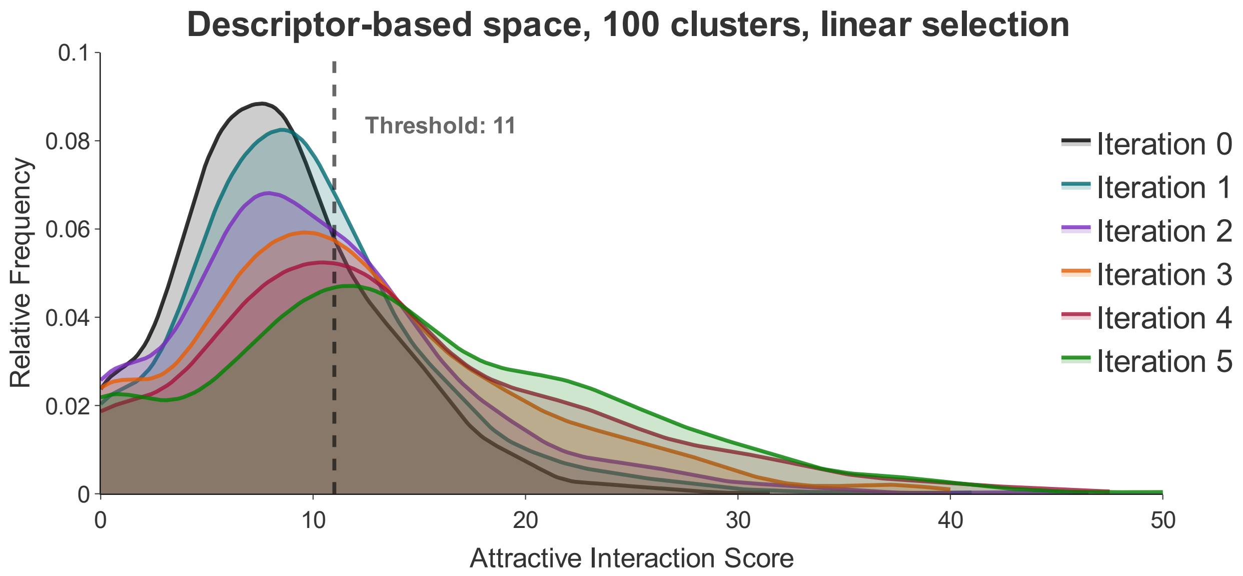

Figure S20.2. Attractive interaction scores for molecules generated by the pretrained model (iteration 0) and by the model after each of the five iterations of active learning where, prior to sampling for docking, molecules in the chemical space are grouped into 100 clusters, and cluster scores are converted into sampling fractions using the *linear* approach.

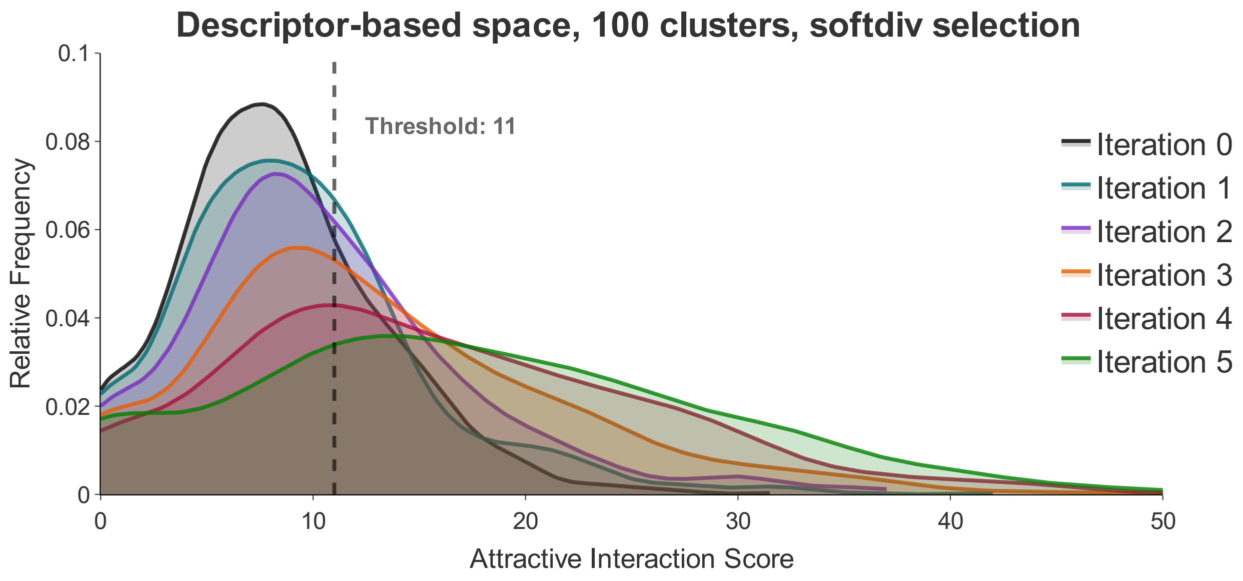

Figure S20.3. Attractive interaction scores for molecules generated by the pretrained model (iteration 0) and by the model after each of the five iterations of active learning where, prior to sampling for docking, molecules in the chemical space are grouped into 100 clusters, and cluster scores are converted into sampling fractions using the *softdiv* approach.

Table S20.4. Statistics of the distribution of attractive interaction scores, when molecules are selected randomly (naïve active learning), with no clustering.

Iteration	Percent > 11	Q1	Q2	Mean	Q3	Max	Std
0	26.20	5.50	8.00	8.41	11.00	33.00	4.58
1	32.40	5.50	9.00	9.27	11.50	35.00	4.79
2	35.00	6.50	9.00	9.67	12.50	33.00	4.89
3	40.00	6.88	9.50	10.45	13.50	37.00	5.31
4	44.80	7.00	10.00	11.03	13.63	42.00	6.16
5	44.20	7.00	10.00	11.13	13.50	38.50	6.05

[a] The percentage of generated molecules with attractive interaction scores equal to or above our score threshold is shown (Percent > 11), as well as the score at the first quartile (Q1), second quartile (Q2), Mean, third quartile (Q3), maximum (Max), and standard deviation (Std) of the distribution.

[b] Iteration 0 refers to the pretraining phase, while later iterations refer to the active learning phases.

[c] This table corresponds to the distribution in Figure 4A of main text.

Table S20.5. Statistics of the distribution of attractive interaction scores, when molecules are clustered into 100 groups and cluster scores are sampled *uniformly*.

Iteration	Percent > 11	Q1	Q2	Mean	Q3	Max	Std
0	12.00	6.68	8.58	8.43	9.85	15.80	2.31
1	24.00	7.75	9.10	9.17	10.88	20.80	3.40
2	30.00	8.17	9.40	9.96	11.50	21.50	3.41
3	35.00	7.86	10.07	10.23	11.63	24.00	3.97
4	50.00	8.88	10.89	11.28	13.46	25.45	4.11
5	50.00	8.80	10.94	11.84	14.48	25.80	4.63

[a] The percentage of generated molecules with attractive interaction scores equal to or above our score threshold is shown (Percent > 11), as well as the score at the first quartile (Q1), second quartile (Q2), Mean, third quartile (Q3), maximum (Max), and standard deviation (Std) of the distribution.

[b] Iteration 0 refers to the pretraining phase, while later iterations refer to the active learning phases.

[c] This table corresponds to the distribution in Figure 4B of main text.

Table S20.6. Statistics of the distribution of attractive interaction scores, when molecules are clustered into 100 groups and cluster scores are converted into sampling fractions using the *linear* method.

Iteration	Percent > 11	Q1	Q2	Mean	Q3	Max	Std
0	12.00	6.68	8.58	8.43	9.85	15.80	2.31
1	23.23	7.88	9.20	9.54	10.71	19.95	2.73
2	37.00	7.78	9.98	10.24	12.10	26.25	4.40
3	47.00	8.29	10.55	11.60	14.51	23.90	5.09
4	62.00	10.19	13.27	13.19	15.84	31.41	5.09
5	71.00	10.50	13.43	14.00	16.93	29.50	5.50

[a] The percentage of generated molecules with attractive interaction scores equal to or above our score threshold is shown (Percent > 11), as well as the score at the first quartile (Q1), second quartile (Q2), Mean, third quartile (Q3), maximum (Max), and standard deviation (Std) of the distribution.
[b] Iteration 0 refers to the pretraining phase, while later iterations refer to the active learning phases.
[c] This table corresponds to the distribution in Figure S4.1.

Table S20.7. Statistics of the distribution of attractive interaction scores, when molecules are clustered into 100 groups and cluster scores are converted into sampling fractions using the *softdiv* method.

Iteration	Percent > 11	Q1	Q2	Mean	Q3	Max	Std
0	28.10	5.50	8.00	8.46	11.50	31.50	4.89
1	34.70	5.50	8.50	9.29	12.50	42.00	5.89
2	42.20	6.50	9.50	10.54	13.50	37.00	6.64
3	54.20	7.50	11.50	13.07	18.00	55.00	8.64
4	65.90	8.50	14.25	15.82	22.00	56.50	9.99
5	71.20	9.50	16.00	17.32	24.50	51.00	10.90

[a] The percentage of generated molecules with attractive interaction scores equal to or above our score threshold is shown (Percent > 11), as well as the score at the first quartile (Q1), second quartile (Q2), Mean, third quartile (Q3), maximum (Max), and standard deviation (Std) of the distribution.
[b] Iteration 0 refers to the pretraining phase, while later iterations refer to the active learning phases.
[c] This table corresponds to the distribution in Figure S4.2.

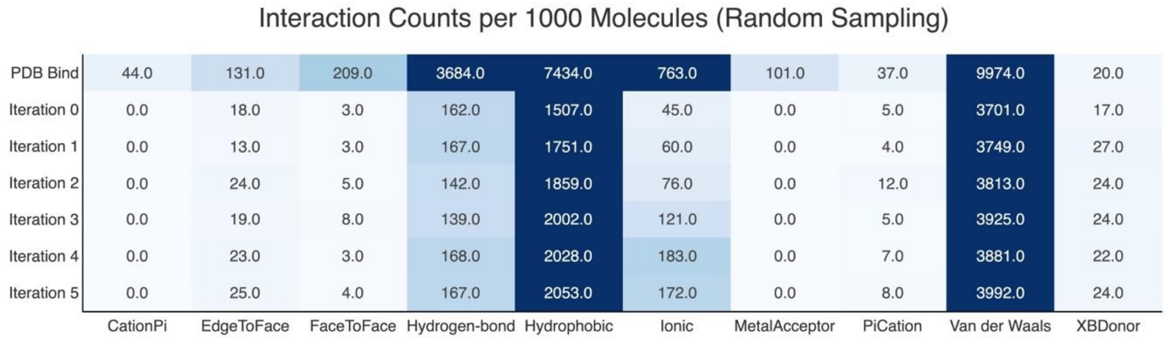

Figure S20.8. Counts of interactions of each type for 1000 scored molecules generated by the pretrained model (iteration 0) and by the model after each of the five rounds of naïve active learning with *random sampling*. A count of interactions from 1000 protein-ligand complexes randomly sampled from the refined set of PDBbind v2020 is included for comparison. These counts correspond to the score distribution in Figure 4A of main text.

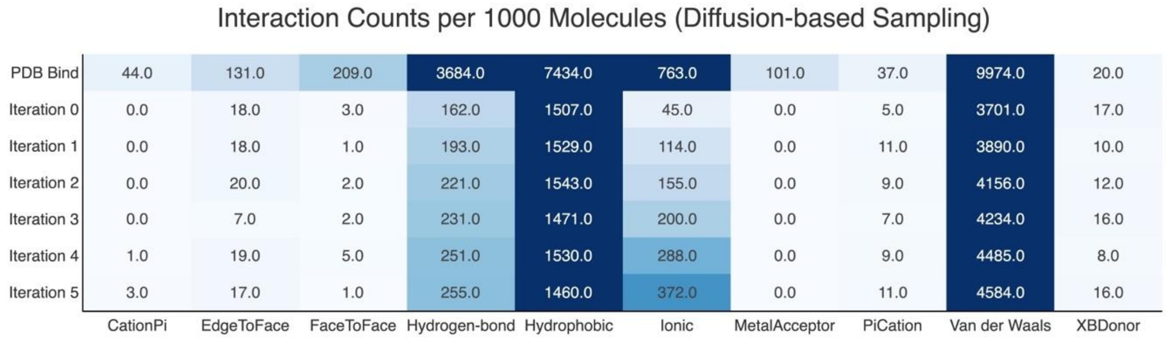

Figure S20.9. Counts of interactions of each type for 1000 molecules generated by the pretrained model (iteration 0) and by the model after each of the five rounds of active learning with clustering into 100 groups and *uniform* selection from each cluster. A count of interactions from 1000 protein-ligand complexes randomly sampled from the refined set of PDBbind v2020 is included for comparison. These counts correspond to the score distribution in Figure 4B of main text.

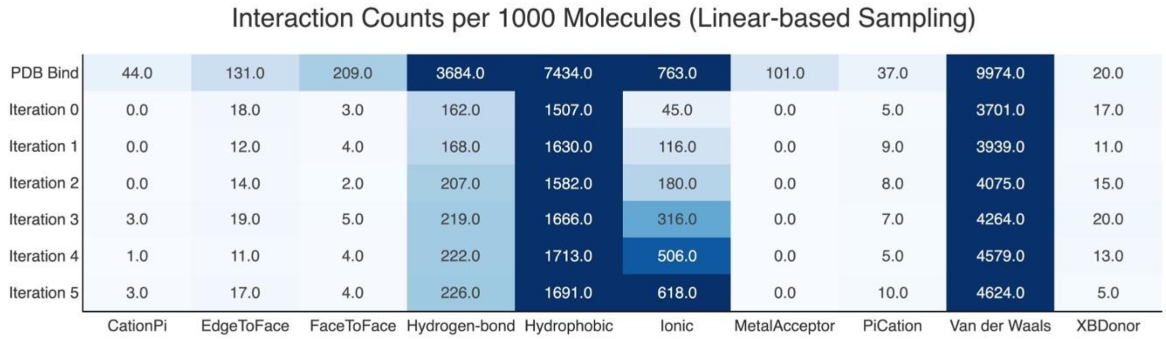

Figure S20.10. Counts of interactions of each type for 1000 scored molecules generated by the pretrained model (iteration 0) and by the model after each of the five rounds of active learning with clustering into 100 groups and conversion of cluster scores into sampling fractions using the *linear* method. A count of interactions from 1000 protein-ligand complexes randomly sampled from the refined set of PDBbind v2020 is included for comparison. These counts correspond to the score distribution in Figure S4.1.

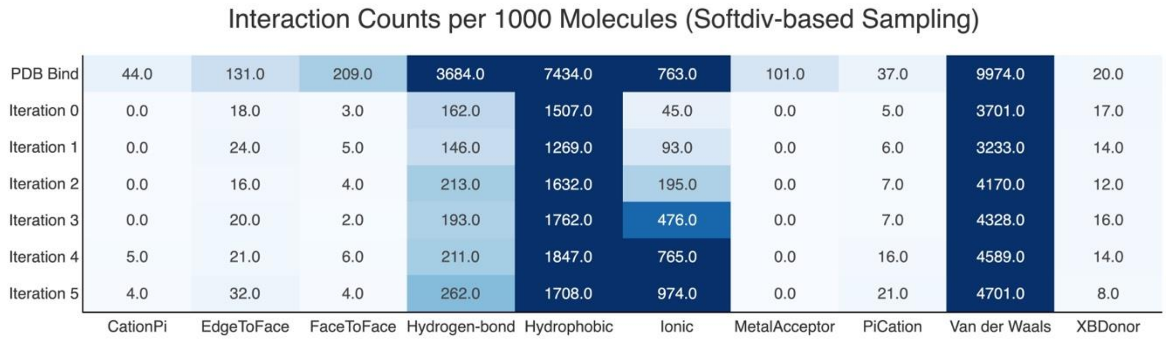

Figure S20.11. Counts of interactions of each type for 1000 scored molecules generated by the pretrained model (iteration 0) and by the model after each of the five rounds of active learning with clustering into 100 groups and conversion of cluster scores into sampling fractions using the *softdiv* method. A count of interactions from 1000 protein-ligand complexes randomly sampled from the refined set of PDBbind v2020 is included for comparison. These counts correspond to the score distribution in Figure S4.2.

Interaction Counts per 1000 Molecules (Softsub-based Sampling)

	CationPi	EdgeToFace	FaceToFace	Hydrogen-bond	Hydrophobic	Ionic	MetalAcceptor	PiCation	Van der Waals	XBDonor
PDB Bind	44.0	131.0	209.0	3684.0	7434.0	763.0	101.0	37.0	9974.0	20.0
Iteration 0	0.0	18.0	3.0	162.0	1507.0	45.0	0.0	5.0	3701.0	17.0
Iteration 1	0.0	17.0	2.0	185.0	1656.0	99.0	0.0	6.0	4134.0	18.0
Iteration 2	1.0	15.0	2.0	187.0	1830.0	347.0	0.0	8.0	4321.0	7.0
Iteration 3	1.0	23.0	2.0	176.0	1857.0	724.0	0.0	10.0	4352.0	15.0
Iteration 4	1.0	19.0	5.0	152.0	1954.0	1104.0	0.0	6.0	4452.0	15.0
Iteration 5	2.0	39.0	5.0	161.0	2026.0	1291.0	0.0	8.0	4653.0	12.0

Figure S20.12. Counts of interactions of each type for 1000 molecules generated by the pretrained model (iteration 0) and by the model after each of the five rounds of active learning with clustering into 100 groups and conversion of cluster scores into sampling fractions using the *softsub* method. A count of interactions from 1000 protein-ligand complexes randomly sampled from the refined set of PDBbind v2020 is included for comparison. These counts correspond to the score distribution in Figure 4C of the main text.

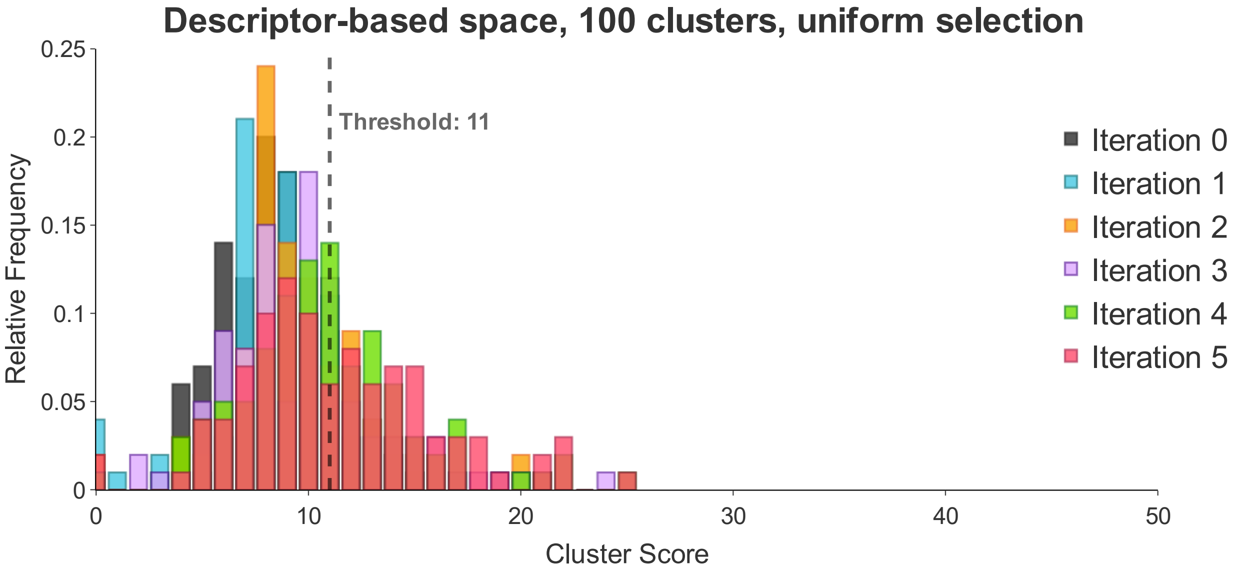

Figure S20.13. Cluster scores (obtained as an average of attractive interaction scores for molecules in the cluster) for molecules generated by the pretrained model (iteration 0) and by the model after each of the five iterations of active learning where, prior to sampling for docking, molecules in the chemical space are grouped into 100 clusters, and molecules are sampled from each cluster *uniformly*. These cluster scores correspond to score distribution in Figure 4B of the main text.

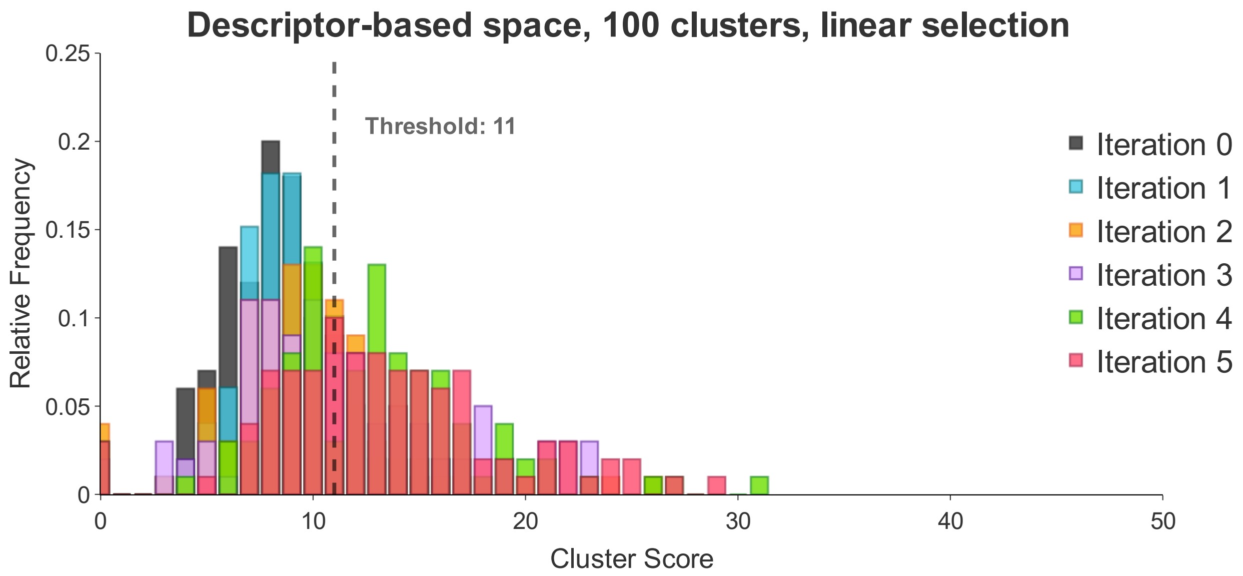

Figure S20.14. Cluster scores (obtained as an average of attractive interaction scores for molecules in the cluster) for molecules generated by the pretrained model (iteration 0) and by the model after each of the five iterations of active learning where, prior to sampling for docking, molecules in the chemical space are grouped into 100 clusters, and molecules are sampled from each cluster using the *linear* method. These cluster scores correspond to score distribution in Figure SI4.1.

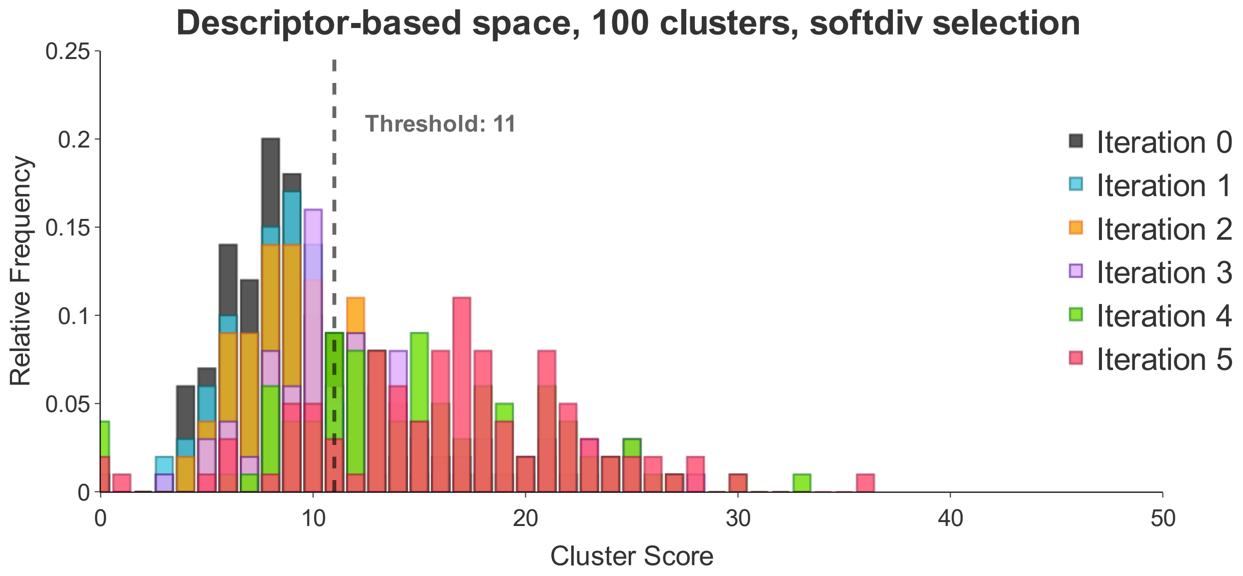

Figure S20.15. Cluster scores (obtained as an average of attractive interaction scores for molecules in the cluster) for molecules generated by the pretrained model (iteration 0) and by the model after each of the five iterations of active learning where, prior to sampling for docking, molecules in the chemical space are grouped into 100 clusters, and molecules are sampled from each cluster using the *softdiv* method. These cluster scores correspond to score distribution in Figure SI4.2.

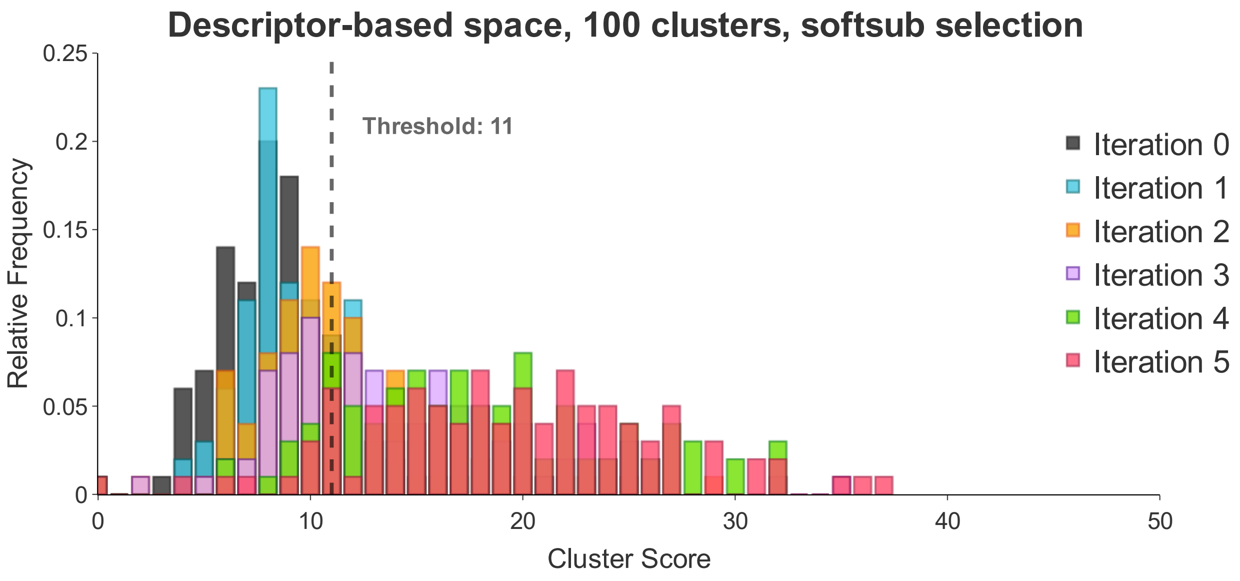

Figure S20.16. Cluster scores (obtained as an average of attractive interaction scores for molecules in the cluster) for molecules generated by the pretrained model (iteration 0) and by the model after each of the five iterations of active learning where, prior to sampling for docking, molecules in the chemical space are grouped into 100 clusters, and molecules are sampled from each cluster using the *softsub* method. These cluster scores correspond to score distribution in Figure 4C of the main text.

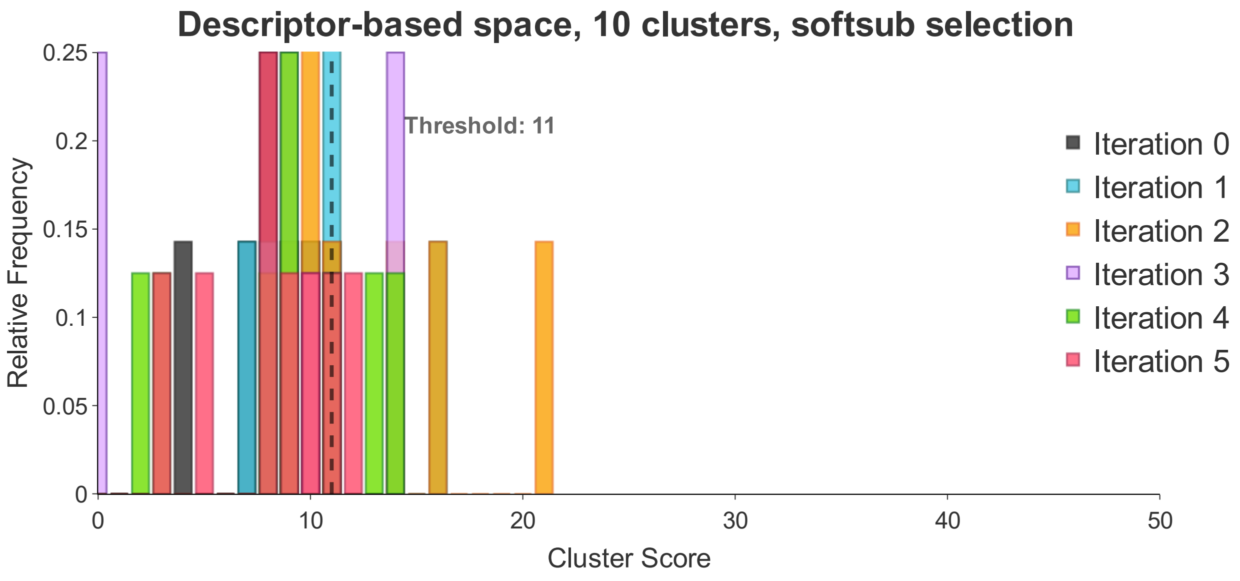

Figure S20.17. Cluster scores (obtained as an average of attractive interaction scores for molecules in the cluster) for molecules generated by the pretrained model (iteration 0) and by the model after each of the five iterations of active learning where, prior to sampling for docking, molecules in the chemical space are grouped into 10 clusters, and molecules are sampled from each cluster using the *softsub* method. These cluster scores correspond to score distribution in Figure S10.1 of the main text.

Section 21: Distributions of Mean and Median Cluster Scores.

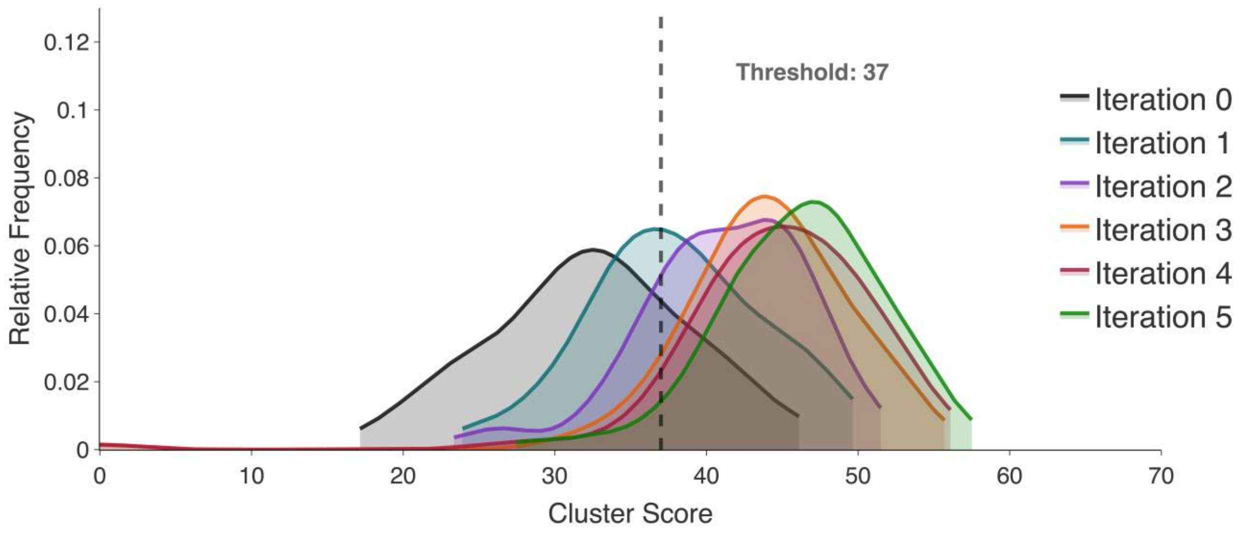

Figure S21.1. Mean cluster attractive interaction scores of scored molecules across five iterations of active learning for c-Abl kinase. The distribution for the model pretrained on the combined dataset with generation conditioned on ADMET filters are shown. Iteration 0 refers to the pretraining phase, while later iterations refer to the active learning phases.

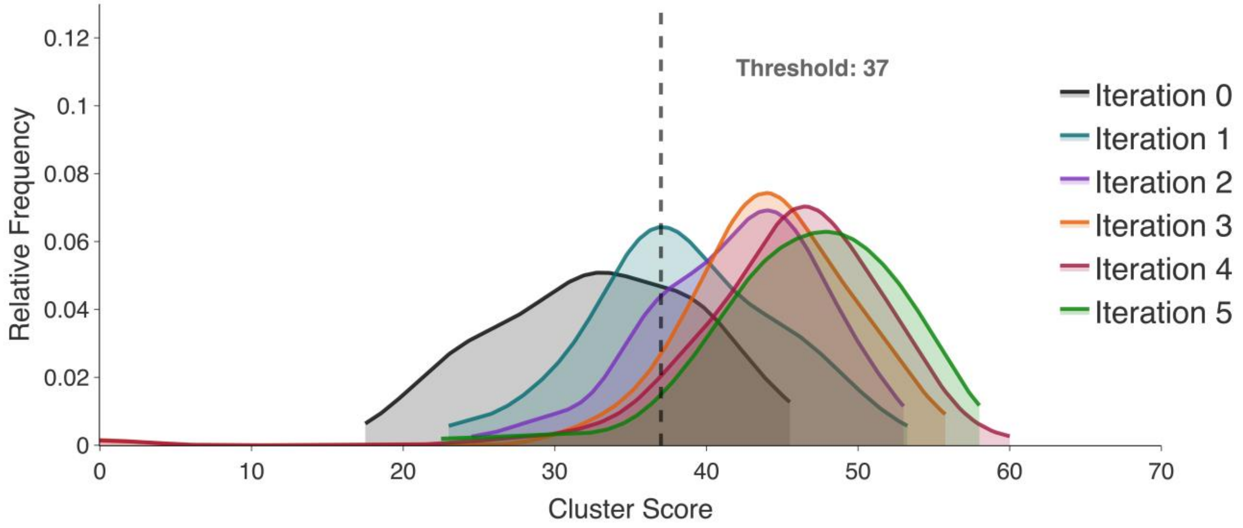

Figure S21.2. Median cluster attractive interaction scores of scored molecules across five iterations of active learning for c-Abl kinase. The distribution for the model pretrained on the combined dataset with generation conditioned on ADMET filters are shown. Iteration 0 refers to the pretraining phase, while later iterations refer to the active learning phases.

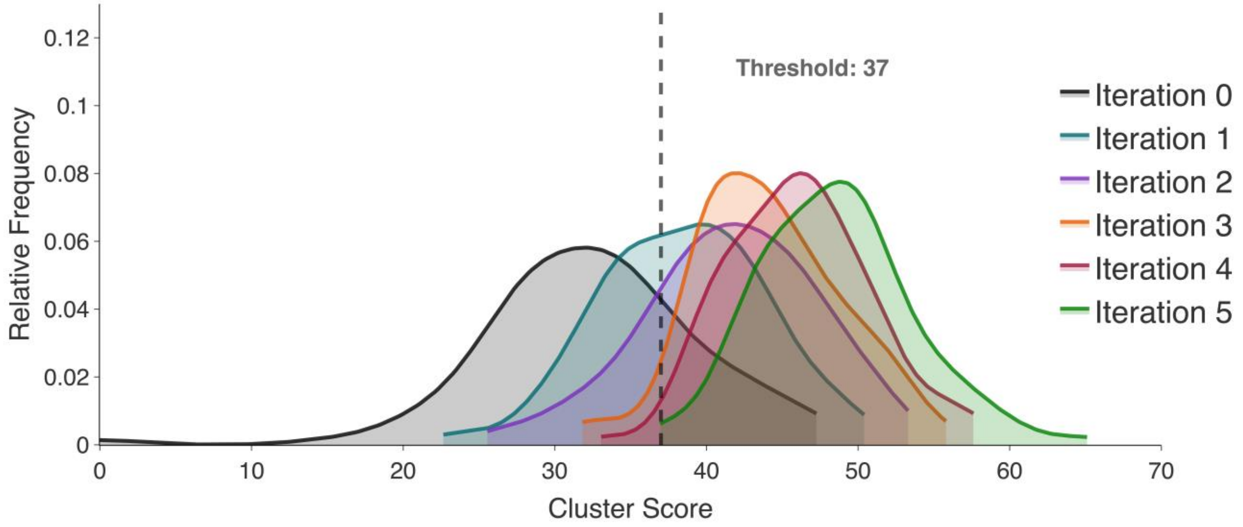

Figure S21.3. Mean cluster attractive interaction scores of scored molecules across five iterations of active learning for c-Abl kinase. The distribution for the model pretrained on the combined dataset with generation conditioned on ADMET and functional group filters are shown. Iteration 0 refers to the pretraining phase, while later iterations refer to the active learning phases.

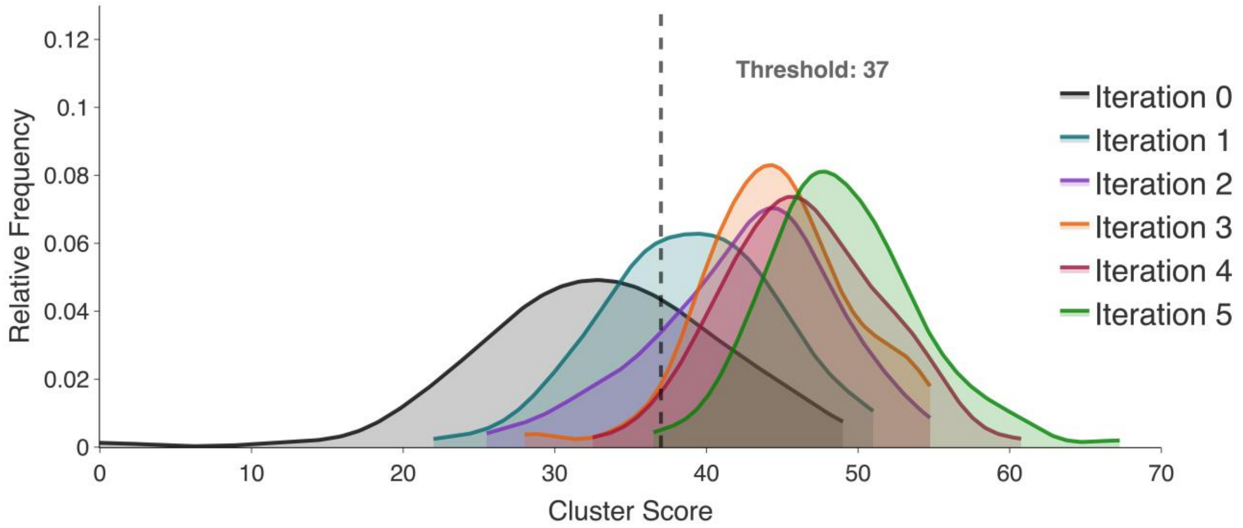

Figure S21.4. Median cluster attractive interaction scores of scored molecules across five iterations of active learning for c-Abl kinase. The distribution for the model pretrained on the combined dataset with generation conditioned on ADMET and functional group filters are shown. Iteration 0 refers to the pretraining phase, while later iterations refer to the active learning phases.

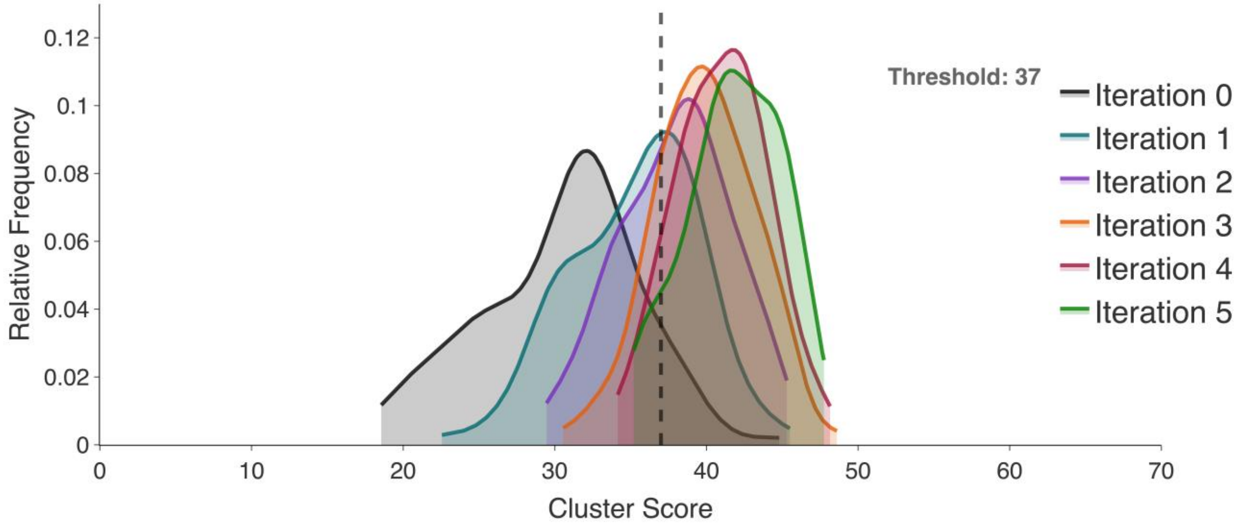

Figure S21.5. Mean cluster attractive interaction scores of scored molecules across five iterations of active learning for c-Abl kinase. The distribution for the model pretrained on the MOSES dataset with generation conditioned on ADMET and functional group filters are shown. Iteration 0 refers to the pretraining phase, while later iterations refer to the active learning phases.

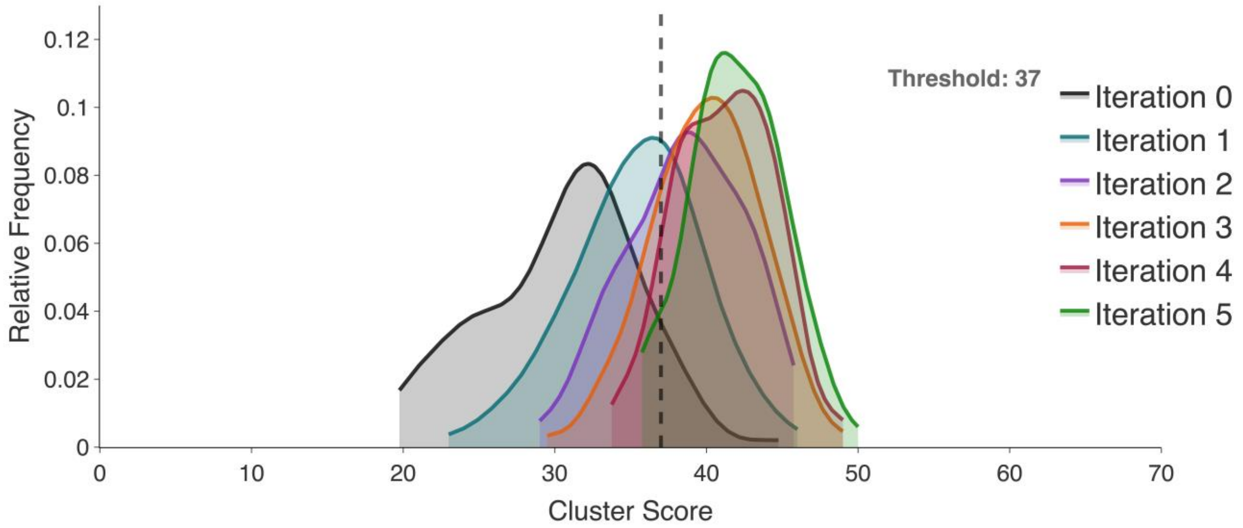

Figure S21.6. Median cluster attractive interaction scores of scored molecules across five iterations of active learning for c-Abl kinase. The distribution for the model pretrained on the MOSES dataset with generation conditioned on ADMET and functional group filters are shown. Iteration 0 refers to the pretraining phase, while later iterations refer to the active learning phases.

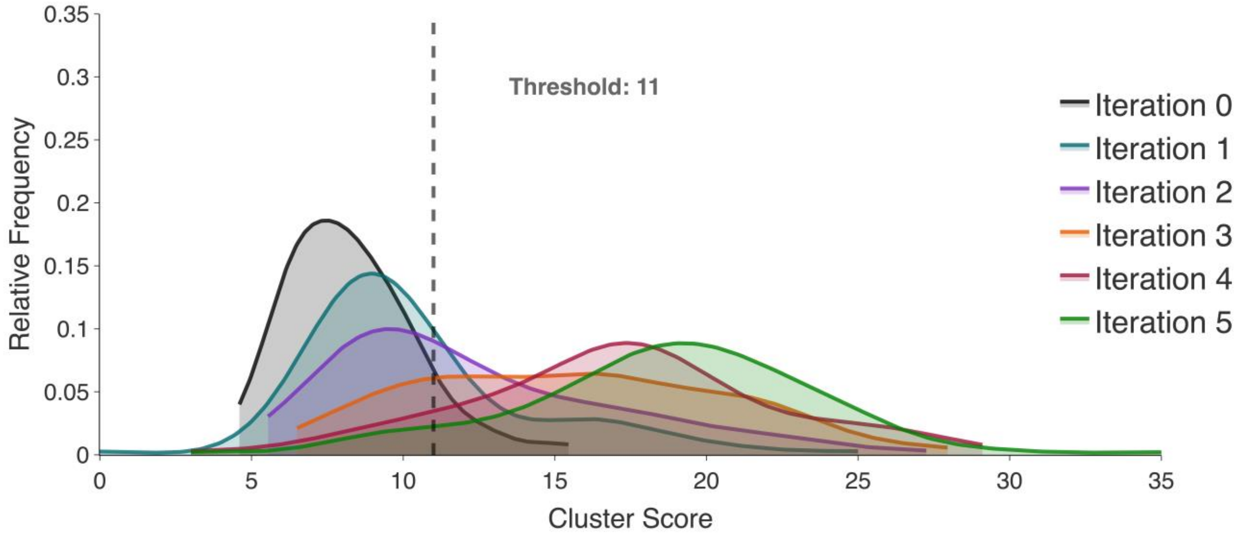

Figure S21.7. Mean cluster attractive interaction scores of scored molecules across five iterations of active learning for the HNH domain of Cas9. The distribution for the model pretrained on the combined dataset with generation conditioned on ADMET filters are shown. Iteration 0 refers to the pretraining phase, while later iterations refer to the active learning phases.

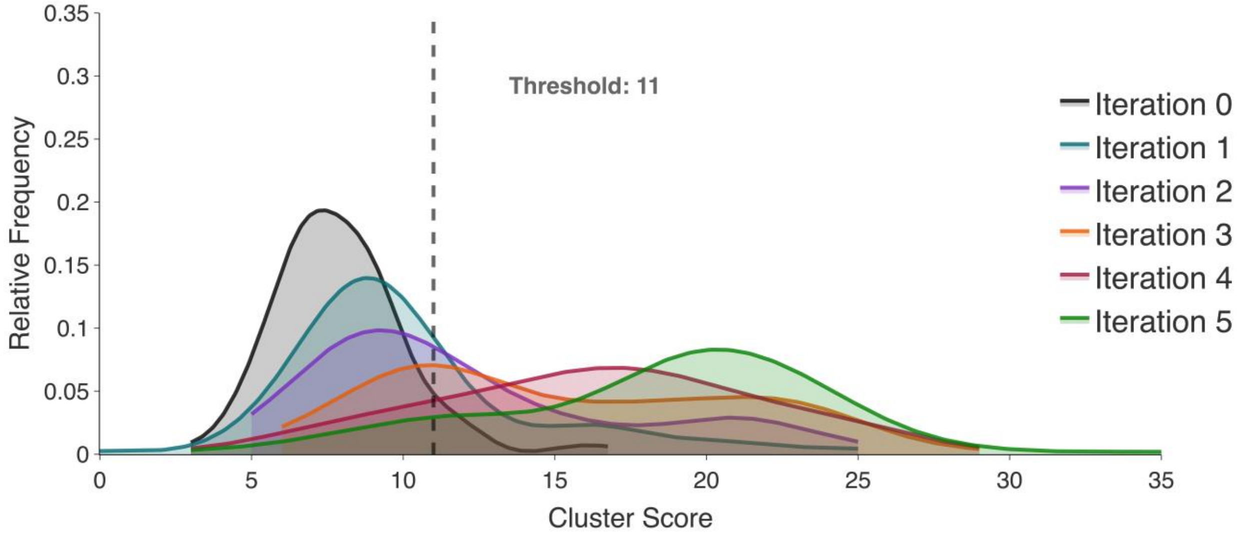

Figure S21.8. Median cluster attractive interaction scores of scored molecules across five iterations of active learning for the HNH domain of Cas9. The distribution for the model pretrained on the combined dataset with generation conditioned on ADMET filters are shown. Iteration 0 refers to the pretraining phase, while later iterations refer to the active learning phases.

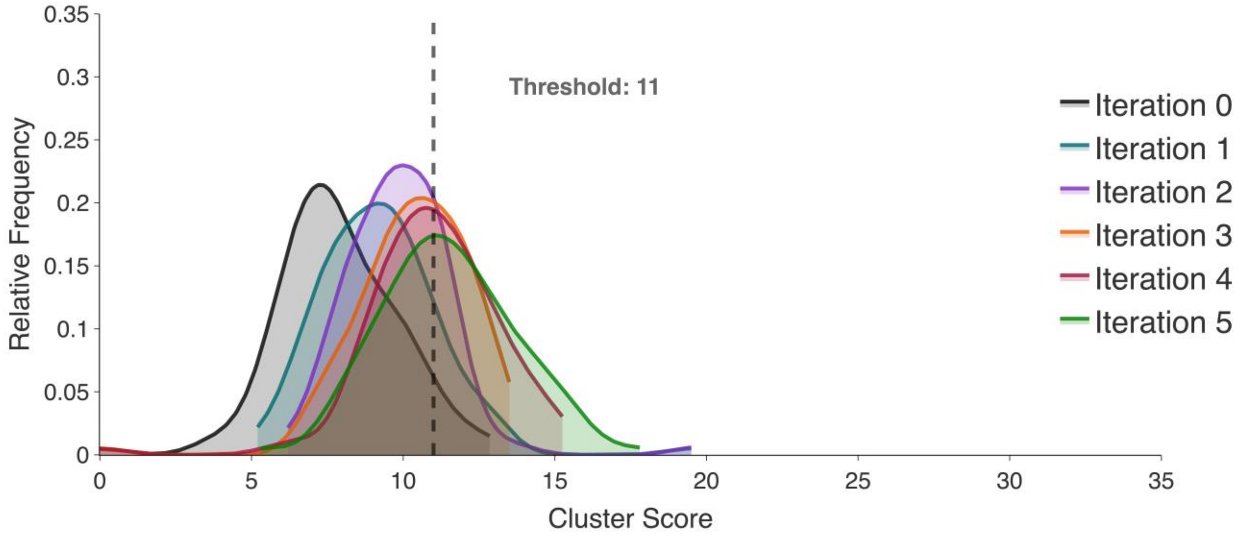

Figure S21.9. Mean cluster attractive interaction scores of scored molecules across five iterations of active learning for the HNH domain of Cas9. The distribution for the model pretrained on the combined dataset with generation conditioned on ADMET and functional group filters are shown. Iteration 0 refers to the pretraining phase, while later iterations refer to the active learning phases.

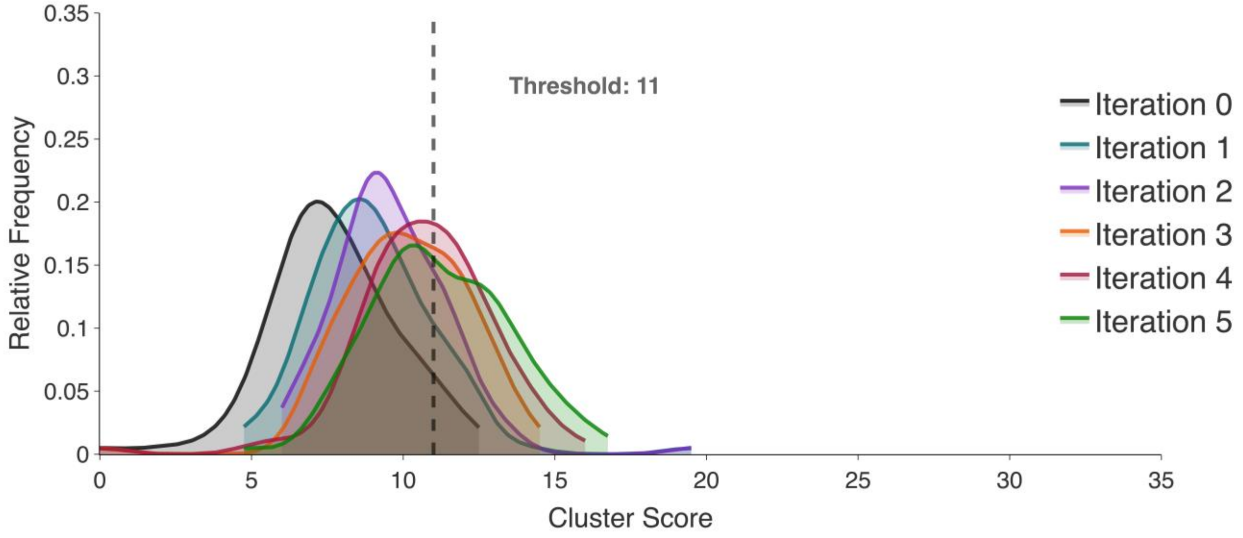

Figure S21.10. Median cluster attractive interaction scores of scored molecules across five iterations of active learning for the HNH domain of Cas9. The distribution for the model pretrained on the combined dataset with generation conditioned on ADMET and functional group filters are shown. Iteration 0 refers to the pretraining phase, while later iterations refer to the active learning phases.

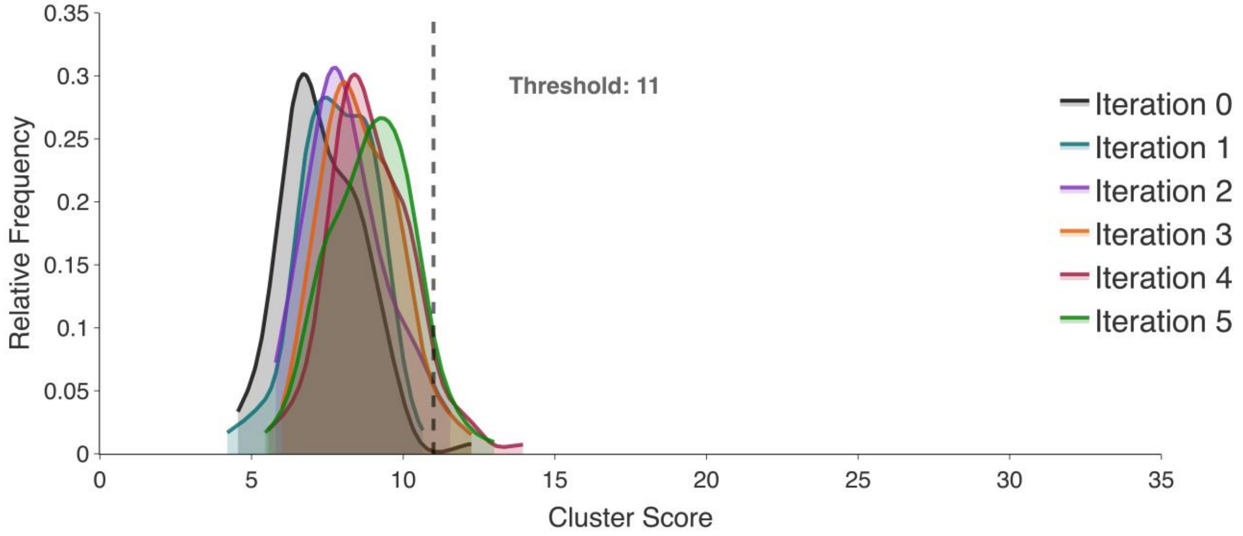

Figure S21.11. Mean cluster attractive interaction scores of scored molecules across five iterations of active learning for the HNH domain of Cas9. The distribution for the model pretrained on the MOSES dataset with generation conditioned on ADMET and functional group filters are shown. Iteration 0 refers to the pretraining phase, while later iterations refer to the active learning phases.

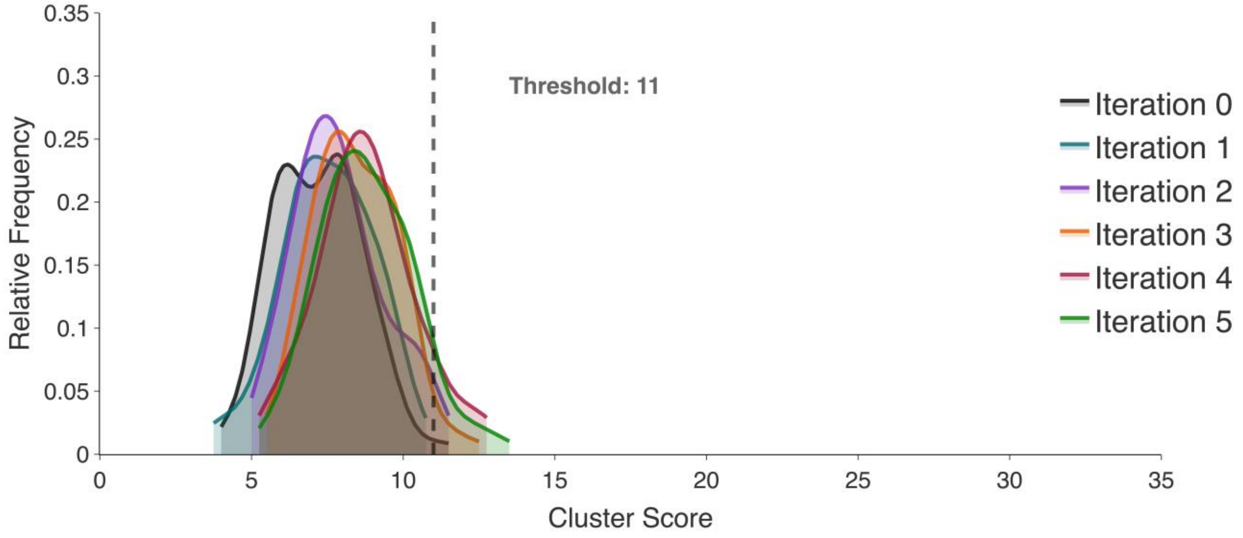

Figure S21.12. Median cluster attractive interaction scores of scored molecules across five iterations of active learning for the HNH domain of Cas9. The distribution for the model pretrained on the MOSES dataset with generation conditioned on ADMET and functional group filters are shown. Iteration 0 refers to the pretraining phase, while later iterations refer to the active learning phases.

<u>Section 22</u>: Additional Evaluation of Generations across Active Learning Iterations.

Results in this section are regarding the model pretrained on the combined dataset with no filters applied to the generations, for alignment to HNH. It should be noted in the comparisons that poor values for the generations from the model aligned with sets curated with random sampling are likely due to memorization, since we only utilize replicas of scored molecules in this scenario.

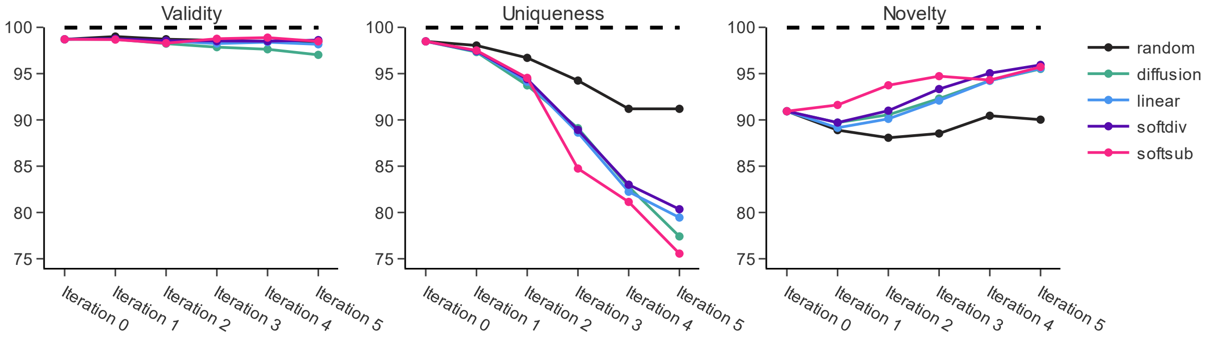

Figure S22.1. Percentage of molecules generated by our model that are valid, unique, or novel after pretraining (iteration 0) and five rounds of active learning. Data are shown for different sampling/conversion schemes.

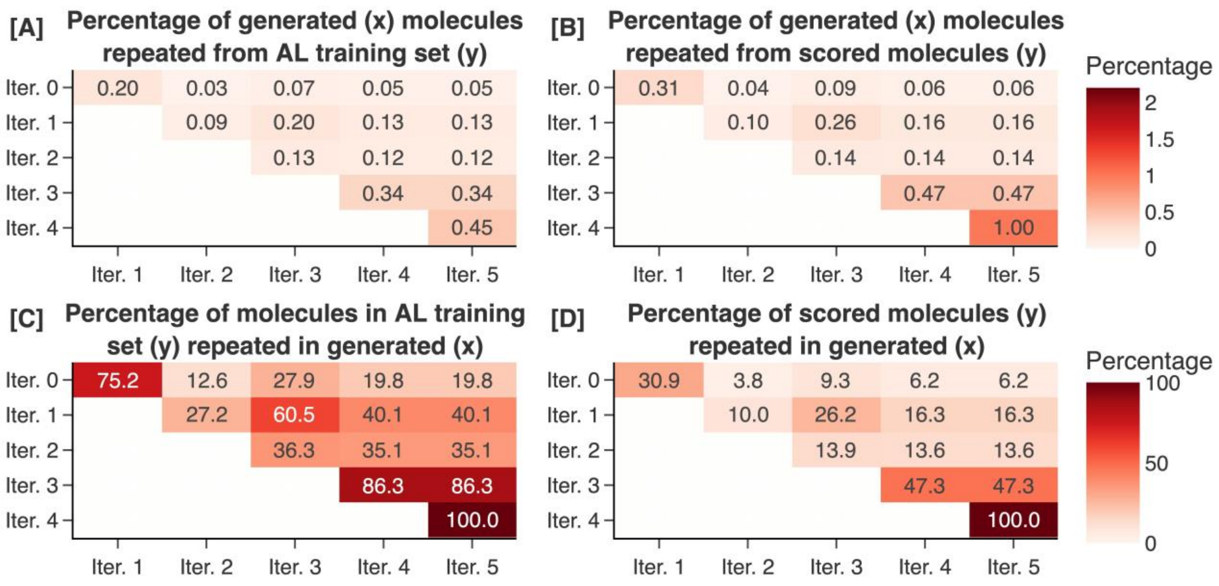

Figure S22.2. Memorization of training set by our model over five rounds of naïve active learning with random selection. (A) The percentage of molecules in a set of 100,000 generated at iteration *i* that occur in the training set at iteration *i*-1. (B) The percentage of molecules in a set of 100,000 generated at iteration *i* that occur in the set of scored molecules at iteration *i*-1. (C) The number of molecules from the active training set at iteration *i*-1 that occurs in generations at iteration *i* divided by the size of the active learning training set at iteration *i*-1 multiplied by 100. (D) the number of scored molecules at iteration *i*-1 that occur in generations at iteration *i* divided by number of scored molecules at iteration *i*-1 (i.e., 1000) multiplied by 100.

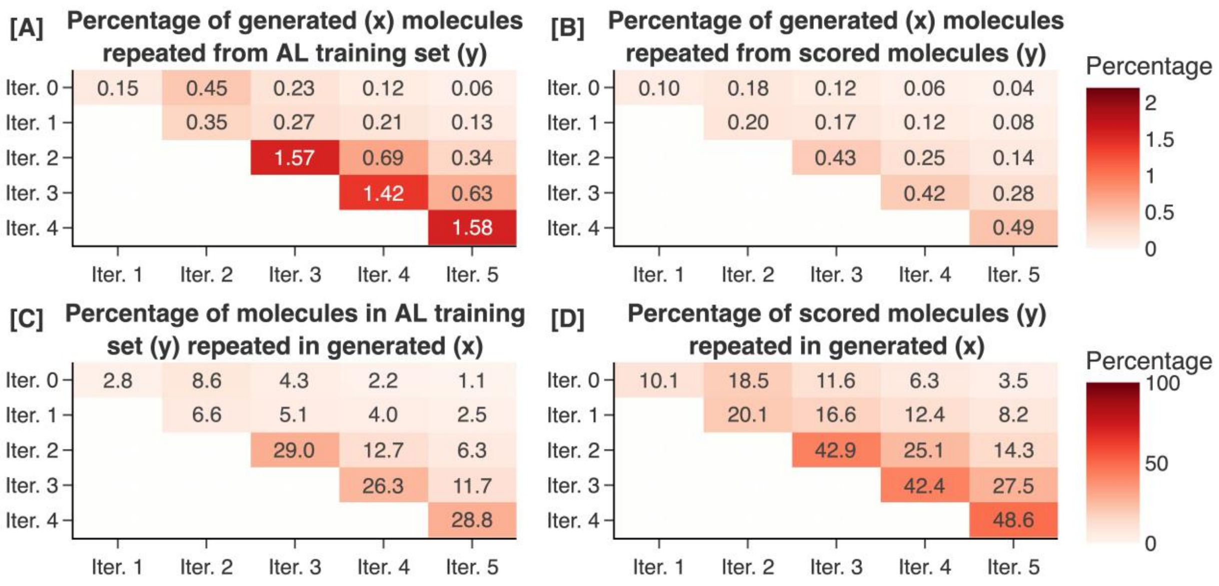

Figure S22.3. Memorization of training set by our model over five rounds of active learning with 100 clusters and uniform selection. (A) The percentage of molecules in a set of 100,000 generated at iteration *I* that occur in the training set at iteration *i*-1. (B) The percentage of molecules in a set of 100,000 generated at iteration *i* that occur in the set of scored molecules at iteration *i*-1. (C) The number of molecules from the active training set at iteration *i*-1 that occurs in generations at iteration *i* divided by the size of the active learning training set at iteration *i*-1 multiplied by 100. (D) the number of scored molecules at iteration *i*-1 that occur in generations at iteration *i* divided by number of scored molecules at iteration *i*-1 (i.e., 1000) multiplied by 100.

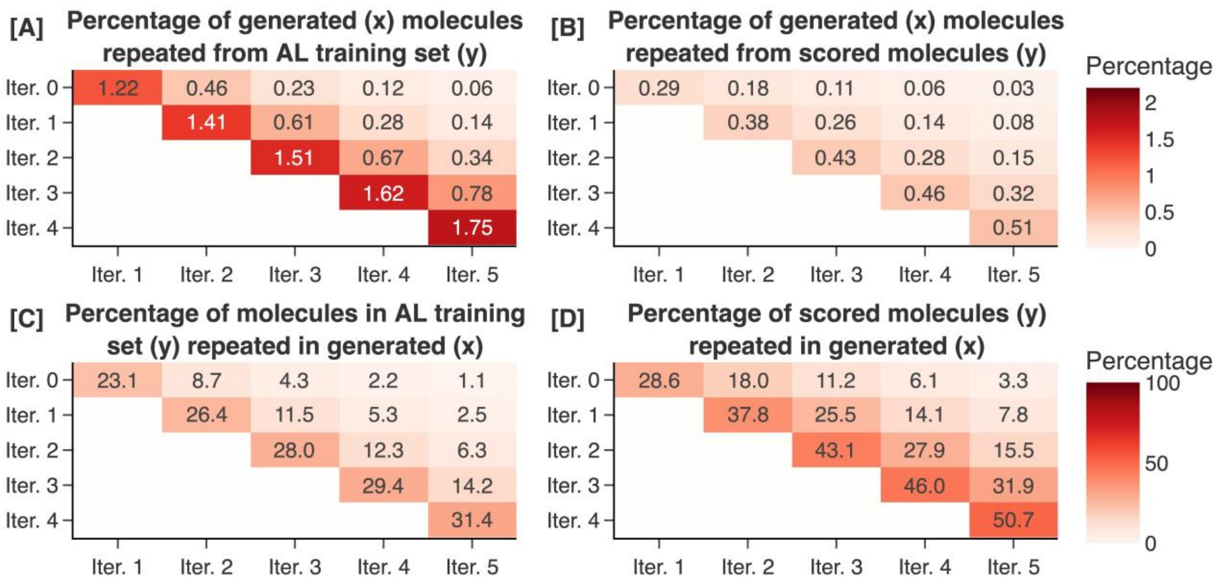

Figure S22.4. Memorization of training set by our model over five rounds of active learning with 100 clusters and linear selection. (A) The percentage of molecules in a set of 100,000 generated at iteration *i* that occur in the training set at iteration *i*-1. (B) The percentage of molecules in a set of 100,000 generated at iteration *i* that occur in the set of scored molecules at iteration *i*-1. (C) The number of molecules from the active training set at iteration *i*-1 that occurs in generations at iteration *i* divided by the size of the active learning training set at iteration *i*-1 multiplied by 100. (D) the number of scored molecules at iteration *i*-1 that occur in generations at iteration *i* divided by number of scored molecules at iteration *i*-1 (i.e., 1000) multiplied by 100.

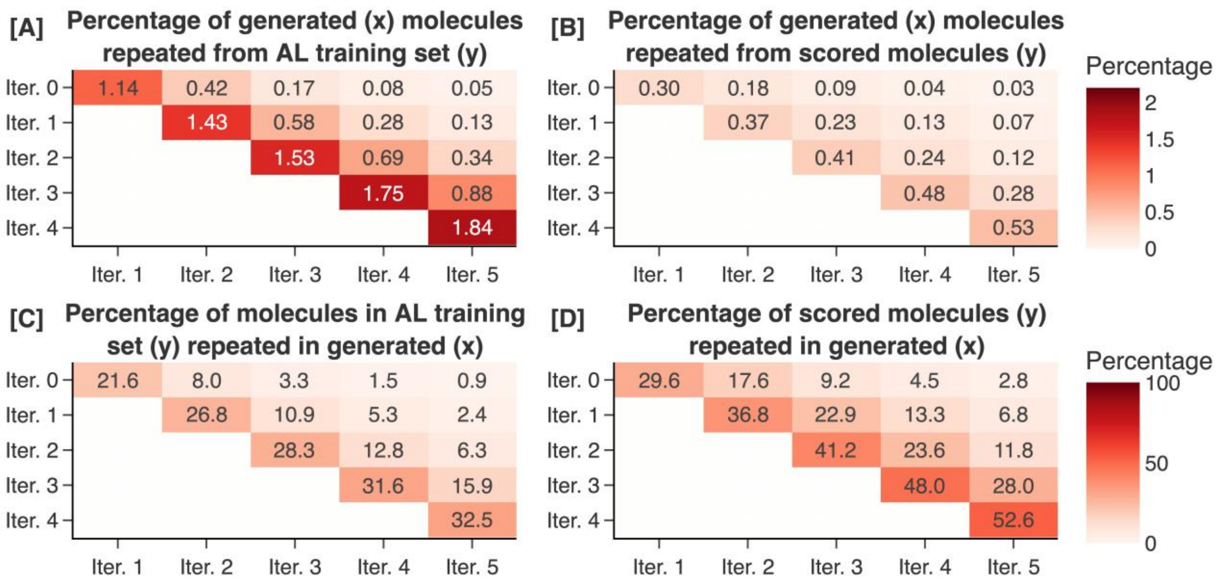

Figure S22.5. Memorization of training set by our model over five rounds of active learning with 100 clusters and softdiv selection. (A) The percentage of molecules in a set of 100,000 generated at iteration *i* that occur in the training set at iteration *i*-1. (B) The percentage of molecules in a set of 100,000 generated at iteration *i* that occur in the set of scored molecules at iteration *i*-1. (C) The number of molecules from the active training set at iteration *i*-1 that occurs in generations at iteration *i* divided by the size of the active learning training set at iteration *i*-1 multiplied by 100. (D) the number of scored molecules at iteration *i*-1 that occur in generations at iteration *i* divided by number of scored molecules at iteration *i*-1 (i.e., 1000) multiplied by 100.

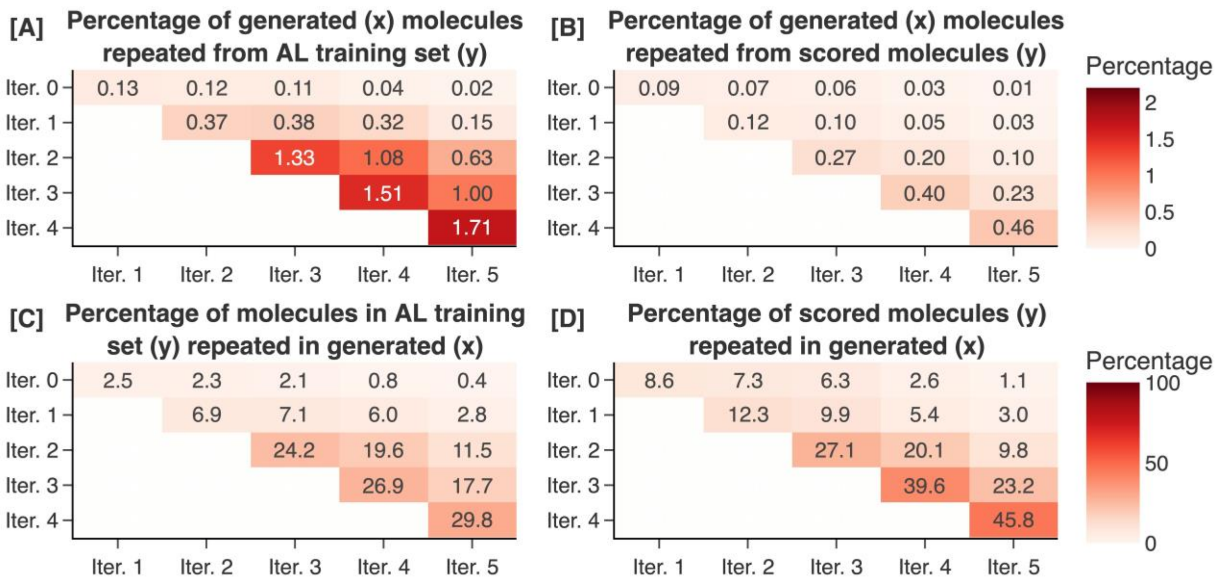

Figure S22.6. Memorization of training set by our model over five rounds of active learning with 100 clusters and softsub selection. (A) The percentage of molecules in a set of 100,000 generated at iteration *i* that occur in the training set at iteration *i*-1. (B) The percentage of molecules in a set of 100,000 generated at iteration *i* that occur in the set of scored molecules at iteration *i*-1. (C) The number of molecules from the active training set at iteration *i*-1 that occurs in generations at iteration *i* divided by the size of the active learning training set at iteration *i*-1 multiplied by 100. (D) the number of scored molecules at iteration *i*-1 that occur in generations at iteration *i* divided by number of scored molecules at iteration *i*-1 (i.e., 1000) multiplied by 100.

References

[1] ADMETlab 2.0. URL: https://admetmesh.scbdd.com/explanation/index.

[2] Bagal, V.; Aggarwal, R.; Vinod, P. K.; Priyakumar, U. D. MolGPT: Molecular Generation Using a Transformer-Decoder Model. *J. Chem. Inf. Model.* **2022**, *62*, 2064-2076. DOI: 10.1021/acs.jcim.1c00600.

[3] Prykhodko, O.; Johansson, S. V.; Kotsias, P.-C.; Arús-Pous, J.; Bjerrum, E. J.; Engkvist, O.; Chen, H. A de novo molecular generation method using latent vector based generative adversarial network. *J. Cheminf.* **2019**, *11*, 74. DOI: 10.1186/s13321-019-0397-9.

[4] Jin, W.; Barzilay, R.; Jaakkola, T. Junction Tree Variational Autoencoder for Molecular Graph Generation. In Proceedings of the 35th International Conference on Machine Learning, Proceedings of Machine Learning Research, **2018**.

[5] Segler, M. H. S.; Kogej, T.; Tyrchan, C.; Waller, M. P. Generating Focused Molecule Libraries for Drug Discovery with Recurrent Neural Networks. *ACS Cent. Sci.* **2018**, *4*, 120-131. DOI: 10.1021/acscentsci.7b00512.

[6] Popova, M.; Shvets, M.; Oliva, J.; Isayev, O. MolecularRNN: Generating Realistic Molecular Graphs With Optimized Properties. *ArXiv* **2019**. DOI: https://doi.org/10.48550/arXiv.1905.13372.

[7] Wang, J.; Chu, Y.; Mao, J.; Jeon, H.-N.; Jin, H.; Zeb, A.; Jang, Y.; Cho, K.-H.; Song, T.; No, K. T. De Novo Molecular Design With Deep Molecular Generative Models for PPI inhibitors. *Briefings Bioinf.* **2022**, *23*, bbac285. DOI: 10.1093/bib/bbac285.

[8] Song, T.; Ren, Y.; Wang, S.; Han, P.; Wang, L.; Li, X.; Rodriguez-Patón, A. DNMG: Deep Molecular Generative Model by Fusion of 3D Information for De Novo Drug Design. *Methods* **2023**, *211*, 10-22. DOI: https://doi.org/10.1016/j.ymeth.2023.02.001.

[9] Chenthamarakshan, V.; Das, P.; Hoffman, S. C.; Strobelt, H.; Padhi, I.; Lim, K. W.; Hoover, B.; Manica, M.; Born, J.; Laino, T.; Mojsilovic, A. CogMol: Target-Specific and Selective Drug Design for COVID-19 Using Deep Generative Models. In Proceedings of the 34th International Conference on Neural Information Processing Systems, **2020**.

[10] Dollar, O.; Joshi, N.; Beck, D. A. C.; Pfaendtner, J. Attention-Based Generative Models for De Novo Molecular Design. *Chem. Sci.* **2021**, *12*, 8362-8372. DOI: 10.1039/D1SC01050F.

[11] Skalic, M.; Jiménez, J.; Sabbadin, D.; De Fabritiis, G. Shape-Based Generative Modeling for de Novo Drug Design. *J. Chem. Inf. Model.* **2019**, *59*, 1205-1214. DOI: 10.1021/acs.jcim.8b00706.

[12] Zhavoronkov, A.; Ivanenkov, Y. A.; Aliper, A.; Veselov, M. S.; Aladinskiy, V. A.; Aladinskaya, A. V.; Terentiev, V. A.; Polykovskiy, D. A.; Kuznetsov, M. D.; Asadulaev, A.; Volkov, Y.; Zholus, A.; Shayakhmetov, R. R.; Zhebrak, A.; Minaeva, L. I.; Zagribelnyy, B. A.; Lee, L.H.; Soll, R.; Madge, D.; Xing, L.; Guo, T.; Aspuru-Guzik, A. Deep Learning Enables Rapid Identification of Potent DDR1 Kinase Inhibitors. *Nat. Biotechnol.* **2019**, *37*, 1038-1040. DOI: 10.1038/s41587-019-0224-x.

[13] Mao, J.; Wang, J.; Zeb, A.; Cho, K.-H.; Jin, H.; Kim, J.; Lee, O.; Wang, Y.; No, K. T. Transformer-Based Molecular Generative Model for Antiviral Drug Design. *J. Chem. Inf. Model.* **2023**. DOI: 10.1021/acs.jcim.3c00536.

[14] Wang, J.; Mao, J.; Wang, M.; Le, X.; Wang, Y. Explore drug-like space with deep generative models. *Methods* **2023**, *210*, 52-59. DOI: https://doi.org/10.1016/j.ymeth.2023.01.004.

[15] Kim, H.; Na, J.; Lee, W. B. Generative Chemical Transformer: Neural Machine Learning of Molecular Geometric Structures from Chemical Language via Attention. *J. Chem. Inf. Model.* **2021**, *61*, 5804-5814. DOI: 10.1021/acs.jcim.1c01289.

[16] Wang, Y.; Zhao, H.; Sciabola, S.; Wang, W. cMolGPT: A Conditional Generative Pre-Trained Transformer for Target-Specific De Novo Molecular Generation. *Molecules* **2023**, *28*. DOI: 10.3390/molecules28114430.

[17] Mercado, R.; Rastemo, T.; Lindelöf, E.; Klambauer, G.; Engkvist, O.; Chen, H.; Bjerrum, E. J. Graph Networks for Molecular Design. *ChemRxiv* **2020**. DOI: 10.26434/chemrxiv.12843137.v1.

[18] Wang, W.; Wang, Y.; Zhao, H.; Sciabola, S. A Transformer-Based Generative Model for De Novo Molecular Design. *ArXiv* **2022**, DOI: https://doi.org/10.48550/arXiv.2210.08749.

[19] Wei, L.; Fu, N.; Song, Y.; Wang, Q.; Hu, J. Probabilistic Generative Transformer Language Models for Generative Design of Molecules. *ArXiv* **2022**, DOI: https://doi.org/10.48550/arXiv.2209.09406.

[20] Polykovskiy, D.; Zhebrak, A.; Sanchez-Lengeling, B.; Golovanov, S.; Tatanov, O.; Belyaev, S.; Kurbanov, R.; Artamonov, A.; Aladinskiy, V.; Veselov, M.; Kadurin, A.; Johansson, S.; Chen, H.; Nikolenko, S.; Aspuru-Guzik, A.; Zhavoronkov, A. Molecular Sets (MOSES): A Benchmarking Platform for Molecular Generation Models. *Front. Pharmacol.* **2020**, *11*, 565644. DOI: 10.3389/fphar.2020.565644.

[21] Pedregosa, F.; Varoquaux, G.; Gramfort, A.; Michel, V. ; Thirion, B. ; Grisel, O, ; Blondel, M. ; Prettenhofer, P. ; Weiss, R. ; Dubourg, V.; Vanderplas, J.; Passos, A.; Cournapeau, D.; Brucher, M.; Perrot, M.; Duchesnay, E. Scikit-learn: Machine Learning in Python. *J. Mach. Learn. Res.* **2011**, *12*, 2825-2830. DOI: http://jmlr.org/papers/v12/pedregosa11a.html.

For Table of Contents Use Only

ChemSpaceAL: An Efficient Active Learning Methodology Applied to Protein-Specific Molecular Generation

Gregory W. Kyro, Anton Morgunov, Rafael I. Brent, Victor S. Batista*

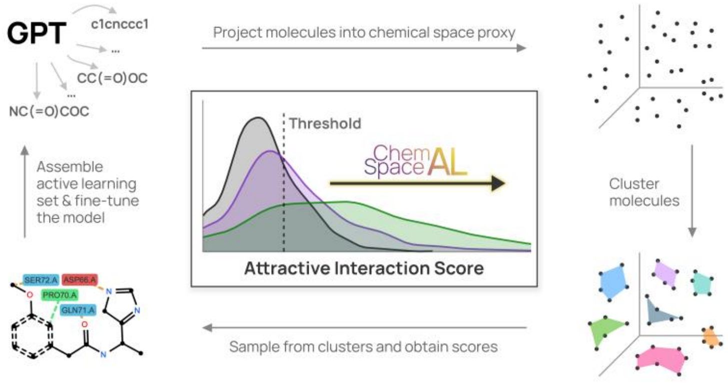

Supplement: 1 [file NIHPP2309.05853V2-supplement-1.pdf]
