# Supplementary material for: ChemSpaceAL: An Efficient Active Learning Methodology Applied to Protein-Specific Molecular Generation
Source: ArXiv. 2023 Sep 11:arXiv:2309.05853v1. Preprint. [Version 1] (PMC10516108)
Supplement: Supplementary file 1 [file NIHPP2309.05853V1-supplement-1.pdf]

Supporting Information

List of Figures and Tables

Section 1: Training our generative model

- **1.1:** Training loss, validation loss and learning rate during the 30 epochs of pretraining of our model on the combined dataset.
- **1.2:** Training step losses (evaluated after each batch) during 5 rounds of active learning (10 epochs each) with different conversion methods.

Section 2: Construction of the chemical space proxy from RDKit descriptors

- **2.1:** List of RDKit descriptors used for the construction of the chemical space proxy.
- **2.2:** List of RDKit descriptors excluded prior to performing Principal Component Analysis.
- **2.3:** Cumulative fraction of variance explained by the first *N* principal components.

Section 3: Alternative methods for converting mean cluster scores to sampling fractions

- **3.1:** Distribution of sampling fractions obtained with different conversion methods applied to cluster scores obtained from generations of the pretrained model.

Section 4: Evolution of the distribution of attractive interaction scores for different conversion methods

- **4.1:** Attractive interaction scores for molecules generated by the pretrained model (iteration 0) and by the model after each of the five iterations of active learning where, prior to sampling for docking, molecules in the chemical space are grouped into 100 clusters, and cluster scores are converted into sampling fractions using the *linear* method.
- **4.2:** Attractive interaction scores for molecules generated by the pretrained model (iteration 0) and by the model across each of the five iterations of active learning where, prior to sampling for docking, molecules in the chemical space are grouped into 100 clusters, and cluster scores are converted into sampling fractions using the *softdiv* method.
- **4.3:** Statistics of the distribution of attractive interaction scores, when molecules are selected *randomly* (naïve active learning), with no clustering.
- **4.4:** Statistics of the distribution of attractive interaction scores, when molecules are clustered into 100 groups and cluster scores are sampled *uniformly*.
- **4.5:** Statistics of the distribution of attractive interaction scores, when molecules are clustered into 100 groups and cluster scores are converted into sampling fractions using the *linear* method.
- **4.6:** Statistics of the distribution of attractive interaction scores, when molecules are clustered into 100 groups and cluster scores are converted into sampling fractions using the *softdiv* method.

Section 5: Evolution of the distribution of the number of attractive interactions for different conversion methods

- **5.1:** Counts of interactions of each type for 1000 scored molecules generated by the pretrained model (iteration 0) and by the model after each of the five rounds of naïve active learning with *random* sampling.
- **5.2:** Counts of interactions of each type for 1000 molecules generated by the pretrained model (iteration 0) and by the model after each of the five rounds of active learning with clustering into 100 groups and *uniform* selection from each cluster.
- **5.3:** Counts of interactions of each type for 1000 scored molecules generated by the pretrained model (iteration 0) and by the model after each of the five rounds of active learning with clustering into 100 groups and conversion of cluster scores into sampling fractions using the *linear* method.
- **5.4:** Counts of interactions of each type for 1000 scored molecules generated by the pretrained model (iteration 0) and by the model after each of the five rounds of active learning with clustering into 100 groups and conversion of cluster scores into sampling fractions using the *softdiv* method.
- **5.5:** Counts of interactions of each type for 1000 molecules generated by the pretrained model (iteration 0) and by the model after each of the five rounds of active learning with clustering into 100 groups and conversion of cluster scores into sampling fractions using the *softsub* method.

Section 6: Evolution of the distribution of cluster-average attractive interaction scores for different conversion methods

- **6.1:** Cluster scores (obtained as an average of attractive interaction scores for molecules in the cluster) for molecules generated by the pretrained model (iteration 0) and by the model after each of the five iterations of active learning where, prior to sampling for docking, molecules in the chemical space are grouped into 100 clusters, and molecules are sampled from each cluster *uniformly*.
- **6.2:** Cluster scores (obtained as an average of attractive interaction scores for molecules in the cluster) for molecules generated by the pretrained model (iteration 0) and by the model after each of the five iterations of active learning where, prior to sampling for docking, molecules in the chemical space are grouped into 100 clusters, and molecules are sampled from each cluster using the *linear* method.
- **6.3:** Cluster scores (obtained as an average of attractive interaction scores for molecules in the cluster) for molecules generated by the pretrained model (iteration 0) and by the model after each of the five iterations of active learning where, prior to sampling for docking, molecules in the chemical space are grouped into 100 clusters, and molecules are sampled from each cluster using the *softdiv* method.
- **6.4:** Cluster scores (obtained as an average of attractive interaction scores for molecules in the cluster) for molecules generated by the pretrained model (iteration 0) and by the model after each of the five iterations of active learning where, prior to sampling for docking, molecules in the chemical space are grouped into 100 clusters, and molecules are sampled from each cluster using the *softsub* method.
- **6.5:** Cluster scores (obtained as an average of attractive interaction scores for molecules in the cluster) for molecules generated by the pretrained model (iteration 0) and by the model after each of the five iterations of active learning where, prior to sampling for docking,

molecules in the chemical space are grouped into 10 clusters, and molecules are sampled from each cluster using the *softsub* method.

Section 7: Performance on the MOSES benchmark

- **7.1:** Primary results of our pretrained model on the MOSES benchmark compared to top-performing models in the field.
- **7.2:** Additional results of our pretrained model on the MOSES benchmark compared to top-performing models in the field.

Section 8: Details on the execution of t-distributed stochastic neighbor embedding (t-SNE)

Section 9: Vocabulary composition of the combined dataset

- **9.1:** List of unique tokens that occur in the raw combined dataset less than 1,000 times.
- **9.2:** List of unique tokens in the processed combined dataset.

Section 10: Choosing the number of clusters to use for k-means

- **10.1:** Attractive interaction scores for molecules generated by the pretrained model (iteration 0) and by the model after each of the five iterations of active learning where, prior to sampling for docking, molecules in the chemical space are grouped into 10 clusters, and cluster scores are converted into sampling fractions using the softsub approach.
- **10.2:** Attractive interaction scores for molecules generated by the pretrained model (iteration 0) and by the model after each of the five iterations of active learning where, prior to sampling for docking, molecules in the chemical space are grouped into 100 clusters, and cluster scores are converted into sampling fractions using the softsub approach.
- **10.3:** Statistics of the distribution of attractive interaction scores, when molecules are clustered into 10 groups and cluster scores are converted into sampling fractions using the softsub method.
- **10.4:** Statistics of the distribution of attractive interaction scores, when molecules are clustered into 100 groups and cluster scores are converted into sampling fractions using the softsub method.

Section 11: Additional evaluation of generations across active learning iterations

- **11.1.** Percentage of molecules generated by our model that are valid, unique, or novel after pretraining (iteration 0) and five rounds of active learning.
- **11.2.** Memorization of training sets by our model over five rounds of naïve active learning with random selection.
- **11.3.** Memorization of training sets by our model over five rounds of active learning with 100 clusters and *uniform* selection.
- **11.4.** Memorization of training sets by our model over five rounds of active learning with 100 clusters and *linear* selection.
- **11.5.** Memorization of training sets by our model over five rounds of active learning with 100 clusters and *softdiv* selection.
- **11.6.** Memorization of training sets by our model over five rounds of active learning with 100 clusters and *softsub* selection.

Section 1: Training our generative model

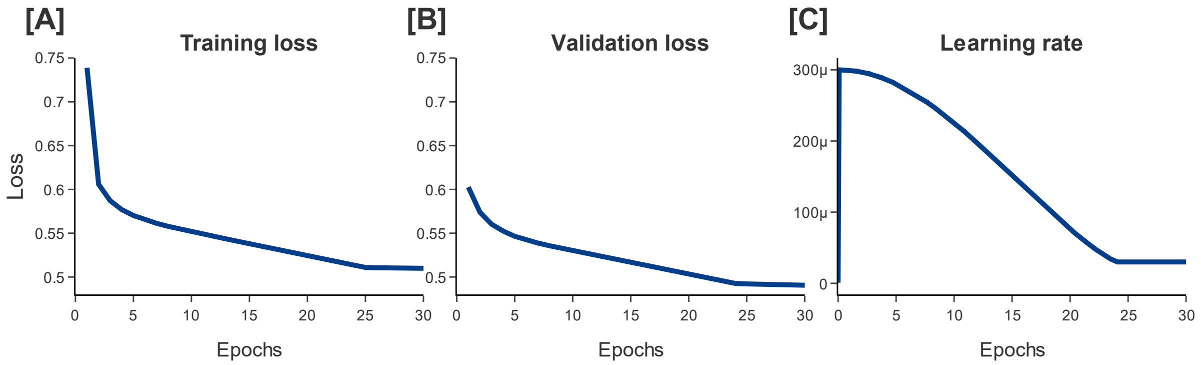

Figure S1.1. Training loss (A), validation loss (B), and learning rate (C) during the 30 epochs of pretraining of our model on the combined dataset.

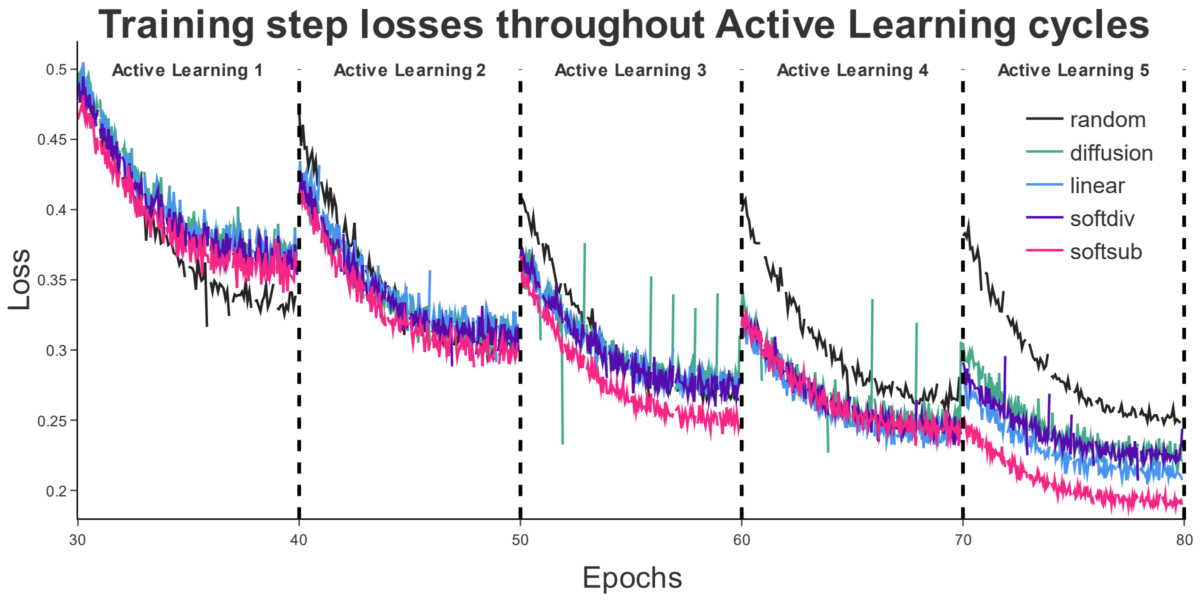

Figure S1.2. Training step losses (evaluated after each batch) during 5 rounds of active learning (10 epochs each) with different conversion methods.

Section 2: Construction of the chemical space proxy from RDKit descriptors

Table S2.1. List of RDKit descriptors used for the construction of the chemical space proxy.

- AvgIpc
- BalabanJ
- BertzCT
- Chi0
- Chi0n
- Chi0v
- Chi1
- Chi1n
- Chi1v
- Chi2n
- Chi2v
- Chi3n
- Chi3v
- Chi4n
- Chi4v
- EState_VSA1
- EState_VSA10
- EState_VSA11
- EState_VSA2
- EState_VSA3
- EState_VSA4
- EState_VSA5
- EState_VSA6
- EState_VSA7
- EState_VSA8
- EState_VSA9
- ExactMolWt
- FpDensityMorgan1
- FpDensityMorgan2
- FpDensityMorgan3
- FractionCSP3
- HallKierAlpha
- HeavyAtomCount
- HeavyAtomMolWt
- Kappa1
- Kappa2
- Kappa3
- LabuteASA
- MaxAbsEState Index
- MaxEStateIndex
- MinAbsEState Index
- MinEStateIndex
- MolLogP
- MolMR
- MolWt
- NHOHCount
- NOCount
- NumAliphatic Carbocycles
- NumAliphatic Heterocycles
- NumAliphatic Rings
- NumAromatic Carbocycles
- NumAromatic Heterocycles
- NumAromatic Rings
- NumHAcceptors
- NumHDonors
- NumHeteroatoms
- NumRadical Electrons
- NumRotatable Bonds
- NumSaturated Carbocycles
- NumSaturated Heterocycles
- NumSaturated Rings
- NumValence Electrons
- PEOE_VSA1
- PEOE_VSA10
- PEOE_VSA11
- PEOE_VSA12
- PEOE_VSA13
- PEOE_VSA14
- PEOE_VSA2
- PEOE_VSA3
- PEOE_VSA4
- PEOE_VSA5
- PEOE_VSA6
- PEOE_VSA7
- PEOE_VSA8
- PEOE_VSA9
- RingCount
- SMR_VSA1
- SMR_VSA10
- SMR_VSA2
- SMR_VSA3
- SMR_VSA4
- SMR_VSA5
- SMR_VSA6
- SMR_VSA7
- SMR_VSA8
- SMR_VSA9
- SlogP_VSA1
- SlogP_VSA10
- SlogP_VSA11
- SlogP_VSA12
- SlogP_VSA2
- SlogP_VSA3
- SlogP_VSA4
- SlogP_VSA5
- SlogP_VSA6
- SlogP_VSA7
- SlogP_VSA8
- SlogP_VSA9
- TPSA
- VSA_EState1
- VSA_EState10
- VSA_EState2
- VSA_EState3
- VSA_EState4
- VSA_EState5
- VSA_EState6
- VSA_EState7
- VSA_EState8
- VSA_EState9
- fr_Al_COO

- fr_Al_OH
- fr_Al_OH_noTert
- fr_ArN
- fr_Ar_COO
- fr_Ar_N
- fr_Ar_NH
- fr_Ar_OH
- fr_COO
- fr_COO2
- fr_C_O
- fr_C_O_noCOO
- fr_C_S
- fr_HOCCN
- fr_Imine
- fr_NH0
- fr_NH1
- fr_NH2
- fr_N_O
- fr_Ndealkylation1
- fr_Ndealkylation2
- fr_Nhpyrrole
- fr_SH
- fr_aldehyde
- fr_alkyl_carbamate
- fr_alkyl_halide
- fr_allylic_oxid
- fr_amide
- fr_amidine
- fr_aniline
- fr_aryl_methyl
- fr_azide
- fr_azo
- fr_barbitur
- fr_benzene
- fr_benzodiazepine
- fr_bicyclic
- fr_diazo
- fr_dihydropyridine
- fr_epoxide
- fr_ester
- fr_ether
- fr_furan
- fr_guanido
- fr_halogen
- fr_hdrzine
- fr_hdrzone
- fr_imidazole
- fr_imide
- fr_isocyan
- fr_isothiocyan
- fr_ketone
- fr_ketone_Topliss
- fr_lactam
- fr_lactone
- fr_methoxy
- fr_morpholine
- fr_nitrile
- fr_nitro
- fr_nitro_arom
- fr_nitro_arom_ nonortho
- fr_nitroso
- fr_oxazole
- fr_oxime
- fr_para_ hydroxylation
- fr_phenol
- fr_phenol_ noOrthoHbond
- fr_phos_acid
- fr_phos_ester
- fr_piperdine
- fr_piperzine
- fr_priamide
- fr_prisulfonamd
- fr_pyridine
- fr_quatN
- fr_sulfide
- fr_sulfonamd
- fr_sulfone
- fr_term_acetylene
- fr_tetrazole
- fr_thiazole
- fr_thiocyan
- fr_thiophene
- fr_unbrch_alkane
- fr_urea
- qed

Table S2.2. List of RDKit descriptors excluded prior to performing Principal Component Analysis.

- BCUT2D_CHGHI
- BCUT2D_CHGLO
- BCUT2D_LOGPHI
- BCUT2D_LOGPLOW
- BCUT2D_MRHI
- BCUT2D_MRLOW
- BCUT2D_MWHI
- BCUT2D_MWLOW
- Ipc
- MaxAbsPartialCharge
- MaxPartialCharge
- MinAbsPartialCharge
- MinPartialCharge

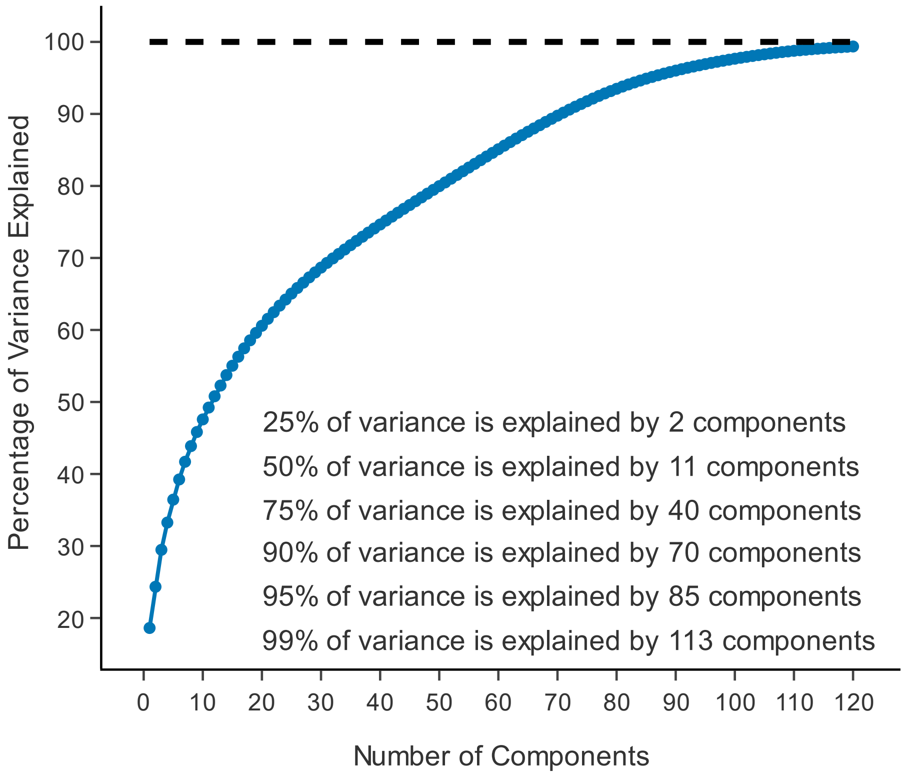

Figure S2.3. Cumulative fraction of variance explained by the first *N* principal components. Our chemical space proxy (i.e., the first 120 principal components) explains 99.3% of the variance in the hyperspace of 196 RDKit descriptors.

Section 3: Alternative methods for converting mean cluster scores to sampling fractions

Prior to constructing the active learning training set, we need to convert the attractive interaction scores s_i obtained by using the prolif software on docked molecules into sampling fractions f_i, which will be used to calculate the number of molecules that we need to sample from each cluster. A simple way to do that is to normalize the sum of all scores to unity:

$$f_i^{\text{linear}}(s_i) = \frac{s_i}{\sum_i s_i}$$

We call this approach *linear* conversion. Because one could interpret sampling fractions as effective probabilities of sampling from a given cluster, it is natural to consider the use of a softmax function:

$$f_i^{\text{softmax}}(s_i) = \frac{e^{s_i}}{\sum_i e^{s_i}}$$

which, for computational stability purposes, is often implemented with the maximum value among a set of arguments subtracted from each individual argument. To contrast with a modification of a softmax function introduced later, we refer to this as *softsub* conversion. In the main text of our paper, we implement the *softsub* approach and refer to it as *softmax* because this is the common implementation of the softmax function.

$$f_i^{\text{softsub}}(s_i) = \frac{e^{s_i - s_{max}}}{\sum_i e^{s_i - s_{max}}}$$

For a pretrained model, cluster scores range from 0 to 16. Because exponential functions increase rapidly, the *softsub* approach will effectively favor the 1-5 clusters with largest scores. We conjecture that a smoother function may lead to better model behavior during active learning, and implement a *softdiv* conversion approach, in which, instead of subtracting the maximum cluster score, we divide by it:

$$f_i^{\text{softdiv}}(s_i) = \frac{e^{s_i / s_{max}}}{\sum_i e^{s_i / s_{max}}}$$

Empirically, this approach leads even to a narrower distribution of sampling fractions than that obtained with the *linear* conversion approach. We introduce a hyperparameter $divf \in (0,1]$ by which we multiply the s_{max} value prior to dividing by it:

$$f_i^{\text{softdivf}}(s_i) = \frac{e^{\frac{s_i}{divf \times s_{max}}}}{\sum_i e^{\frac{s_i}{divf \times s_{max}}}}$$

By visualizing the distribution of *softdiv* values with different values of the hyperparameter (Figure S4.1), we pick $divf = 0.25$, as it maximizes the spread in sampling fractions. In what follows, the *softdiv* conversion will refer to *softdiv* with $divf = 0.25$.

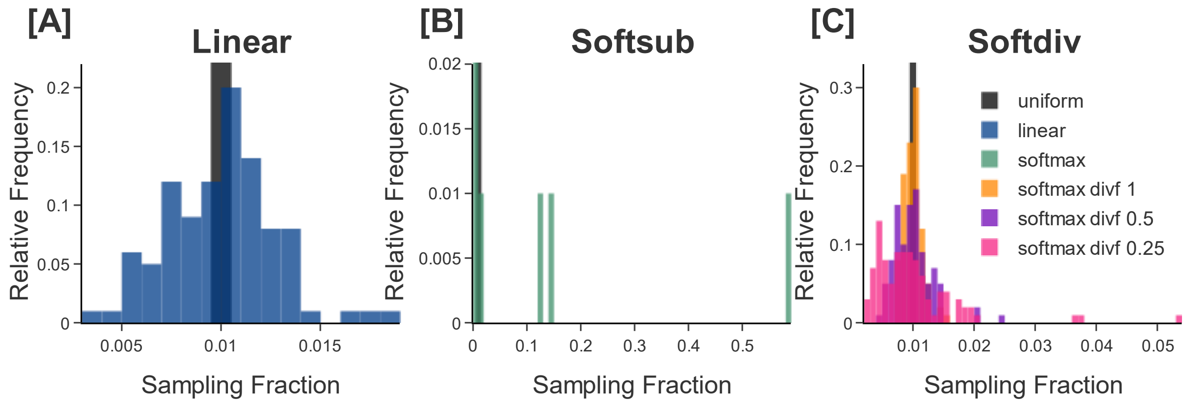

Figure S3.1. Distribution of sampling fractions obtained with different conversion approaches applied to cluster scores obtained from generations of the pretrained model. A bar corresponding to sampling the same number of molecules from each cluster (i.e., uniform sampling) is shown in black.

Section 4: Evolution of the distribution of attractive interaction scores for different conversion methods

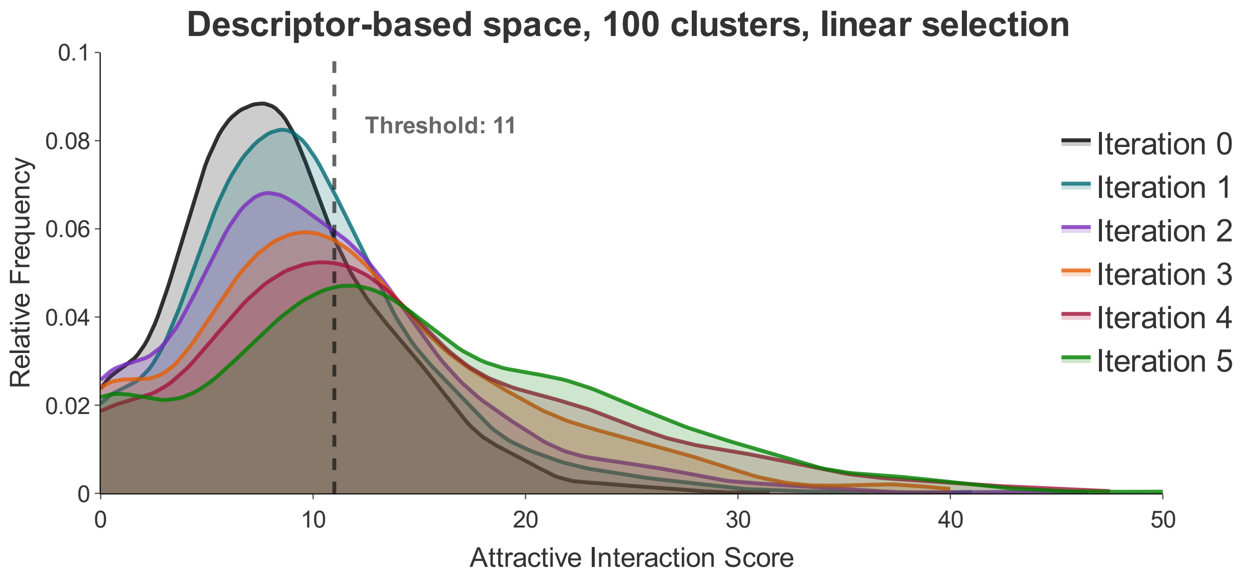

Figure S4.1. Attractive interaction scores for molecules generated by the pretrained model (iteration 0) and by the model after each of the five iterations of active learning where, prior to sampling for docking, molecules in the chemical space are grouped into 100 clusters, and cluster scores are converted into sampling fractions using the *linear* approach.

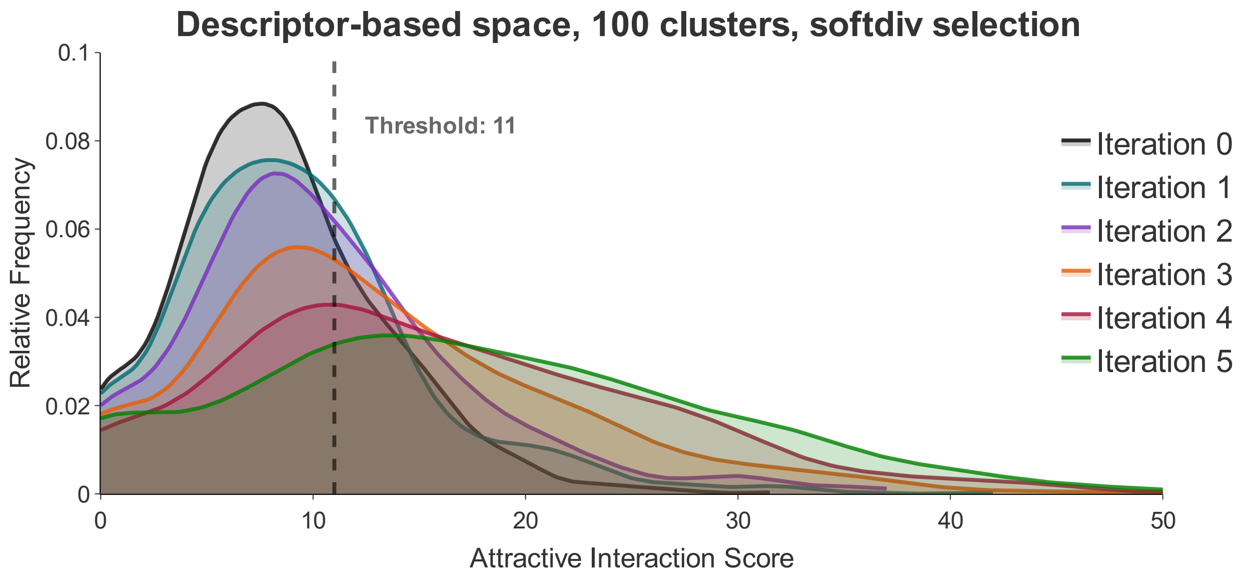

Figure S4.2. Attractive interaction scores for molecules generated by the pretrained model (iteration 0) and by the model after each of the five iterations of active learning where, prior to sampling for docking, molecules in the chemical space are grouped into 100 clusters, and cluster scores are converted into sampling fractions using the *softdiv* approach.

Table S4-3. Statistics of the distribution of attractive interaction scores, when molecules are selected randomly (naïve active learning), with no clustering.

Iteration	Percent > 11	Q1	Q2	Mean	Q3	Max	Std
0	26.20	5.50	8.00	8.41	11.00	33.00	4.58
1	32.40	5.50	9.00	9.27	11.50	35.00	4.79
2	35.00	6.50	9.00	9.67	12.50	33.00	4.89
3	40.00	6.88	9.50	10.45	13.50	37.00	5.31
4	44.80	7.00	10.00	11.03	13.63	42.00	6.16
5	44.20	7.00	10.00	11.13	13.50	38.50	6.05

[a] The percentage of generated molecules with attractive interaction scores equal to or above our score threshold is shown (Percent > 11), as well as the score at the first quartile (Q1), second quartile (Q2), Mean, third quartile (Q3), maximum (Max), and standard deviation (Std) of the distribution.

[b] Iteration 0 refers to the pretraining phase, while later iterations refer to the active learning phases.

[c] This table corresponds to the distribution in Figure 4A of main text.

Table S4.4. Statistics of the distribution of attractive interaction scores, when molecules are clustered into 100 groups and cluster scores are sampled *uniformly*.

Iteration	Percent > 11	Q1	Q2	Mean	Q3	Max	Std
0	12.00	6.68	8.58	8.43	9.85	15.80	2.31
1	24.00	7.75	9.10	9.17	10.88	20.80	3.40
2	30.00	8.17	9.40	9.96	11.50	21.50	3.41
3	35.00	7.86	10.07	10.23	11.63	24.00	3.97
4	50.00	8.88	10.89	11.28	13.46	25.45	4.11
5	50.00	8.80	10.94	11.84	14.48	25.80	4.63

[a] The percentage of generated molecules with attractive interaction scores equal to or above our score threshold is shown (Percent > 11), as well as the score at the first quartile (Q1), second quartile (Q2), Mean, third quartile (Q3), maximum (Max), and standard deviation (Std) of the distribution.

[b] Iteration 0 refers to the pretraining phase, while later iterations refer to the active learning phases.

[c] This table corresponds to the distribution in Figure 4B of main text.

Table S4.5. Statistics of the distribution of attractive interaction scores, when molecules are clustered into 100 groups and cluster scores are converted into sampling fractions using the *linear* method.

Iteration	Percent > 11	Q1	Q2	Mean	Q3	Max	Std
0	12.00	6.68	8.58	8.43	9.85	15.80	2.31
1	23.23	7.88	9.20	9.54	10.71	19.95	2.73
2	37.00	7.78	9.98	10.24	12.10	26.25	4.40
3	47.00	8.29	10.55	11.60	14.51	23.90	5.09
4	62.00	10.19	13.27	13.19	15.84	31.41	5.09
5	71.00	10.50	13.43	14.00	16.93	29.50	5.50

[a] The percentage of generated molecules with attractive interaction scores equal to or above our score threshold is shown (Percent > 11), as well as the score at the first quartile (Q1), second quartile (Q2), Mean, third quartile (Q3), maximum (Max), and standard deviation (Std) of the distribution.

[b] Iteration 0 refers to the pretraining phase, while later iterations refer to the active learning phases.

[c] This table corresponds to the distribution in Figure S4.1.

Table S4.6. Statistics of the distribution of attractive interaction scores, when molecules are clustered into 100 groups and cluster scores are converted into sampling fractions using the *softdiv* method.

Iteration	Percent > 11	Q1	Q2	Mean	Q3	Max	Std
0	28.10	5.50	8.00	8.46	11.50	31.50	4.89
1	34.70	5.50	8.50	9.29	12.50	42.00	5.89
2	42.20	6.50	9.50	10.54	13.50	37.00	6.64
3	54.20	7.50	11.50	13.07	18.00	55.00	8.64
4	65.90	8.50	14.25	15.82	22.00	56.50	9.99
5	71.20	9.50	16.00	17.32	24.50	51.00	10.90

[a] The percentage of generated molecules with attractive interaction scores equal to or above our score threshold is shown (Percent > 11), as well as the score at the first quartile (Q1), second quartile (Q2), Mean, third quartile (Q3), maximum (Max), and standard deviation (Std) of the distribution.

[b] Iteration 0 refers to the pretraining phase, while later iterations refer to the active learning phases.

[c] This table corresponds to the distribution in Figure S4.2.

<u>Section 5:</u> <u>Evolution of the distribution of the number of attractive interactions for different conversion methods</u>

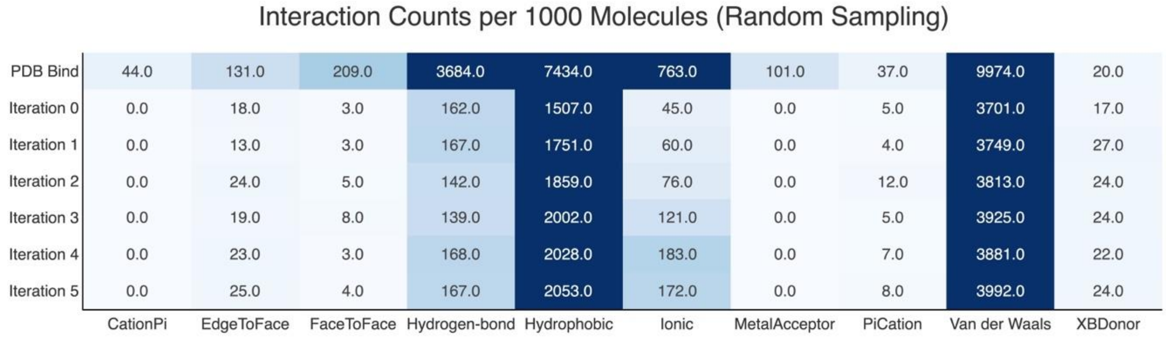

Figure S5.1. Counts of interactions of each type for 1000 scored molecules generated by the pretrained model (iteration 0) and by the model after each of the five rounds of naïve active learning with *random sampling*. A count of interactions from 1000 protein-ligand complexes randomly sampled from the refined set of PDBbind v2020 is included for comparison. These counts correspond to the score distribution in Figure 4A of main text.

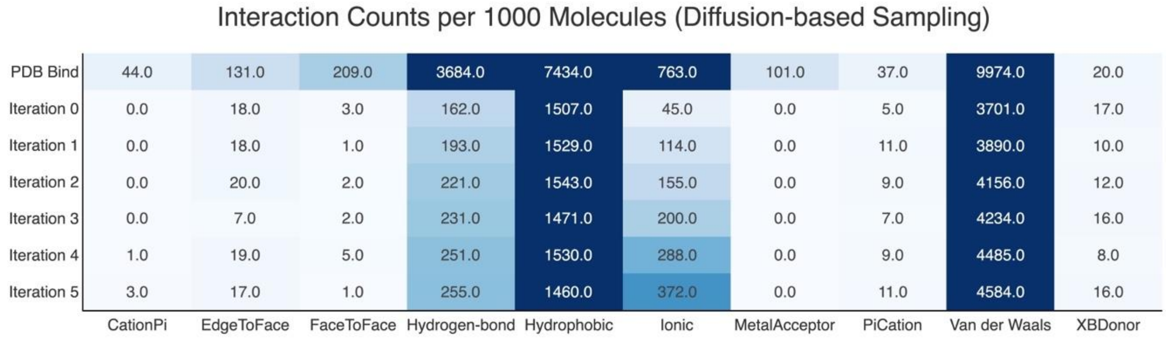

Figure S5.2. Counts of interactions of each type for 1000 molecules generated by the pretrained model (iteration 0) and by the model after each of the five rounds of active learning with clustering into 100 groups and *uniform* selection from each cluster. A count of interactions from 1000 protein-ligand complexes randomly sampled from the refined set of PDBbind v2020 is included for comparison. These counts correspond to the score distribution in Figure 4B of main text.

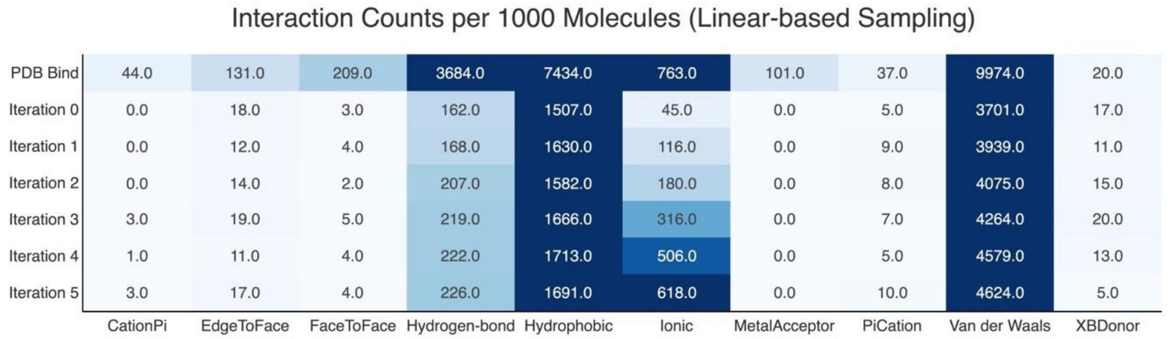

Figure S5.3. Counts of interactions of each type for 1000 scored molecules generated by the pretrained model (iteration 0) and by the model after each of the five rounds of active learning with clustering into 100 groups and conversion of cluster scores into sampling fractions using the *linear* method. A count of interactions from 1000 protein-ligand complexes randomly sampled from the refined set of PDBbind v2020 is included for comparison. These counts correspond to the score distribution in Figure S4.1.

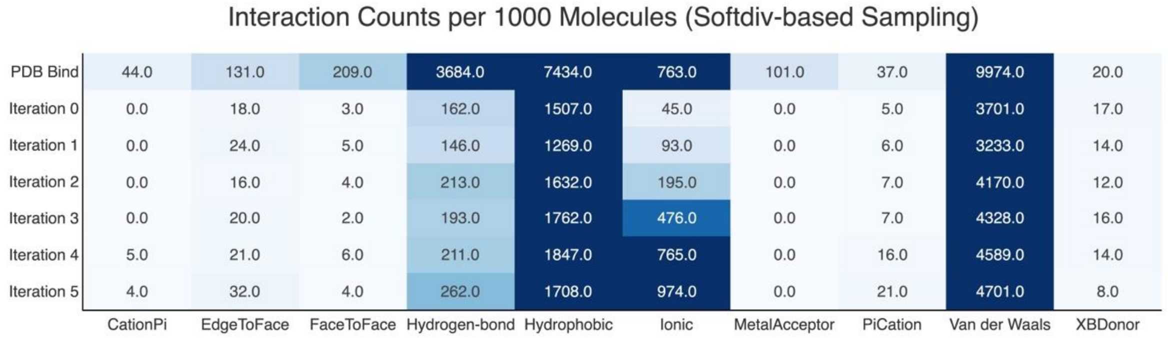

Figure S5.4. Counts of interactions of each type for 1000 scored molecules generated by the pretrained model (iteration 0) and by the model after each of the five rounds of active learning with clustering into 100 groups and conversion of cluster scores into sampling fractions using the *softdiv* method. A count of interactions from 1000 protein-ligand complexes randomly sampled from the refined set of PDBbind v2020 is included for comparison. These counts correspond to the score distribution in Figure S4.2.

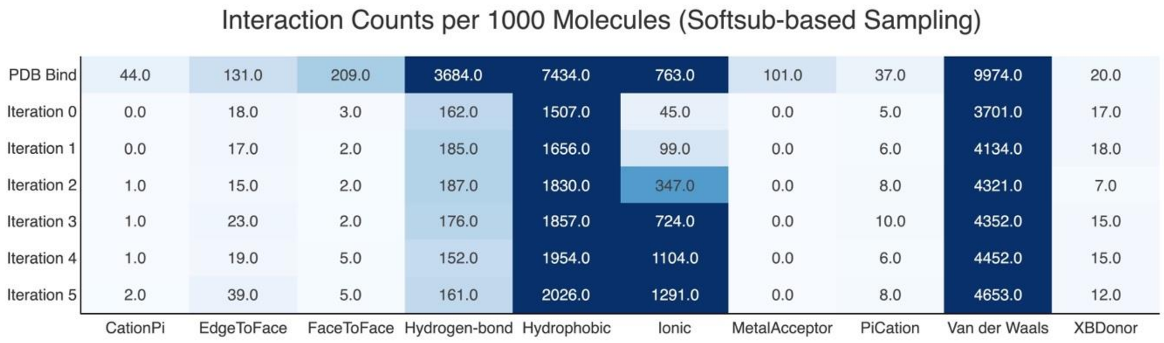

Figure S5.5. Counts of interactions of each type for 1000 molecules generated by the pretrained model (iteration 0) and by the model after each of the five rounds of active learning with clustering into 100 groups and conversion of cluster scores into sampling fractions using the *softsub* method. A count of interactions from 1000 protein-ligand complexes randomly sampled from the refined set of PDBbind v2020 is included for comparison. These counts correspond to the score distribution in Figure 4C of the main text.

Section 6: Evolution of the distribution of cluster-average attractive interaction scores for different conversion methods

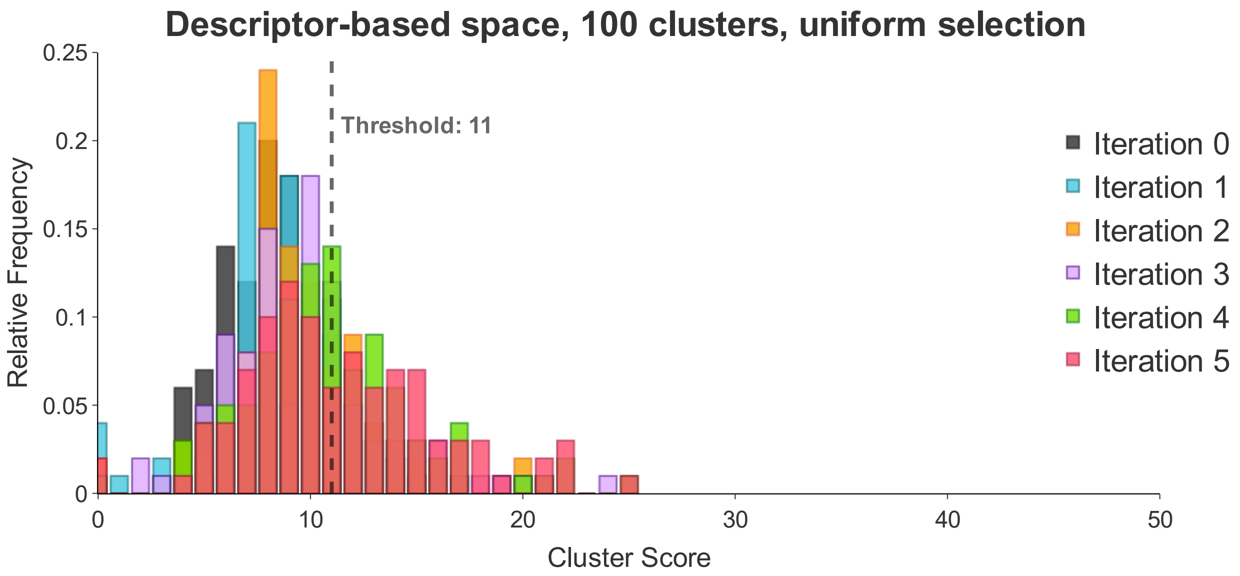

Figure S6.1. Cluster scores (obtained as an average of attractive interaction scores for molecules in the cluster) for molecules generated by the pretrained model (iteration 0) and by the model after each of the five iterations of active learning where, prior to sampling for docking, molecules in the chemical space are grouped into 100 clusters, and molecules are sampled from each cluster *uniformly*. These cluster scores correspond to score distribution in Figure 4B of the main text.

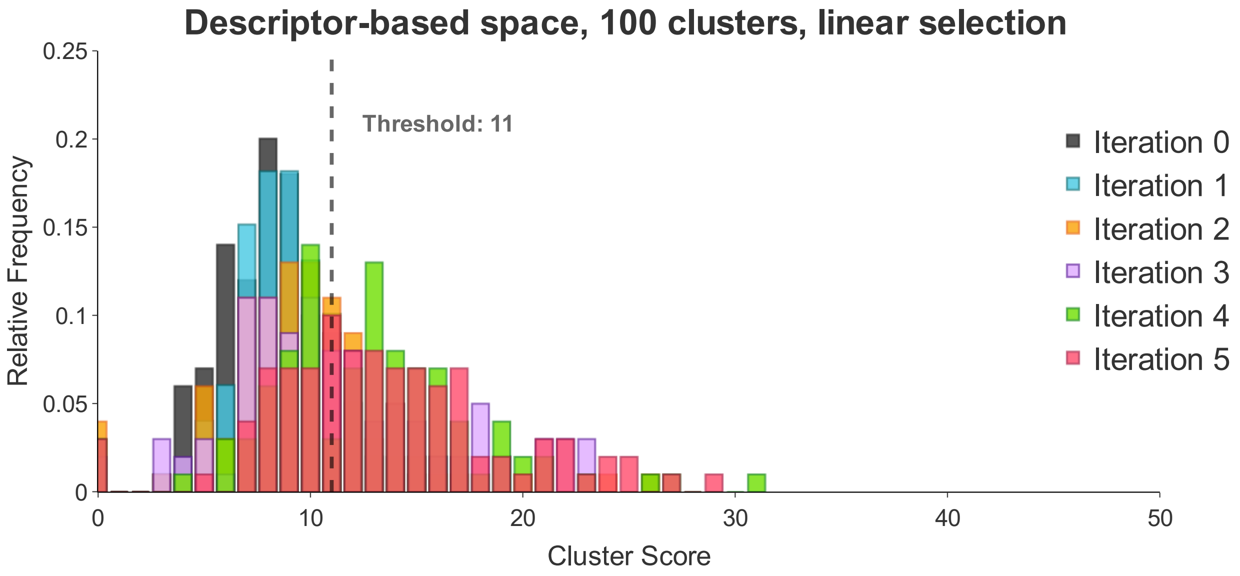

Figure S6.2. Cluster scores (obtained as an average of attractive interaction scores for molecules in the cluster) for molecules generated by the pretrained model (iteration 0) and by the model after each of the five iterations of active learning where, prior to sampling for docking, molecules in the chemical space are grouped into 100 clusters, and molecules are sampled from each cluster using the *linear* method. These cluster scores correspond to score distribution in Figure SI4.1.

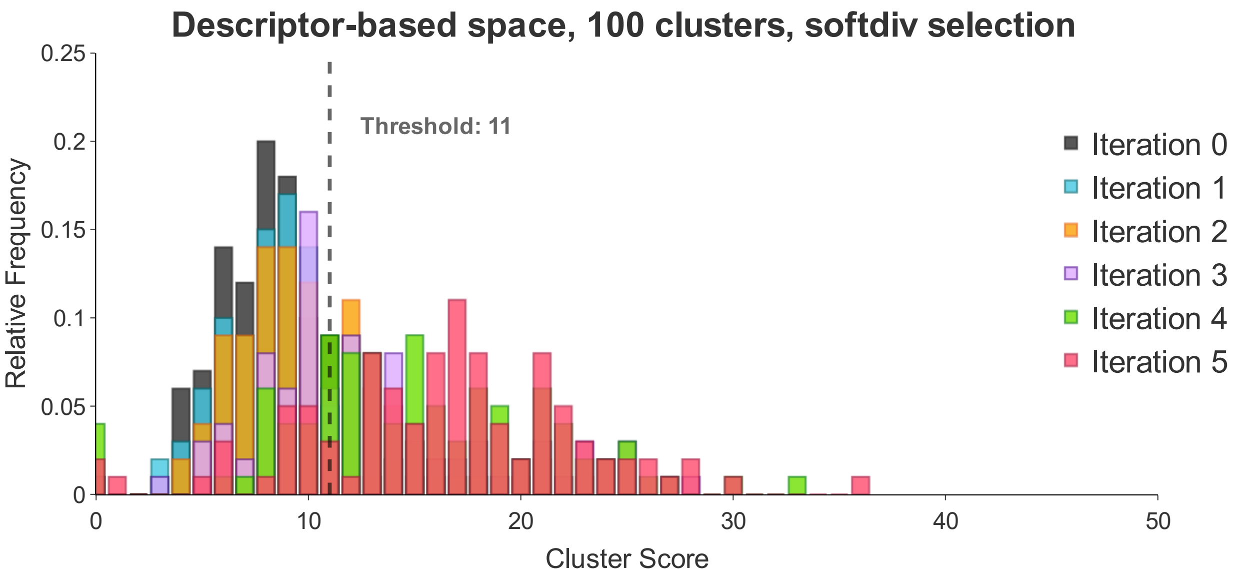

Figure S6.3. Cluster scores (obtained as an average of attractive interaction scores for molecules in the cluster) for molecules generated by the pretrained model (iteration 0) and by the model after each of the five iterations of active learning where, prior to sampling for docking, molecules in the chemical space are grouped into 100 clusters, and molecules are sampled from each cluster using the *softdiv* method. These cluster scores correspond to score distribution in Figure SI4.2.

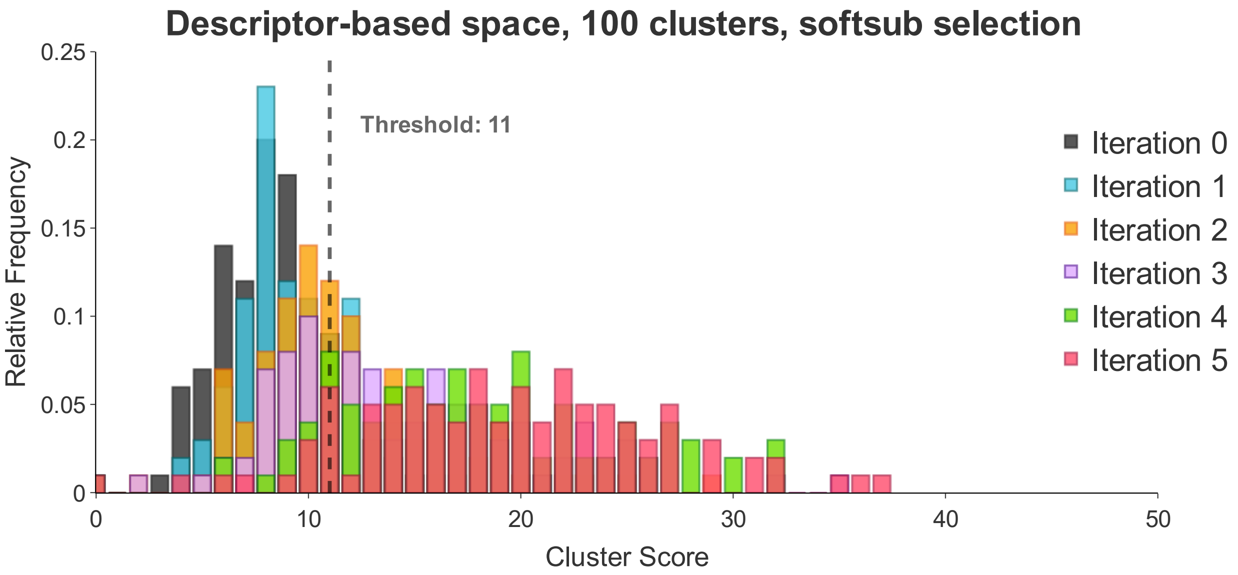

Figure S6.4. Cluster scores (obtained as an average of attractive interaction scores for molecules in the cluster) for molecules generated by the pretrained model (iteration 0) and by the model after each of the five iterations of active learning where, prior to sampling for docking, molecules in the chemical space are grouped into 100 clusters, and molecules are sampled from each cluster using the *softsub* method. These cluster scores correspond to score distribution in Figure 4C of the main text.

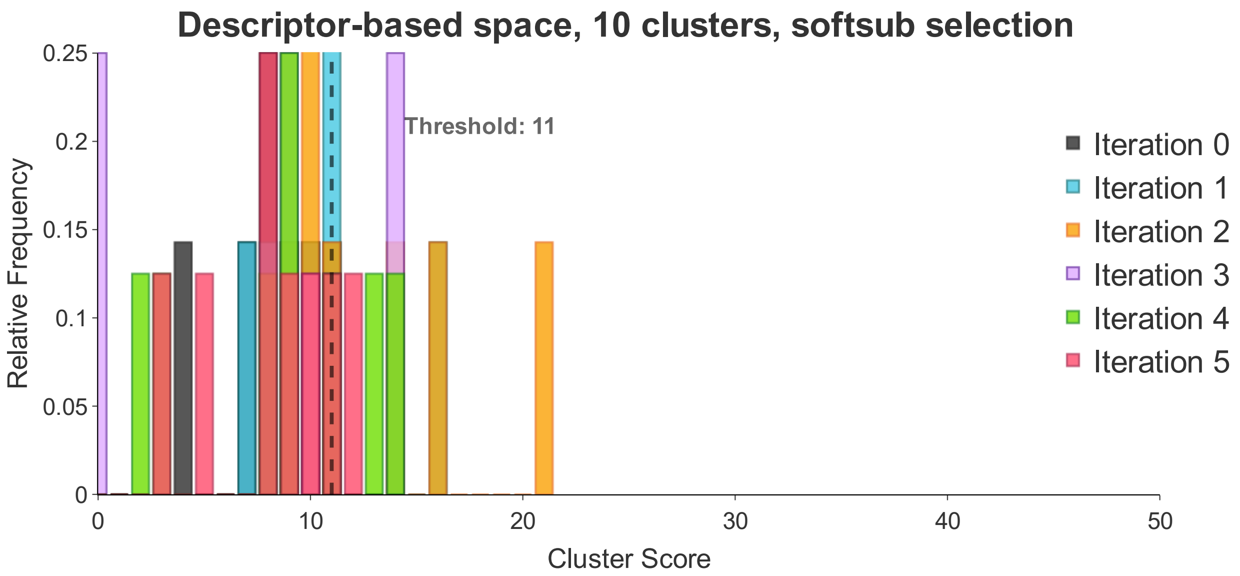

Figure S6.5. Cluster scores (obtained as an average of attractive interaction scores for molecules in the cluster) for molecules generated by the pretrained model (iteration 0) and by the model after each of the five iterations of active learning where, prior to sampling for docking, molecules in the chemical space are grouped into 10 clusters, and molecules are sampled from each cluster using the *softsub* method. These cluster scores correspond to score distribution in Figure S10.1 of the main text.

Section 7: Performance on the MOSES benchmark

Table S7.1. Primary results of our pretrained model on the MOSES benchmark compared to top-performing models in the field.

Model	Validity	Unique@1K	Unique@10K	Novelty	$IntDiv_1$	$IntDiv_2$	Filters
Our Model	0.996	**1.000**	0.999	0.730	0.856	0.850	**0.998**
MolGPT[69]	0.994	N/A	**1.000**	0.797	0.857	0.851	N/A
LatentGAN[70]	0.897	**1.000**	0.997	0.949	0.857	0.850	0.973
JT-VAE[50]	**1.000**	**1.000**	**1.000**	0.914	0.855	0.849	0.976
CharRNN[5]	0.975	**1.000**	0.999	0.842	0.856	0.850	0.994
MolecularRNN[26]	**1.000**	N/A	0.994	**1.000**	0.881	**0.876**	N/A
iPPIgAN[33]	0.989	**1.000**	0.999	0.990	N/A	N/A	N/A
DNMG[34]	0.999	**1.000**	0.998	0.936	0.856	0.850	0.996
CogMol[43]	0.955	**1.000**	**1.000**	N/A	0.857	0.851	0.989
TransVAE[51]	0.567	NA	N/A	0.996	N/A	N/A	N/A
ShapeProb[57]	0.969	**1.000**	0.995	N/A	0.865	N/A	0.865
GENTRL[54]	0.850	N/A	N/A	N/A	N/A	N/A	N/A
TransAntivirus[62]	**1.000**	0.999	0.999	0.999	**0.895**	N/A	N/A
CRTmaccs[64]	**1.000**	**1.000**	**1.000**	**1.000**	N/A	N/A	N/A
MolGCT[66]	0.985	**1.000**	0.998	0.814	0.853	N/A	0.996
cMolGPT[99]	0.988	**1.000**	0.999	N/A	N/A	N/A	N/A
GraphINVENT[77]	0.964	**1.000**	0.998	N/A	0.857	0.851	0.950
cTransformer[67]	0.988	**1.000**	0.999	N/A	N/A	N/A	N/A
GMTransformer[63]	0.829	**1.000**	**1.000**	0.883	0.856	N/A	0.980

[a] Validity (ratio of generated molecules deemed valid by RDKit's molecular structure parser), Unique@1K and @10K (fraction of valid generated molecules with no duplicates), Novelty (fraction of valid and unique generated molecules that are not in the training set), $IntDiv_i$ (internal diversity within the generated set for power mean i), and Filters (fraction of generated molecules that pass filters that check for specific fragments) are shown. See MOSES benchmark[91] for more details on how these metrics are calculated.
[b] The top value for each metric is shown in bold.
[c] Values not reported are shown as N/A.

Table S7.2. Additional results of our pretrained model on the MOSES benchmark compared to top-performing models in the field.

Model	FCD/Test	FCD/TestSF	Frag/Test	Frag/TestSF	SNN/Test	SNN/TestSF	Scaff/Test	Scaff/TestSF
Our Model	**0.038**	**0.450**	**1.000**	**0.999**	0.633	**0.585**	**0.970**	0.071
MolGPT[69]	0.067	0.507	N/A	N/A	N/A	N/A	N/A	N/A
LatentGAN[70]	0.296	0.824	0.999	0.998	0.538	0.514	0.886	0.100
JT-VAE[50]	0.395	0.938	0.997	0.995	0.548	0.519	0.896	0.101
CharRNN[5]	0.073	0.520	**1.000**	0.998	0.601	0.565	0.924	0.110
MolecularRNN[26]	N/A	N/A	N/A	N/A	N/A	N/A	N/A	N/A
iPPIgAN[33]	5.879	6.171	N/A	N/A	N/A	N/A	N/A	N/A
DNMG[34]	0.373	0.631	0.999	0.998	0.472	0.579	0.784	**0.998**
CogMol[43]	0.166	0.603	0.999	0.997	0.560	0. 533	0.905	0.128
TransVAE[51]	N/A	N/A	N/A	N/A	N/A	N/A	N/A	N/A
ShapeProb[57]	1.332	1.850	0.984	0.980	0.446	0.432	0.459	0.066
GENTRL[54]	N/A	N/A	N/A	N/A	N/A	N/A	N/A	N/A
TransAntivirus[62]	10.947	N/A	N/A	N/A	N/A	N/A	N/A	N/A
CRTmaccs[64]	13.565	13.999	N/A	N/A	0.334	0.330	N/A	N/A
MolGCT[66]	0.402	0.803	0.997	0.995	0.618	0.577	0.891	0.092
cMolGPT[99]	N/A	N/A	**1.000**	0.998	0.619	0.578	N/A	N/A
GraphINVENT[77]	0.682	1.223	0.986	0.986	0.569	0.539	0.885	0.127
cTransformer[67]	N/A	N/A	**1.000**	0.998	0.619	0.578	N/A	N/A
GMTransformer[63]	0.199	0.760	0.998	0.996	0.578	0.546	0.913	0.109

[a] FCD (Fr**échet ChemNet Distance that is calculated using activation of the penultimate layer of ChemNet), Frag (compares molecular fragments between generated and training sets), SNN (average Tanimoto similarity between molecules in the generated set and the corresponding nearest molecule in the training set), and Scaff (compares molecular scaffolds between generated and training sets) are shown**. Test (similarity from the training set to the test set) and TestSF (similarity from the training set to the scaffold test set) are shown for each metric. See MOSES benchmark[91] for more details on how these metrics are calculated

[b] The top value for each metric is shown in bold.

[c] Values not reported are shown as N/A

Section 8: Details on the execution of t-distributed stochastic neighbor embedding (t-SNE)

To create a standard t-SNE space which involves a constant coordinate system, we proceed as follows. Firstly, we collect scored molecules from all iterations (6,000 molecules). Secondly, we add the molecules from all active learning training sets that employed either softmax or uniform selection methods. Thirdly, we add a random sample of 10,000 molecules from the set of generations at each iteration. We perform this sampling to have the same number of molecules from the active learning training sets and generated sets, which enables us to fairly compute the difference in distributions. Note that our training sets usually contain slightly more than 10,000 molecules, so we sample exactly 10,000 for consistency. After combining all molecules and dropping all duplicates, we perform a t-SNE reduction.

Section 9: Vocabulary composition of the combined dataset

Table S9.1. List of unique tokens that occur in the unfiltered combined dataset less than 1,000 times.

- '%10'
- '%11'
- '%12'
- '%13'
- '%14'
- '%15'
- '%16'
- '%17'
- '%18'
- '%19'
- '%20'
- '%21'
- '%22'
- '%23'
- '%24'
- '%25'
- '%26'
- '%27'
- '%28'
- '%29'
- '%30'
- '%31'
- '%32'
- '*'
- ':'
- '[*]'
- '[10B]'
- '[11C-]'
- '[11C@@H]'
- '[11CH2]'
- '[11CH3]'
- '[11CH]'
- '[11C]'
- '[11c]'
- '[123I-]'
- '[123IH]'
- '[123I]'
- '[123Te]'
- '[124I-]'
- '[124I]'
- '[125I-]'
- '[125IH]'
- '[125I]'
- '[127I]'
- '[127Xe]'
- '[129Xe]'
- '[131Cs]'
- '[131I-]'
- '[131I]'
- '[133Xe]'
- '[135I]'
- '[13CH2]'
- '[13CH3]'
- '[13CH]'
- '[13C]'
- '[13NH3]'
- '[13cH]'
- '[13c]'
- '[14C@@H]'
- '[14C@@]'
- '[14C@H]'
- '[14CH2]'
- '[14CH3]'
- '[14CH]'
- '[14C]'
- '[14cH]'
- '[14c]'
- '[15NH]'
- '[15OH2]'
- '[15nH]'
- '[15n]'
- '[17F]'
- '[18F-]'
- '[18FH]'
- '[18F]'
- '[18OH]'
- '[18O]'
- '[19F]'
- '[211At]'
- '[223Ra]'
- '[22Na+]'
- '[32PH]'
- '[32P]'
- '[35S]'
- '[3H]'
- '[42K+]'
- '[45Ca+2]'
- '[47Ca+2]'
- '[4H]'
- '[73Se]'
- '[75Se]'
- '[76BrH]'
- '[76Br]'
- '[81Kr]'
- '[82Rb+]'
- '[82Rb]'
- '[85Sr+2]'
- '[85SrH2]'
- '[89Sr+2]'
- '[Ag+]'
- '[Ag-4]'
- '[Ag-]'
- '[Ag]'
- '[Al+3]'
- '[Al-3]'
- '[Al]'
- '[Ar]'
- '[As+]'
- '[As-]'
- '[AsH3]'
- '[AsH]'
- '[As]'
- '[At]'
- '[Au-]'
- '[Au]'
- '[B@-]'
- '[B@@-]'
- '[BH-]'
- '[BH2-]'
- '[BH3-]'
- '[B]'
- '[Ba+2]'
- '[Ba]'
- '[Be+2]'
- '[Bi+3]'
- '[BiH3]'
- '[Bi]'
- '[Br+2]'
- '[Br]'
- '[C+]'
- '[CH+]'
- '[CH-]'
- '[CH2+]'
- '[CH2-]'
- '[CH2]'
- '[CH]'
- '[C]'
- '[Ca++]'
- '[Ca+2]'
- '[CaH2]'
- '[Ca]'
- '[Cl+2]'
- '[Cl+3]'
- '[Cl+]'
- '[Cl]'
- '[Co]'
- '[Cs+]'
- '[Cs]'
- '[Cu-]'
- '[Cu]'
- '[F+]'
- '[F-]'
- '[Fe++]'
- '[Fe--]'
- '[Fe-3]'
- '[Fe]'
- '[Gd-4]'
- '[Gd-5]'
- '[H+]'
- '[H-]'
- '[HH]'
- '[He]'
- '[Hg]'
- '[I+2]'

- '[I+3]'
- '[I+]'
- '[IH2]'
- '[IH]'
- '[I]'
- '[KH]'
- '[K]'
- '[Kr]'
- '[Li+]'
- '[LiH]'
- '[Li]'
- '[Mg+2]'
- '[Mg+]'
- '[MgH2]'
- '[Mg]'
- '[Mn]'
- '[Mo]'
- '[N@+]'
- '[N@@+]'
- '[N@@H+]'
- '[N@@]'
- '[N@H+]'
- '[N@]'
- '[NH-]'
- '[NH2+]'
- '[NH4+]'
- '[NH]'
- '[N]'
- '[NaH]'
- '[Na]'
- '[Nb--]'
- '[Ni++]'
- '[Ni]'
- '[O+]'
- '[O-2]'
- '[OH+]'
- '[OH-]'
- '[OH]'
- '[O]'
- '[Os]'
- '[P-]'
- '[P@+]'
- '[P@@+]'
- '[P@@]'
- '[P@]'
- '[PH+]'
- '[PH2+]'
- '[PH2]'
- '[PH]'
- '[P]'
- '[Pd--]'
- '[Pd]'
- '[Pt--]'
- '[Pt]'
- '[Ra]'
- '[Rb+]'
- '[Rb]'
- '[Re-]'
- '[Re]'
- '[Ru-]'
- '[Ru]'
- '[S-2]'
- '[S-]'
- '[S@+]'
- '[S@@+]'
- '[S@]'
- '[SH+]'
- '[SH-]'
- '[SH2]'
- '[SH]'
- '[S]'
- '[Sb]'
- '[Se+]'
- '[Se-2]'
- '[Se-]'
- '[SeH2]'
- '[SeH]'
- '[Si-]'
- '[Si@]'
- '[SiH-]'
- '[SiH2]'
- '[SiH3-]'
- '[SiH3]'
- '[SiH4]'
- '[SiH]'
- '[Sn]'
- '[Sr++]'
- '[Sr+2]'
- '[SrH2]'
- '[Tc]'
- '[Te+]'
- '[Te-]'
- '[TeH2]'
- '[TeH]'
- '[Te]'
- '[V]'
- '[W]'
- '[Xe]'
- '[Zn++]'
- '[Zn+2]'
- '[Zn+]'
- '[Zn-2]'
- '[Zn]'
- '[b-]'
- '[c+]'
- '[c-]'
- '[cH+]'
- '[cH-]'
- '[c]'
- '[n-]'
- '[nH+]'
- '[n]'
- '[o+]'
- '[o]'
- '[s+]'
- '[s]'
- '[se+]'
- '[te+]'
- '[te]'
- 'b'
- 'p'

Table S9.2. List of unique tokens in the filtered combined dataset.

- '!'
- '#'
- '('
- ') '
- '-'
- '.'
- '/'
- '1'
- '2'
- '3'
- '4'
- '5'
- '6'
- '7'
- '8'
- '9'
- '<'
- '='
- 'B'
- 'Br'
- 'C'
- 'Cl'
- 'F'
- 'I'
- 'N'
- 'O'
- 'P'
- 'S'
- '[2H]'
- '[B-]'
- '[Br-]'
- '[C-]'
- '[C@@H]'
- '[C@@]'
- '[C@H]'
- '[C@]'
- '[Cl-]'
- '[H]'
- '[I-]'
- '[K+]'
- '[N+]'
- '[N-]'
- '[NH+]'
- '[NH3+]'
- '[Na+]'
- '[O-]'
- '[P+]'
- '[S+]'
- '[S@@]'
- '[Se]'
- '[Si]'
- '[n+]'
- '[nH]'
- '[se]'
- '\'
- 'c'
- 'n'
- 'o'
- 's'
- '~'

Section 10: Choosing the number of clusters to use for k-means

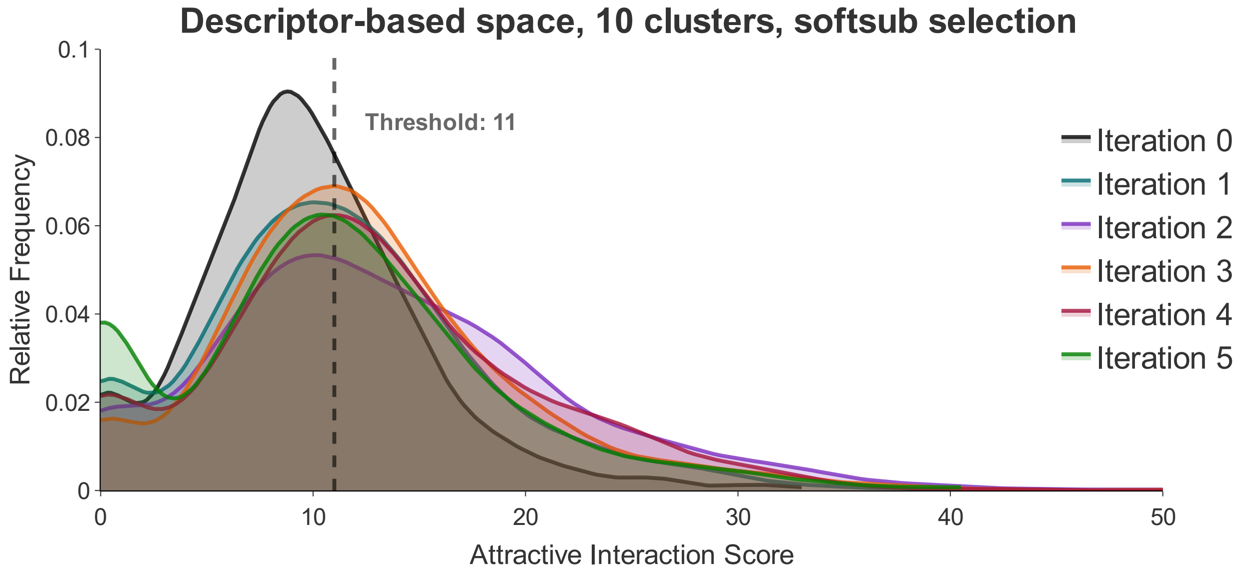

Figure S10.1. Attractive interaction scores for molecules generated by the pretrained model (iteration 0) and by the model after each of the five iterations of active learning where, prior to sampling for docking, molecules in the chemical space are grouped into 10 clusters. Cluster scores are converted into sampling fractions using the *softsub* approach.

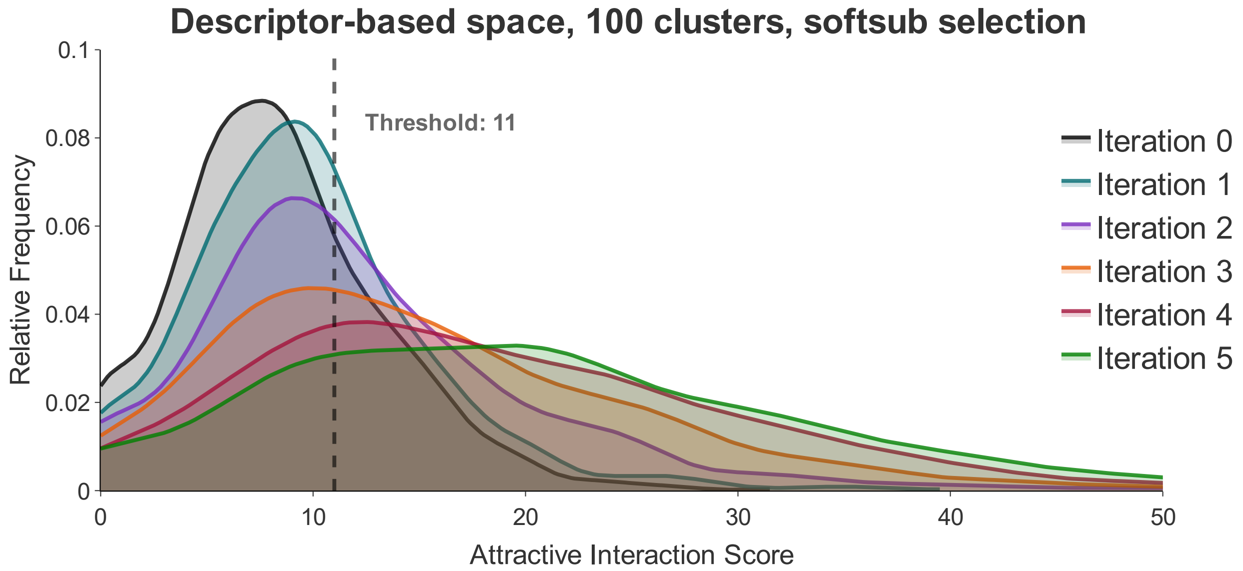

Figure S10.2. Attractive interaction scores for molecules generated by the pretrained model (iteration 0) and by the model after each of the five iterations of active learning where, prior to sampling for docking, molecules in the chemical space are grouped into 100 clusters. Cluster scores are converted into sampling fractions using the *softsub* approach. This figure occurs in the main text (Figure 4C), but is also shown here for comparison.

Table S10.3. Statistics of the distribution of attractive interaction scores, when molecules are clustered into 10 groups and cluster scores are converted into sampling fractions using the *softsub* method.

Iteration	Percent > 11	Q1	Q2	Mean	Q3	Max	Std
0	14.29	8.26	8.91	8.75	10.28	11.25	2.25
1	42.86	8.96	10.27	10.67	11.49	16.09	2.62
2	57.14	10.32	11.51	13.31	15.34	21.00	3.91
3	37.50	6.80	10.45	8.88	12.51	14.94	5.44
4	37.50	7.33	9.10	9.03	11.74	14.12	3.83
5	25.00	7.50	8.75	8.67	10.99	12.90	2.96

[a] The percentage of generated molecules with attractive interaction scores equal to or above our score threshold is shown (Percent > 11), as well as the score at the first quartile (Q1), second quartile (Q2), Mean, third quartile (Q3), maximum (Max), and standard deviation (Std) of the distribution.

[b] Iteration 0 refers to the pretraining phase, while later iterations refer to the active learning phases.

Table S10.4. Statistics of the distribution of attractive interaction scores, when molecules are clustered into 100 groups and cluster scores are converted into sampling fractions using the *softsub* method.

Iteration	Percent > 11	Q1	Q2	Mean	Q3	Max	Std
0	28.10	5.50	8.00	8.46	11.50	31.50	4.89
1	37.00	6.00	9.00	9.76	12.50	39.50	5.63
2	49.70	7.50	10.50	12.22	16.00	51.00	7.93
3	62.60	8.00	13.50	15.14	20.63	54.00	9.70
4	72.90	10.00	16.50	18.25	25.00	55.50	10.90
5	76.00	11.00	19.00	20.08	27.63	59.00	11.90

[a] The percentage of generated molecules with attractive interaction scores equal to or above our score threshold is shown (Percent > 11), as well as the score at the first quartile (Q1), second quartile (Q2), Mean, third quartile (Q3), maximum (Max), and standard deviation (Std) of the distribution.

[b] Iteration 0 refers to the pretraining phase, while later iterations refer to the active learning phases.

Section 11: Additional evaluation of generations across active learning iterations

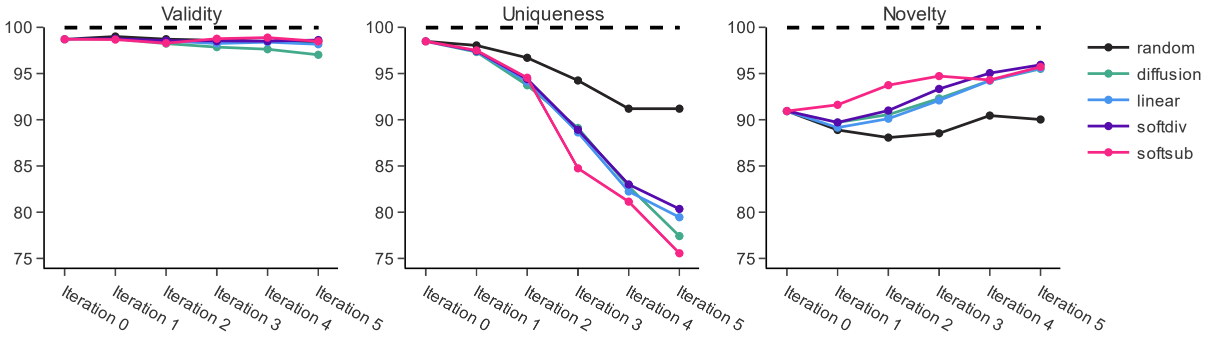

Figure S11.1. Percentage of molecules generated by our model that are valid, unique, or novel after pretraining (iteration 0) and five rounds of active learning. Data are shown for different sampling/conversion schemes.

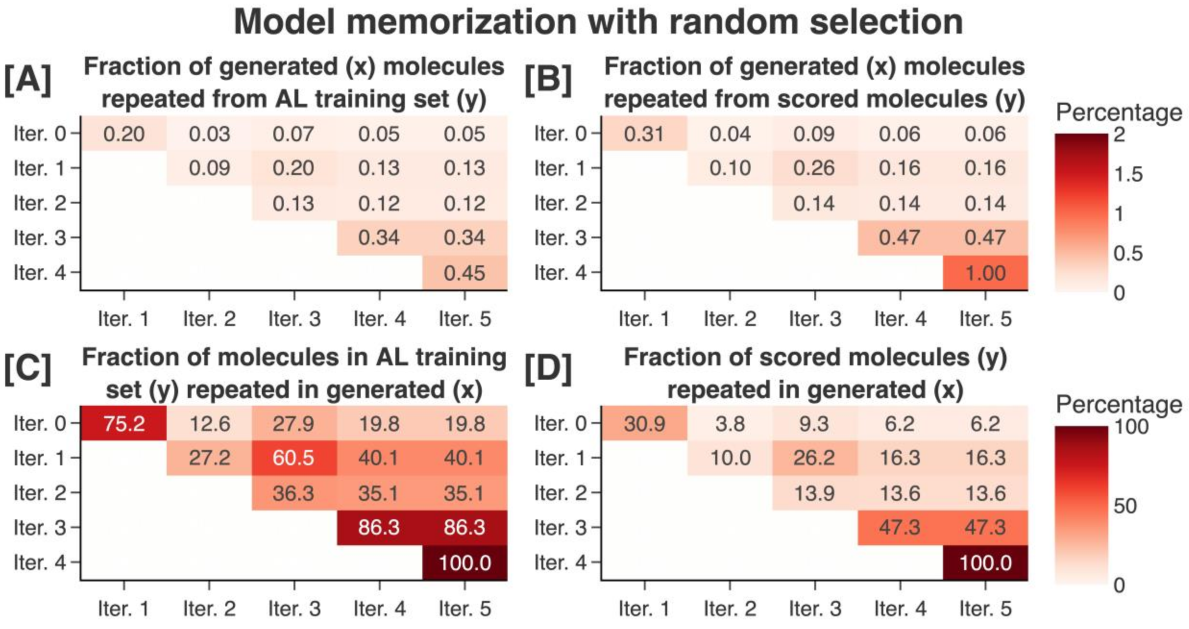

Figure S11.2. Memorization of training set by our model over five rounds of naïve active learning with random selection. (A) The fraction of molecules in a set of 100,000 generated at iteration *i* that occur in the training set at iteration *i*-1. (B) The fraction of molecules in a set of 100,000 generated at iteration *i* that occur in the set of scored molecules at iteration *i*-1. (C) The number of molecules from the active training set at iteration *i*-1 that occurs in generations at iteration *i* divided by the size of the active learning training set at iteration *i*-1. (D) the number of scored molecules at iteration *i*-1 that occur in generations at iteration *i* divided by number of scored molecules at iteration *i*-1 (i.e., 1000).

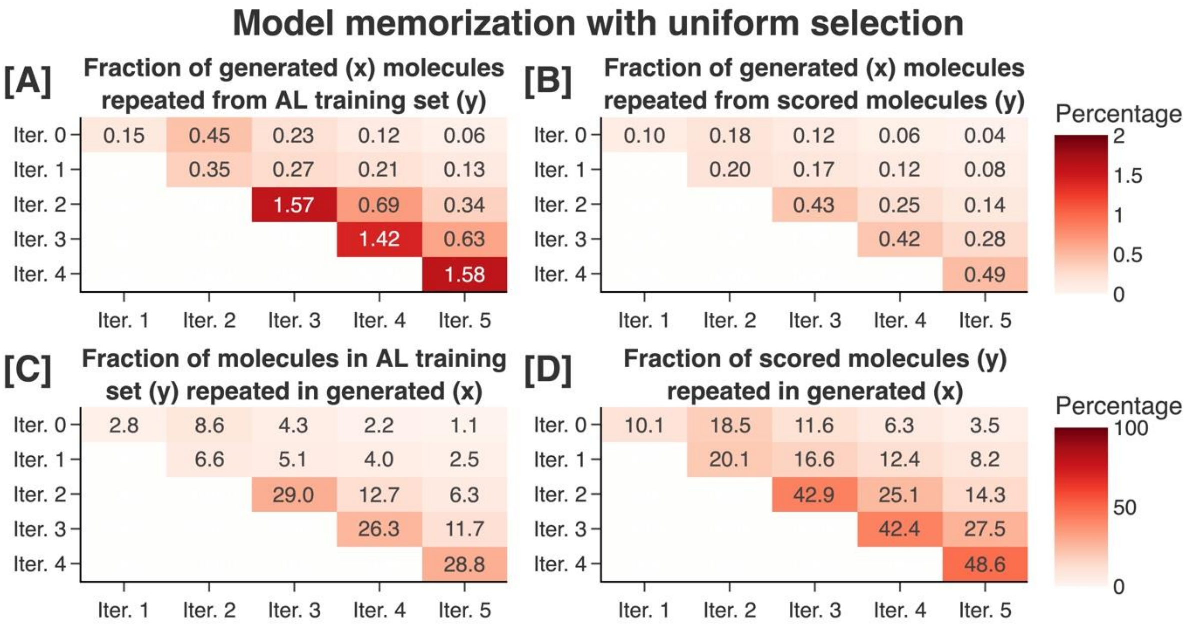

Figure S11.3. Memorization of training set by our model over five rounds of active learning with 100 clusters and uniform selection. (A) The fraction of molecules in a set of 100,000 generated at iteration *i* that occur in the training set at iteration *i*-1. (B) The fraction of molecules in a set of 100,000 generated at iteration *i* that occur in the set of scored molecules at iteration *i*-1. (C) The number of molecules from the active training set at iteration *i*-1 that occurs in generations at iteration *i* divided by the size of the active learning training set at iteration *i*-1. (D) the number of scored molecules at iteration *i*-1 that occur in generations at iteration *i* divided by number of scored molecules at iteration *i*-1 (i.e., 1000).

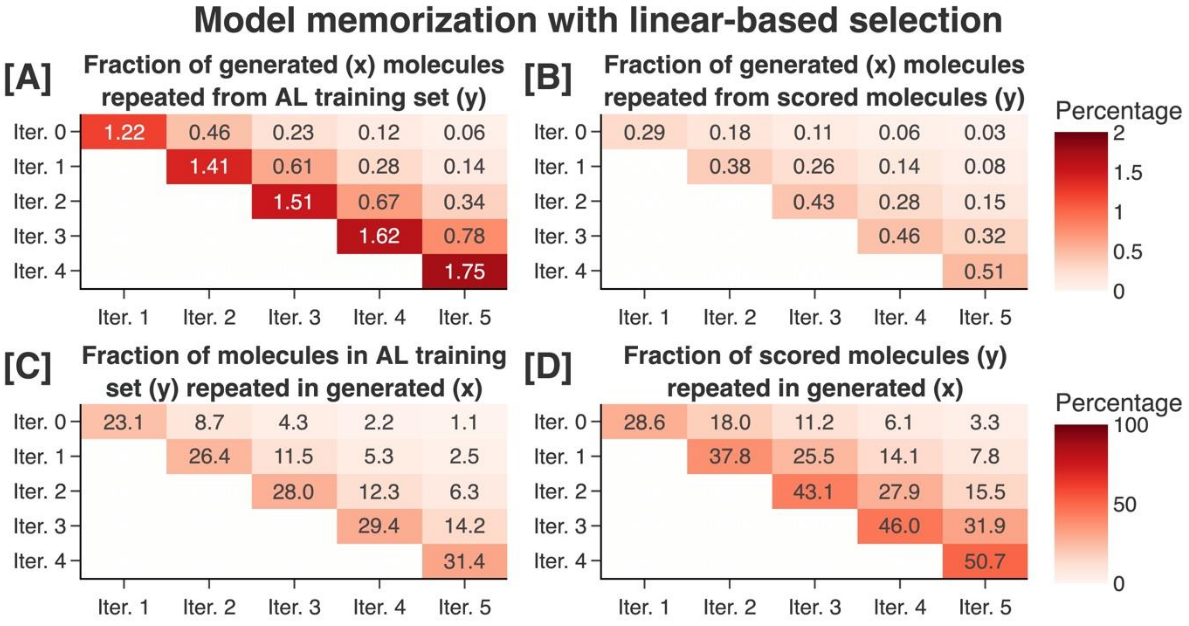

Figure S11.4. Memorization of training set by our model over five rounds of active learning with 100 clusters and linear selection. (A) The fraction of molecules in a set of 100,000 generated at iteration *i* that occur in the training set at iteration *i*-1. (B) The fraction of molecules in a set of 100,000 generated at iteration *i* that occur in the set of scored molecules at iteration *i*-1. (C) The number of molecules from the active training set at iteration *i*-1 that occurs in generations at iteration *i* divided by the size of the active learning training set at iteration *i*-1. (D) the number of scored molecules at iteration *i*-1 that occur in generations at iteration *i* divided by number of scored molecules at iteration *i*-1 (i.e., 1000).

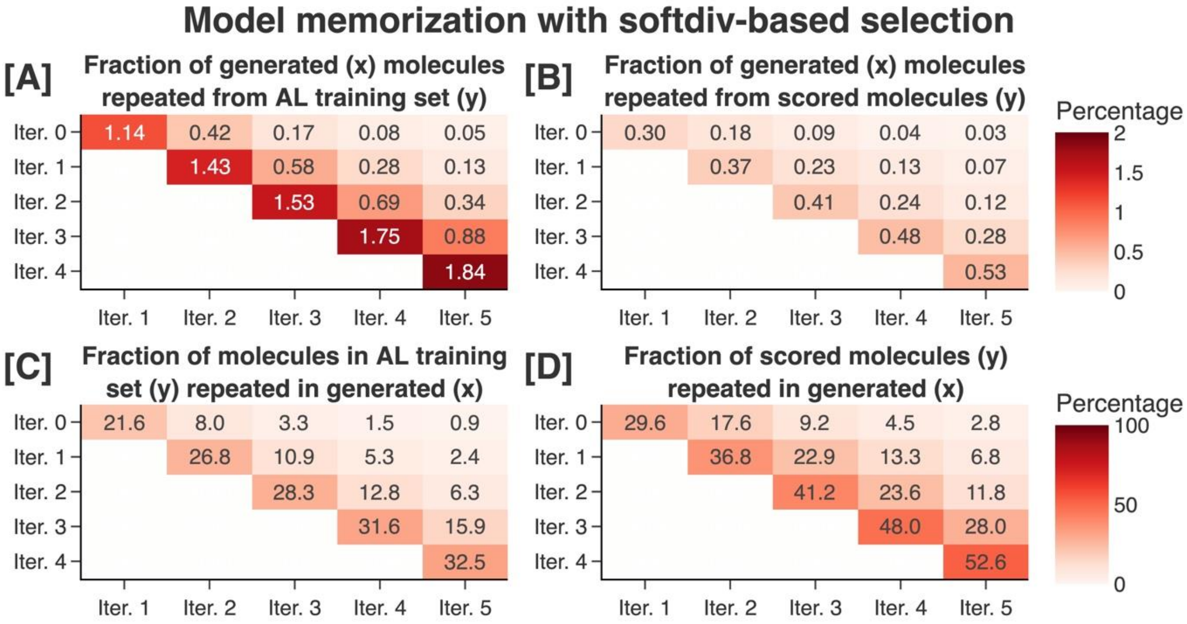

Figure S11.5. Memorization of training set by our model over five rounds of active learning with 100 clusters and softdiv selection. (A) The fraction of molecules in a set of 100,000 generated at iteration *i* that occur in the training set at iteration *i*-1. (B) The fraction of molecules in a set of 100,000 generated at iteration *i* that occur in the set of scored molecules at iteration *i*-1. (C) The number of molecules from the active training set at iteration *i*-1 that occurs in generations at iteration *i* divided by the size of the active learning training set at iteration *i*-1. (D) the number of scored molecules at iteration *i*-1 that occur in generations at iteration *i* divided by number of scored molecules at iteration *i*-1 (i.e., 1000).

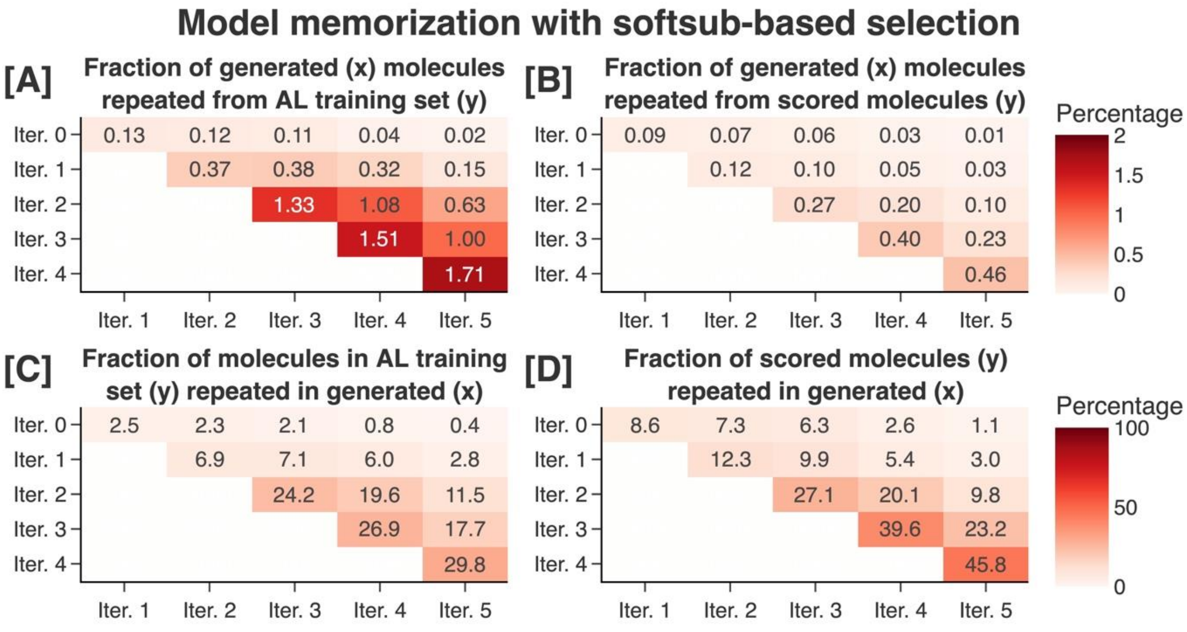

Figure S11.6. Memorization of training set by our model over five rounds of active learning with 100 clusters and softsub selection. (A) The fraction of molecules in a set of 100,000 generated at iteration *i* that occur in the training set at iteration *i*-1. (B) The fraction of molecules in a set of 100,000 generated at iteration *i* that occur in the set of scored molecules at iteration *i*-1. (C) The number of molecules from the active training set at iteration *i*-1 that occurs in generations at iteration *i* divided by the size of the active learning training set at iteration *i*-1. (D) the number of scored molecules at iteration *i*-1 that occur in generations at iteration *i* divided by number of scored molecules at iteration *i*-1 (i.e., 1000).
